# Supplementary material for: The Pre-BRA (pre-pectoral Breast Reconstruction EvAluation) feasibility study: protocol for a mixed-methods IDEAL 2a/2b prospective cohort study to determine the safety and effectiveness of prepectoral implant-based breast reconstruction
Source: BMJ Open. 2020 Jan 26;10(1):e033641. doi: 10.1136/bmjopen-2019-033641 (PMC7044855; doi:10.1136/bmjopen-2019-033641)
Supplement: Supplementary data [file bmjopen-2019-033641supp001.pdf]

The Pre-BRA (Pre-pectoral Breast Reconstruction Evaluation)
Feasibility Study: Protocol
KL Harvey, N Mills, P White, C Holcombe, S Potter
Appendix 1.

Local Trust Logo here

The Pre-BRA Feasibility Study
PATIENT CONSENT FORM

Please initial box

1	I confirm that I have read and understand the information sheet dated 28/03/2019 (version 2.0) for the above study. I have had the opportunity to consider the information, ask questions and have had these answered satisfactorily.	☐
2	I understand that my participation is voluntary and that I am free to withdraw at any time without giving any reason, without my medical care or legal rights being affected.	☐
3	I understand that relevant sections of my medical notes and data collected during the study may be looked at by individuals from regulatory authorities, the study research team or from the NHS Trust, where it is relevant to my taking part in this research. I give permission for these individuals to have access to my records.	☐
4	I agree that the study team may retain personally identifiable data collected during the study which could be used to directly support future research studies such as an extension to the Pre-Bra Study. For example, the study team may wish to contact me with further questionnaires, or to use identifiers such as my individual NHS number to interrogate national databases.	☐
5	I agree that data collected during the study may be retained by the study team for up to 10 years after the study has closed, in order to support future research studies.	☐
6	I agree to complete patient questionnaires for use in the study	☐
7	I agree to take part in the above study	☐
8	I would like to be sent a summary of the study's findings when the research is complete: Yes ☐ No ☐<br>I have supplied my email address for that purpose.<br>Email address: ______________________________	☐

Name of Patient	Date	Signature
Researcher Taking Consent	Date	Signature

One copy of the signed consent form should be given to the participant and one copy retained by the researcher.

IRAS Project ID: 255421 Patient Informed Consent Form v2.0 21/03/2019
