# Supplementary material for: The Pre-BRA (pre-pectoral Breast Reconstruction EvAluation) feasibility study: protocol for a mixed-methods IDEAL 2a/2b prospective cohort study to determine the safety and effectiveness of prepectoral implant-based breast reconstruction
Source: BMJ Open. 2020 Jan 26;10(1):e033641. doi: 10.1136/bmjopen-2019-033641 (PMC7044855; doi:10.1136/bmjopen-2019-033641)
Supplement: Supplementary data [file bmjopen-2019-033641supp002.pdf]

The Pre-BRA (Pre-pectoral Breast Reconstruction Evaluation) Feasibility Study: Protocol
KL Harvey, N Mills, P White, C Holcombe, S Potter
Appendix 2.

CASE REPORT FORM

**Unit Name**	
**REDCap Record ID***	| | | | | | | | |

Record ID = * Unit code+### e.g. NBT001, NBT002 etc.

**Participant email address**	

The Pre-BRA (Pre-pectoral Breast Reconstruction Evaluation) Feasibility Study: Protocol
KL Harvey, N Mills, P White, C Holcombe, S Potter
Appendix 2.

Pre-Op and Operative Data

Pre-BRA

The Pre-BRA (Pre-pectoral Breast Reconstruction Evaluation) Feasibility Study: Protocol
KL Harvey, N Mills, P White, C Holcombe, S Potter
Appendix 2.

Participant Data

Participant withdrawn? Yes ☐ No ☐

(*Only tick 'yes' if the patient no longer wishes to participate in the study)*

Participant questionnaire completion: *(includes BREAST-Q PROMS and Pain Scores)*

Baseline* on paper, follow-ups** online ☐

Everything online ☐

Everything on paper - *PLEASE INFORM CENTRAL STUDY TEAM* ☐

*Baseline defined as: baseline (pre-operative) BREAST-Q PROMS and 24-hours post-op pain score.
**Follow-ups defined as: 3-month and 18-month BREAST-Q PROMS and 1 week, 2 weeks and 3-months pain scores

IF the patient has chosen to complete baseline PROMS on paper and follow-ups online, please offer the participant:

i. a PAPER copy of the Pre-Operative BREAST-Q AND
ii. ask them to record 24-hour post-op pain score in person (/over the telephone if discharged)
(These results need to be transcribed into REDCap by a member of the research team)
iii. All other PROMS will be requested via email direct to the patient

IF the patient has chosen to complete all online PLEASE NOTE:

The patient's record including email address and date of surgery **must** be saved on REDCap on or before the day of surgery in order to generate electronic baseline (pre-operative) BREAST-Q and 24hours post-op pain score requests.

IF the patient has chosen to complete everything on paper, please offer the participant:

i. a PAPER copy of the Pre-Operative BREAST-Q for completion prior to surgery
ii. PAPER copies of the Post-operative BREAST-Q for completion at 3 and 18 months post-operatively (could be sent by post) AND
iii. Ask them to record 24 hour post-op pain score in person (/over the telephone if discharged)
iv. 1 week, 2 weeks and 3 months post-op pain scores can be collected by telephone call from a member of the research team.
(These results need to be transcribed into REDCap by a member of the research team)

Harvey KL, *et al. BMJ Open* 2020; 10:e033641. doi: 10.1136/bmjopen-2019-033641

The Pre-BRA (Pre-pectoral Breast Reconstruction Evaluation) Feasibility Study: Protocol
KL Harvey, N Mills, P White, C Holcombe, S Potter

Pre-BRA Section 1 - Pre-operative data

Co-morbidity data

Patient age (in years) on entering the study …………………. **Years**

Planned* **date of primary mastectomy and reconstruction**
*(*Required to generate automatic electronic BREAST-Q PROMS and post-operative pain score requests via REDCap)*

……../………./….…..(DD/MM/YYYY)

Laterality of planned surgery?

Right breast ☐ Left Breast ☐ Bilateral ☐

Indication for RIGHT mastectomy and planned reconstruction? (leave blank if n/a)

Risk reducing ☐ Malignancy ☐

Indication for LEFT mastectomy and planned reconstruction? (leave blank if n/a)

Risk reducing ☐ Malignancy ☐

Answer ONLY IF indication for surgery is malignancy: (leave blank if n/a)

RIGHT BREAST:

First operation for cancer ☐
Completion mastectomy* ☐
Local recurrence ☐

LEFT BREAST:

First operation for cancer ☐
Completion mastectomy* ☐
Local recurrence ☐

** i.e. Mastectomy performed following unsuccessful breast conservation surgery for example wide local excision (WLE) or therapeutic mammoplasty (TM)*

Medical co-morbidities?

Yes ☐ No ☐

Answer ONLY IF medical comorbidities present: (please tick all relevant)

Diabetes ☐
Asthma/ COPD ☐
Ischaemic heart disease ☐
Connective tissue disease ☐
Current steroid therapy ☐
Anticoagulant therapy (prior to surgery) ☐
Other co-morbidity ☐

If other, please supply details: ……………………………………………………………..

Harvey KL, *et al. BMJ Open* 2020; 10:e033641. doi: 10.1136/bmjopen-2019-033641

The Pre-BRA (Pre-pectoral Breast Reconstruction Evaluation) Feasibility Study: Protocol
KL Harvey, N Mills, P White, C Holcombe, S Potter
Appendix 2.

Prior and neo-adjuvant treatments

Previous radiotherapy to ipsilateral breast or chest wall
(MEETS RELATIVE EXCLUSION CRITERIA)

Yes ☐ No ☐

Neo-adjuvant chemotherapy prior to mastectomy and reconstruction?

Yes ☐ No ☐

Previous surgery to ipsilateral breast?

No ☐ (Please go to Section 2))

Yes ☐

(REDCap™ will open the following options, please tick all relevant and indicate date of surgery:)

Previous axillary surgery ☐ Year.................................

Wide local excision ☐ Year................................

Augmentation ☐ Year.................................

Reduction ☐ Year.................................

Other ☐ Year.................................

Pre-BRA Section 2 - Assessment of suitability for pre-pectoral breast reconstruction

Smoking status (RELATIVE EXCLUSION if current smoker)

Non-smoker ☐

Current smoker ☐

Nicotine replacement/ vaping with nicotine ☐

Recent ex-smoker (i.e. stopped at point of diagnosis) ☐

Grade of ptosis

0 No ptosis ☐

1 Mild - Nipple at level of IMF and above most of lower breast tissue ☐

2 Moderate - Nipple below IMF, but higher than most of the breast tissue ☐

3 Advanced - Nipple below IMF and at level of maximal breast projection ☐

4 Severe - Nipple far below IMF and points towards the floor ☐

Predicted implant vol (cc) ....

The Pre-BRA (Pre-pectoral Breast Reconstruction Evaluation) Feasibility Study: Protocol
KL Harvey, N Mills, P White, C Holcombe, S Potter
Appendix 2.
(>600cc fixed volume **MEETS RELATIVE EXCLUSION CRITERIA***)*

Anticipated adjuvant therapy (RELATIVE EXCLUSION if PMRT is anticipated)

Post-mastectomy radiotherapy?

Not required (risk reduction)	☐	Possible	☐
No, previously received	☐	Probable (positive nodes, large tumour)	☐
Unlikely (e.g. DCIS only)	☐		

Body Mass Index

Patient height (cm) ……………………………. Patient weight (kg) …………………………….

BMI (REDCap™ calculates) ……………………. **(RELATIVE EXCLUSION if >30)**

Manual BMI entry *(only answer if height/ weight values unknown)* ……………………….

Suitability for pre-pectoral reconstruction

RELATIVE CONTRA-INDICATIONS:

- Previous breast/ chest wall radiotherapy or mantle radiotherapy
- Anticipated post-mastectomy radiotherapy
- BMI >30
- Current smoker (or nicotine dependent)
- Implant volume >600cc

What is your clinical impression of this patient's risk of experiencing a post-operative complication?

They are at low risk ☐ (Defined as no relative contraindications)

They are at moderate risk ☐ (Defined as 1 relative contra-indication)

They are at high risk ☐ (Defined as >1 relative contraindication)

They are high risk for another reason ☐

Please supply details if you have specified another reason

…………………………………………………………………………………………………

…………………………………………………………………………………………………

…………………………………………………………………………………………………

Are any additional surgical procedures planned at a later date? *(please select all that apply)*

Harvey KL, *et al. BMJ Open* 2020; 10:e033641. doi: 10.1136/bmjopen-2019-033641

The Pre-BRA (Pre-pectoral Breast Reconstruction Evaluation) Feasibility Study: Protocol
KL Harvey, N Mills, P White, C Holcombe, S Potter
Appendix 2.

Lipo-modelling ☐

Nipple reconstruction ☐

Contralateral symmetrising Reduction/ Mastopexy/ Augmentation ☐

No further procedures planned ☐

Pre-BRA Section 3 - Modifications to Usual Patient Selection Criteria

Have you modified your usual patient selection criteria in order to offer this patient a pre-pectoral implant reconstruction?

Yes ☐ No ☐

If you have answered "yes", please explain in what way: ....

....

....

....

....

Note: The study team are very interested in speaking to surgeons to explore any modifications made to their usual patient selection criteria.

Does the operating surgeon consent to be contacted for a brief telephone interview?

Yes ☐ No ☐

Please supply the email address of the operating surgeon if yes:

....

2.12 FINAL STUDY ENTRY CHECKLIST

**2.8.1 Meets inclusion/exclusion criteria for the study**	**Yes/No**
**2.8.2 Written consent to participate in the study**	**Yes/No**
**2.8.3 Pre-op PROMs (Breast-Q) completed**	**Yes/No**

7

The Pre-BRA (Pre-pectoral Breast Reconstruction Evaluation) Feasibility Study: Protocol
KL Harvey, N Mills, P White, C Holcombe, S Potter
Appendix 2.

Pre-BRA Section 4 - Operative data

Actual* **date of primary mastectomy and reconstruction**

........./………../..……..(DD/MM/YYYY)

**If the planned (see section 1.) and actual dates of surgery differ, please contact central study team to confirm which date is correct. This is important for generating electronic BREAST-Q PROMS and post-operative pain score requests.*

ASA grade

1	Normal healthy individual	☐
2	Mild systemic disease that does not limit activities	☐
3	Severe systemic disease that limits activities but is not incapacitating	☐
4	Incapacitating systematic disease which is constantly life-threatening	☐

Name of operating Consultant surgeon

...

Procedure performed in theatre with laminar flow system in situ?

Yes ☐ No ☐

Type of skin prep used at time of surgery

Iodine (Betadine) (brown)	☐	Chlorhexidine (pink)	☐
2% Chlorprep	☐	Other (please specify)	☐

...

Planned antibiotic use

Prophylactic only (<24 hours)	☐	1-5 days	☐
Extended course (5+ days)	☐	Until drains removed	☐
Other (please specify)	☐		

……………………………………………………………………………………………………..

Antibiotics used, please include dose/ frequency (free text)

……………………………………………………………………………………………………..

Duration of procedure (knife to skin to dressings on) ....(Minutes)

8

The Pre-BRA (Pre-pectoral Breast Reconstruction Evaluation) Feasibility Study: Protocol
KL Harvey, N Mills, P White, C Holcombe, S Potter
Appendix 2.

Did you adhere to the steps advised in The Theatre Implant Checklist or 'TIC List'? (REDCap™ displays "TIC" List) Please tick all steps which were completed in this case:

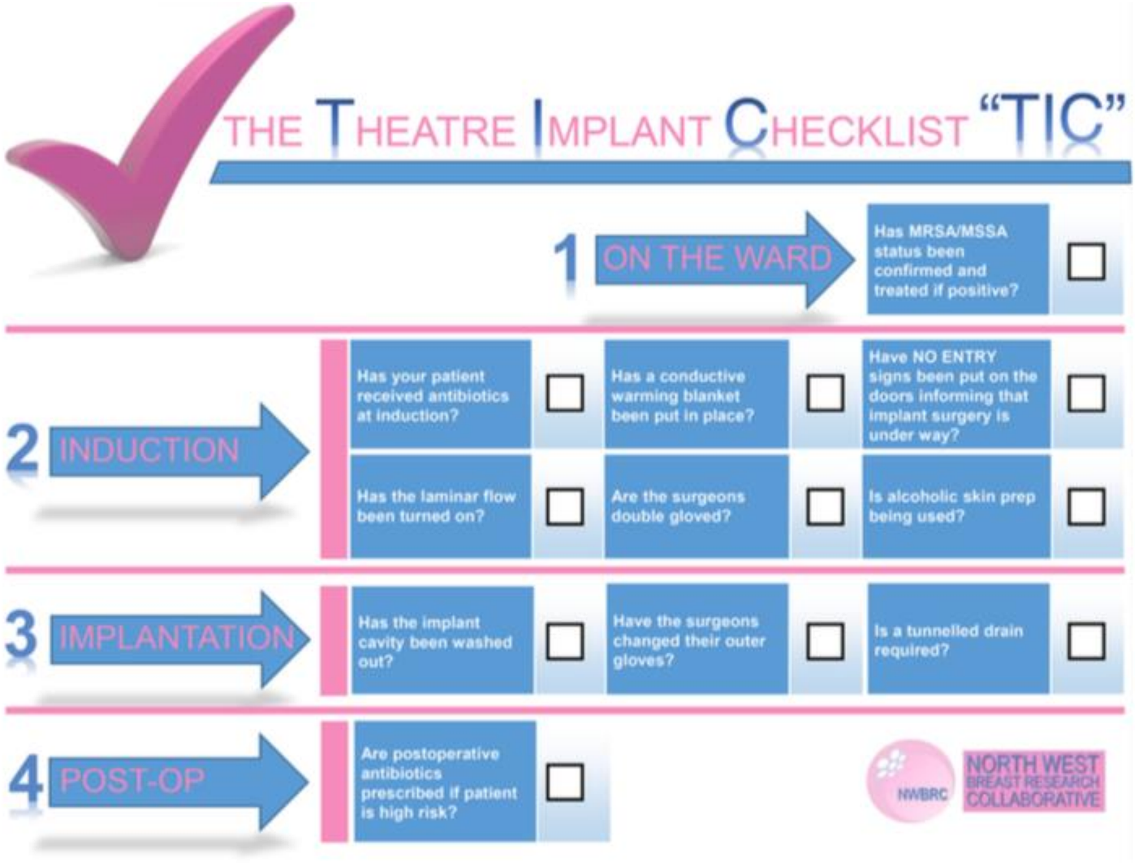

(Image reproduced from Barr, S.P., et al., *Infection prevention in breast implant surgery - A review of the surgical evidence, guidelines and a checklist.* European Journal of Surgical Oncology, 2016. **42**: p. 591-603.)

	**Yes /**	**No**
Patient received antibiotics at induction?	☐	☐
Conductive warming blanket been put in place?	☐	☐
NO ENTRY signs been put on the doors informing that implant surgery is under way?	☐	☐
Are the surgeons double gloved?	☐	☐
Is alcoholic skin prep being used?	☐	☐
Implant cavity washed out?	☐	☐
(Before implantation) Have the surgeons changed their outer gloves?	☐	☐
Is a tunnelled drain required?	☐	☐
Are post-operative antibiotics prescribed if patient is high risk?	☐	☐

9

The Pre-BRA (Pre-pectoral Breast Reconstruction Evaluation) Feasibility Study: Protocol
KL Harvey, N Mills, P White, C Holcombe, S Potter
Appendix 2.

Regarding length of stay, is the patient planned for:

Day case (i.e. home the same day as surgery) ☐

Overnight stay (i.e. home the day after surgery) ☐

Inpatient stay (>1 night) ☐

Was surgery performed on: (please tick all that apply)

Right breast ☐ Left breast ☐

**Right breast procedure:**		**Left breast procedure:**	
Implant reconstruction	☐	Implant reconstruction	☐
Other reconstruction	☐	Other reconstruction	☐
Augmentation	☐	Augmentation	☐
Reduction	☐	Reduction	☐
Mastopexy	☐	Mastopexy	☐
Other	☐	Other	☐

If other selected, please give details: ………………………………………………………………..

………

Harvey KL, *et al. BMJ Open* 2020; 10:e033641. doi: 10.1136/bmjopen-2019-033641

The Pre-BRA (Pre-pectoral Breast Reconstruction Evaluation) Feasibility Study: Protocol
KL Harvey, N Mills, P White, C Holcombe, S Potter
Appendix 2.

Pre-BRA Section 5a. RIGHT BREAST DATA

(Please only answer Section 5a. if you have performed a PRE-PECTORAL IMPLANT RECONSTRUCTION on the patient's RIGHT BREAST)

Pre-operative Planning: RIGHT BREAST

Right breast – What do you plan to do upfront i.e. prior to starting the operation?

One stage reconstruction ☐ Two-stage reconstruction ☐

Intra-operative Detail

Right breast - Grade of primary operating surgeon

Consultant ☐ SAS grade ☐

Senior trainee (OPF/ST8+) ☐ ST6/7 ☐

ST5 or below ☐ Other ☐

If other selected, please give details: …………………………………………………………………..

Right breast - Number of pre-pectoral implant-based reconstructions using this method/ technique the primary surgeon has **previously performed:**

In total (i.e. both supervised and unsupervised):

Less than 5 ☐ 11-25 ☐

5-10 ☐ More than 25 ☐

Supervised:

Less than 5 ☐ 11-25 ☐

5-10 ☐ More than 25 ☐

Right breast - Type of mastectomy

Skin sparing mastectomy ☐ Skin and nipple preserving ☐

Reduction (Wise) pattern ☐ Other ☐

If other selected, please give details: …………………………………………………………………..

………

11

The Pre-BRA (Pre-pectoral Breast Reconstruction Evaluation) Feasibility Study: Protocol
KL Harvey, N Mills, P White, C Holcombe, S Potter
Appendix 2.

Right breast - Location of incision

Peri-areolar (nipple sparing)	☐	Lateral	☐
Inframammary	☐	Elliptical removing NAC	☐
Wise pattern	☐	Other	☐

If other selected, please give details: ……………………………………………………………..

……………………………………………………………………………………………………

Right breast – Was infiltration in the mastectomy plane i.e. hydro-dissection used?

No	☐	With saline only	☐
With local anaesthetic +/- saline	☐	LA and adrenaline +/- saline	☐

Right breast - Main instrument used for dissection of mastectomy skin flaps

Blade	☐	Scissors	☐
Handheld monopolar diathermy	☐	Diathermy scissors	☐
Combination of methods	☐	Other	☐

If other selected, please give details: ……………………………………………………………..

……………………………………………………………………………………………………

Right breast - Mastectomy dry weight (gms) ………………………………………

<u>Feasibility of Pre-Pectoral Reconstruction</u>

Right breast - Surgeon's assessment of skin flap quality

Good – healthy, well-perfused, no concerns noted at time of surgery	☐
Average – intermediate – no obvious concerns at time of surgery	☐
Poor – thin flaps, questionable vascularity; concerns at time of surgery	☐

Right breast - Did you complete the reconstruction as planned? (i.e. as per your answer to the first question of Section 5?)

Yes	☐	No	☐

12

The Pre-BRA (Pre-pectoral Breast Reconstruction Evaluation) Feasibility Study: Protocol
KL Harvey, N Mills, P White, C Holcombe, S Potter
Appendix 2.

Right breast - What operation did you actually perform?

i. Pre-pectoral approach considered safe and performed with fixed volume implant ☐

ii. Pre-pectoral reconstruction performed with variable volume implant (e.g. Becker) ☐

iii. Pre-pectoral reconstruction performed with a tissue expander ☐

iv. Pre-pectoral reconstruction abandoned ☐

If you selected answer iv. please also answer the next two questions ("Why?" and "What did you do instead?")

If you answered i, ii or iii please go straight to the third question down ("Right breast - type of implant coverage")

Why was pre-pectoral approach abandoned?

……………………………………………………………………………………

……………………………………………………………………………………..

……………………………………………………………………………………

What procedure was performed instead

No reconstruction ☐

Sub-pectoral reconstruction with tissue expander (no mesh) ☐

Sub-pectoral reconstruction with biological mesh ☐

Sub-pectoral reconstruction with synthetic mesh ☐

Other ☐

If other selected, please give details: **……………………………………………………………..**

……………………………………………………………………………………………………

Right breast - type of implant coverage

No mesh or ADM coverage (subcutaneous implant only) ☐

Biological mesh (e.g. ADM) ☐

Synthetic mesh (e.g. TiLOOP) ☐

Complete dermal sling ☐

Dermal sling and biological mesh in combination ☐

13

The Pre-BRA (Pre-pectoral Breast Reconstruction Evaluation) Feasibility Study: Protocol
KL Harvey, N Mills, P White, C Holcombe, S Potter
Appendix 2.

Dermal sling and synthetic mesh in combination ☐

Hybrid implant coverage (e.g. biological mesh at top/synthetic mesh at bottom) ☐

Other ☐

If 'Hybrid implant coverage' or 'other' selected, please supply details: ………………………….

……

Right breast - type of implant coverage, if you selected 'Biological mesh' please select which type:

BRAXON	☐	Artia	☐
Surgimend	☐	Fortiva	☐
Strattice	☐	Cellis	☐
Veritas	☐	Exaflex Pocket	☐
MESO	☐	Native	☐
Tutomesh	☐		

Other biological ADM/ mesh (please state) ……………………………………………..☐

……

Right breast - type of implant coverage, if you selected 'synthetic mesh' please select which type:

TiLOOP	☐	Galaflex	☐
TiGR	☐		

Other (please state) ……………………………………………………………………….☐

Right breast - Breast prosthesis details

Fixed volume implant ☐
Size (ccs)……………………………………………

OR

Temporary tissue expander ☐

Vol of saline inserted (mls)…………………………

Size when fully expanded (ccs)……………………..

OR

Combined implant (Beckers) ☐

14

The Pre-BRA (Pre-pectoral Breast Reconstruction Evaluation) Feasibility Study: Protocol
KL Harvey, N Mills, P White, C Holcombe, S Potter
Appendix 2.

Silicone component (g).......................... Vol of saline inserted (mls)…........................

Size when fully expanded (ccs)........................

Right breast – shape of implant

Round ☐ Anatomical ☐

Right breast – texture of implant

Smooth ☐ Textured/ Microtextured ☐

Polyurethane ☐

Right breast – Was the prosthesis bathed/washed prior to insertion?

Yes ☐ No ☐

If 'Yes' selected which wash was used?

Saline ☐ Antiseptic ☐

Antibiotics ☐ Other (please give details) ☐

....

Right breast – Was the cavity washed or irrigated prior to insert of implant?

Yes ☐ No ☐

If 'Yes' selected what irrigation fluid was used?

Saline ☐ Antiseptic ☐

Antibiotics ☐ Other (please give details) ☐

....

Surgeon glove change prior to handling the implant?

Yes ☐ No ☐

Right Breast - Were the skin edges freshened/excised prior to wound closure?

Yes ☐ No ☐

Right breast – Type of axillary surgery

None ☐

The Pre-BRA (Pre-pectoral Breast Reconstruction Evaluation) Feasibility Study: Protocol
KL Harvey, N Mills, P White, C Holcombe, S Potter
Appendix 2.

SLNB/targeted sample post NAC ☐

Axillary sample (failed SLNB) ☐

Axillary clearance ☐

SLNB + ANC (e.g. positive OSNA) ☐

Right breast – Number of (tunnelled) drains used (per side if bilateral case)

None ☐ One ☐ Two ☐

Right breast – intra-operative analgesia used

Local/regional block (e.g. pectoral or serratus block) ☐

Infiltration of LA as part of hydro dissection/tumescence ☐

Infiltration of LA into wounds prior to starting case ☐

Infiltration of LA into wounds at the end of the case ☐

LA inserted down drains ☐

Intra-pocket LA catheter (continuous infusion of LA post-op) ☐

LA not used ☐

Other ☐

Right breast dressings used (please select as many as apply)

Occlusive adhesive dressing (e.g. Opsite) ☐

Skin glue (e.g. Liquiband) ☐

Skin closure system - skin glue and tape (e.g. Dermabond Prineo dressing) ☐

Negative pressure dressing e.g. PICO ☐

Other ☐

The Pre-BRA (Pre-pectoral Breast Reconstruction Evaluation) Feasibility Study: Protocol
KL Harvey, N Mills, P White, C Holcombe, S Potter
Appendix 2.

Pre-BRA Section 5b. LEFT BREAST DATA

(Please only answer Section 5b. if you have performed a PRE-PECTORAL IMPLANT RECONSTRUCTION on the patient's LEFT BREAST)

Pre-operative Planning: LEFT BREAST

Left breast – What do you plan to do upfront i.e. prior to starting the operation?

One stage reconstruction ☐ Two-stage reconstruction ☐

Intra-operative Detail

Left breast - Grade of primary operating surgeon

Consultant	☐	SAS grade	☐
Senior trainee (OPF/ST8+)	☐	ST6/7	☐
ST5 or below	☐	Other	☐

If other selected, please give details: ………………………………………………………………..

Left breast - Number of pre-pectoral implant-based reconstructions using this method the primary surgeon has **previously performed:**

In total (i.e. both supervised and unsupervised):

Less than 5	☐	11-25	☐
5-10	☐	>25	☐

Supervised:

Less than 5	☐	11-25	☐
5-10	☐	>25	☐

Left breast - Type of mastectomy

Skin sparing mastectomy	☐	Skin and nipple preserving	☐
Reduction (wise) pattern	☐	Other	☐

If other selected, please give details: ………………………………………………………………..

……

Harvey KL, *et al. BMJ Open* 2020; 10:e033641. doi: 10.1136/bmjopen-2019-033641

The Pre-BRA (Pre-pectoral Breast Reconstruction Evaluation) Feasibility Study: Protocol
KL Harvey, N Mills, P White, C Holcombe, S Potter
Appendix 2.

Left breast - Location of incision

Peri-areolar (nipple sparing)	☐	Lateral	☐
Inframammary	☐	Elliptical removing NAC	☐
Wise pattern	☐	Other	☐

If other selected, please give details: ……………………………………………………………..

………

Left breast – Was infiltration in the mastectomy plane/hydro-dissection used?

No	☐	With saline only	☐
With local anaesthetic +/- saline	☐	LA and adrenaline +/- saline	☐

Left breast - Main instrument used for dissection of mastectomy skin flaps

Blade	☐	Scissors	☐
Handheld monopolar diathermy	☐	Diathermy scissors	☐
Combination of methods	☐	Other	☐

If other selected, please give details: ……………………………………………………………..

………

Left breast - Mastectomy dry weight (gms) ………………………………………

<u>Feasibility of Pre-Pectoral Reconstruction</u>

Left breast - Surgeon's assessment of skin flap quality

Good – healthy, well-perfused, no concerns noted at time of surgery	☐
Average – intermediate – no obvious concerns at time of surgery	☐
Poor – thin flaps, questionable vascularity; concerns at time of surgery	☐

Left breast - Did you complete the reconstruction as planned? (i.e. as per your answer to the first question of Section 3b?)

Yes	☐	No	☐

Left breast – if 'no', what operation did you actually perform?

Harvey KL, *et al. BMJ Open* 2020; 10:e033641. doi: 10.1136/bmjopen-2019-033641

The Pre-BRA (Pre-pectoral Breast Reconstruction Evaluation) Feasibility Study: Protocol
KL Harvey, N Mills, P White, C Holcombe, S Potter
Appendix 2.

i. Pre-pectoral approach considered safe and performed with fixed volume implant ☐

ii. Pre-pectoral reconstruction performed with variable volume implant (e.g. Becker) ☐

iii. Pre-pectoral reconstruction performed with a tissue expander ☐

iv. Pre-pectoral reconstruction abandoned ☐

If you selected answer iv. please also answer the next two questions ("Why?" and "What did you do instead")

If you answered i, ii or iii please go straight to the third question down ("Left breast - type of implant coverage")

Why was pre-pectoral approach abandoned?

………………………………………………………………………………………

……………………………………………………………………………………..

………………………………………………………………………………………

What procedure was performed instead

No reconstruction ☐

Sub-pectoral reconstruction with tissue expander (no mesh) ☐

Sub-pectoral reconstruction with biological mesh ☐

Sub-pectoral reconstruction with synthetic mesh ☐

Other ☐

Left breast - type of implant coverage

No mesh or ADM coverage (subcutaneous implant only) ☐

Biological mesh (e.g. ADM) ☐

Synthetic mesh (e.g. TiLOOP) ☐

Complete dermal sling ☐

Dermal sling and biological mesh in combination ☐

Dermal sling and synthetic mesh in combination ☐

The Pre-BRA (Pre-pectoral Breast Reconstruction Evaluation) Feasibility Study: Protocol
KL Harvey, N Mills, P White, C Holcombe, S Potter
Appendix 2.

Hybrid implant coverage (e.g. biological mesh at top/synthetic mesh at bottom) ☐

Other ☐

If 'Hybrid implant coverage' or 'other' selected, please supply details: ………………………….

………

Left breast - type of implant coverage, if you selected 'Biological mesh' please select which type:

BRAXON	☐	Artia	☐
Surgimend	☐	Fortiva	☐
Strattice	☐	Cellis	☐
Veritas	☐	Exaflex Pocket	☐
MESO	☐	Native	☐
Tutomesh	☐		

Other biological ADM/ mesh (please state) ……………………………………………… ☐

………

Left breast - type of implant coverage, if you selected 'synthetic mesh' please select which type:

TiLOOP	☐	Galaflex	☐
TiGR	☐		

Other (please state) ……………………………………………………………………… ☐

Left breast - Breast prosthesis details

Fixed volume implant ☐
Size (ccs)………………………………………………

OR

Temporary tissue expander ☐

Vol of saline inserted (mls)…………………………
Size when fully expanded (ccs)………………………

OR

Combined implant (Beckers) ☐

Silicone component (g)……………………… Vol of saline inserted (mls)………………………

20

The Pre-BRA (Pre-pectoral Breast Reconstruction Evaluation) Feasibility Study: Protocol
KL Harvey, N Mills, P White, C Holcombe, S Potter
Appendix 2.

Size when fully expanded (ccs).........................

Left breast – shape of implant

Round ☐ Anatomical ☐

Left breast – texture of implant

Smooth ☐ Textured/ Microtextured ☐

Polyurethane ☐ I don't know ☐

Left breast – Was the prosthesis bathed/washed prior to insertion?

Yes ☐ No ☐

If 'Yes' selected which wash was used?

Saline ☐ Antiseptic ☐

Antibiotics ☐ Other (please give details) ☐

....

Left breast – Was the cavity washed or irrigated prior to insert of implant?

Yes ☐ No ☐

If 'Yes' selected what irrigation fluid was used?

Saline ☐ Antiseptic ☐

Antibiotics ☐ Other (please give details) ☐

....

Left Breast - Surgeon glove change prior to handling the implant?

Yes ☐ No ☐

Left Breast - Were the skin edges freshened/excised prior to wound closure?

Yes ☐ No ☐

Left breast – Type of axillary surgery

None ☐ (Please go to question 3.36)

SLNB/targeted sample post NAC ☐ Axillary sample (failed SLNB) ☐

Axillary clearance ☐ SLNB + ANC (e.g. positive OSNA) ☐

The Pre-BRA (Pre-pectoral Breast Reconstruction Evaluation) Feasibility Study: Protocol
KL Harvey, N Mills, P White, C Holcombe, S Potter
Appendix 2.

Left breast – Number of (tunnelled) drains used

None ☐ One ☐ Two ☐

Left breast – intra-operative analgesia used

Local/regional block (e.g. pectoral or serratus block) ☐

Infiltration of LA as part of hydro dissection/tumescence ☐

Infiltration of LA into wounds prior to starting case ☐

Infiltration of LA into wounds at the end of the case ☐

LA inserted down drains ☐

LA not used ☐
Intra-pocket LA catheter (continuous infusion of LA post-op)

Other ☐

Left breast dressings used (select as many as apply)

Occlusive adhesive dressing (e.g. Opsite) ☐

Skin glue (e.g. Liquiband) ☐

Skin closure system - skin glue and tape (e.g. Dermabond Prineo dressing) ☐

Negative pressure dressing e.g. PICO ☐
Other

The Pre-BRA (Pre-pectoral Breast Reconstruction Evaluation) Feasibility Study: Protocol
KL Harvey, N Mills, P White, C Holcombe, S Potter
Appendix 2.

Pre-BRA Section 6 – Modifications to Usual Operative Technique

Were any modifications made to the operative technique used in this case?

Yes ☐ No ☐

If you have answered "**yes**", please provide details about:

i. What was modified and how? (e.g. mesh sutured in a different way) ………………………...

……………………………………………………………………………………………………..

……………………………………………………………………………………………………..

……………………………………………………………………………………………………..

ii. Why was the modification made? (e.g. difficulty accessing mesh) …………………………...

……………………………………………………………………………………………………..

……………………………………………………………………………………………………..

……………………………………………………………………………………………………..

<u>Please Note</u>: The study team are very interested in speaking to surgeons to explore any modifications made to the procedure.

Does the operating surgeon consent to be contacted for a brief telephone interview?

Yes ☐ No ☐

Please supply the email address of the operating surgeon if yes:

…………………………………………………………………………………………

Pre-BRA Section 7 – Peri Discharge

<u>Length of Stay</u>

Was the patient managed as a day case?
(defined as: discharged on the same day as surgery)

Yes ☐ No ☐

The Pre-BRA (Pre-pectoral Breast Reconstruction Evaluation) Feasibility Study: Protocol
KL Harvey, N Mills, P White, C Holcombe, S Potter
Appendix 2.

Date of discharge (DD/MM/YYYY) ………………/……………/……………………..

Length of post-operative stay…………..………………. (no. of post-operative days in hospital, calculated from date of surgery (= Day 0) to date of discharge)

(REDCap will auto-populate using the answers from date of surgery and the answer above)

END OF PRE-OPERATIVE AND OPERATIVE DATA SECTION

Harvey KL, *et al. BMJ Open* 2020; 10:e033641. doi: 10.1136/bmjopen-2019-033641

The Pre-BRA (Pre-pectoral Breast Reconstruction Evaluation) Feasibility Study: Protocol
KL Harvey, N Mills, P White, C Holcombe, S Potter
Appendix 2.

Pain Scores

Pre-BRA

<u>Please NOTE:</u>

If the participant does not wish to complete pains core data entry online via email link they must be collected in person/ via telephone call and transcribed into REDCap's "Pain scores" section by the research team

Pain score at 24 hours' post-reconstruction **(0 - 10)*** ………………

Pain score at 1-week post-reconstruction: **(0 - 10)** ………………

Pain score at 2 weeks' post-reconstruction: **(0 - 10)** ………………

Pain score at 3 months' post-reconstruction: **(0 - 10)** ………………

* 0-10 Numeric pain rating scale, where:
0= No pain, 5= Moderate pain, 10= worst possible pain

The Pre-BRA (Pre-pectoral Breast Reconstruction Evaluation) Feasibility Study: Protocol
KL Harvey, N Mills, P White, C Holcombe, S Potter
Appendix 2.

3 Month Data

Pre-BRA

26

The Pre-BRA (Pre-pectoral Breast Reconstruction Evaluation) Feasibility Study: Protocol
KL Harvey, N Mills, P White, C Holcombe, S Potter
Appendix 2.

Pre-BRA Section 8 – Oncological outcome and adjuvant treatment data

Not applicable – risk reducing surgery ☐ (No further questions in Section 8)

RIGHT BREAST
(Only answer the following questions if you operated for malignancy on the patient's **right breast)**

Right breast – if neoadjuvant chemotherapy – complete pathological response?

Yes ☐ No ☐

Right breast - Invasive status

Invasive ☐ DCIS ☐

Right breast – Focality

Unifocal ☐ Multifocal ☐

Right breast - Grade of DCIS or invasive carcinoma

(from core biopsy if complete pathological response)

1 – Low grade (DCIS) or well-differentiated (invasive) ☐

2 – Intermediate grade (DCIS) or moderately differentiated (invasive) ☐

3 – High grade (DCIS) or poorly differentiated (invasive) ☐

Right breast - Lymph node involvement

Total number of involved nodes (macromets only) ....

Total number of lymph nodes in pathology specimen..

Right breast - Invasive lesion size (mm)

(largest lesion if multifocal) ....

Right side - Planned delayed axillary clearance

Yes ☐ No ☐

LEFT BREAST
(Only answer the following questions if you operated for malignancy on the patient's **left breast)**

Left breast – if neoadjuvant chemotherapy – complete pathological response?

Yes ☐ No ☐

Left breast - Invasive status

27

The Pre-BRA (Pre-pectoral Breast Reconstruction Evaluation) Feasibility Study: Protocol
KL Harvey, N Mills, P White, C Holcombe, S Potter
Appendix 2.

Invasive ☐ DCIS ☐

Left breast – Focality

Unifocal ☐ Multifocal ☐

Left breast - Grade of DCIS or invasive carcinoma
(from core biopsy if complete pathological response)

1 – Low grade (DCIS) or well-differentiated (invasive) ☐

2 – Intermediate grade (DCIS) or moderately differentiated (invasive) ☐

3 – High grade (DCIS) or poorly differentiated (invasive) ☐

Left breast - Lymph node involvement

6.13.1 Total number of involved nodes (macromets only) ....

6.13.2 Total number of lymph nodes in pathology specimen..

Left breast - Invasive lesion size (mm)

(largest lesion if multifocal) ....

Left side - Planned delayed axillary clearance

Yes ☐ No ☐

PLANNED ADJUVANT THERAPY
(Answer the following questions for **all cases of malignancy**)

Chemotherapy

Yes ☐ No ☐ Already received ☐

Radiotherapy

Right side

to chest wall	Yes	☐	No	☐
to axilla	Yes	☐	No	☐
to SCF*	Yes	☐	No	☐
to IMC**	Yes	☐	No	☐

Left side

to chest wall	Yes	☐	No	☐
to axilla	Yes	☐	No	☐
to SCF*	Yes	☐	No	☐

Harvey KL, *et al. BMJ Open* 2020; 10:e033641. doi: 10.1136/bmjopen-2019-033641

The Pre-BRA (Pre-pectoral Breast Reconstruction Evaluation) Feasibility Study: Protocol
KL Harvey, N Mills, P White, C Holcombe, S Potter
Appendix 2.

to IMC** Yes ☐ No ☐

(*Supraclavicular fossa, ** internal mammary chain)

Endocrine therapy Yes ☐ No ☐

Anti-Her2 therapy Yes ☐ No ☐

Date of FIRST adjuvant treatment
(i.e. date of first dose of chemotherapy, if applicable or 1st fraction of radiotherapy)

(DD/MM/YYYY)……………………………………………….

Pre-BRA Section 9– Complication(s) at 3 Months

Post-operative complication experienced <u>IN THE FIRST 3 MONTHS</u>?

Yes ☐ No ☐

Readmission to hospital <u>IN THE FIRST 3 MONTHS</u>
Was the patient readmitted to hospital to manage a complication in the first 3 months post-operatively?

Yes ☐ No ☐ Date (DD/MM/YYYY) ……………………

If 'Yes' selected, please give reason for readmission:

………………………………………………………………………………………

……………………………………………………………………………………..

………………………………………………………………………………………

Re-operation for complications <u>IN THE FIRST 3 MONTHS</u>
Did the patient undergo an unplanned re-operation (i.e. following a complication) in the first 3 months post-operatively?

Yes ☐ No ☐ Date (DD/MM/YYYY) ……………………

If 'Yes' selected, please give reason for re-operation:

………………………………………………………………………………………

……………………………………………………………………………………..

………………………………………………………………………………………

The Pre-BRA (Pre-pectoral Breast Reconstruction Evaluation) Feasibility Study: Protocol
KL Harvey, N Mills, P White, C Holcombe, S Potter
Appendix 2.

Breast Complication Details:

Please indicate if a complication was experienced, indicate which side was affected and the date it was first noticed

	Right	Left	Bilateral	Date (DD/MM/YYYY)
**Seroma?**				
No aspiration required	☐	☐	☐	....
Requiring aspiration 1-2 times	☐	☐	☐	....
Requiring aspiration 3 or more times	☐	☐	☐	....
**Haematoma?**				
Minor - Managed conservatively	☐	☐	☐	....
Major 1 - Requiring aspiration in clinic (+/-USS, no GA)	☐	☐	☐	....
Major 2 - Requiring evacuation in theatre	☐	☐	☐	....
**Wound infection?**				
Minor - Requiring oral antibiotics	☐	☐	☐	....
Major 1 - Requiring admission for IV antibiotics	☐	☐	☐	....
Major 2 - Requiring surgical drainage or debridement	☐	☐	☐	....
**Mastectomy skin flap necrosis?**				
Minor – Managed conservatively	☐	☐	☐	....
Major 1 – Requiring surgical debridement in clinic (no GA)	☐	☐	☐	....
Major 2 – Requiring surgical debridement in theatre (GA)	☐	☐	☐	....
**Nipple necrosis?**				
Minor – Managed conservatively	☐	☐	☐	....
Major 1 – Requiring surgical debridement	☐	☐	☐	....
Major 2 – Total NAC loss	☐	☐	☐	....

Harvey KL, *et al. BMJ Open* 2020; 10:e033641. doi: 10.1136/bmjopen-2019-033641

The Pre-BRA (Pre-pectoral Breast Reconstruction Evaluation) Feasibility Study: Protocol
KL Harvey, N Mills, P White, C Holcombe, S Potter
Appendix 2.

	Right	Left	Bilateral	Date (DD/MM/YYYY)
**Wound dehiscence?**				
Minor – Managed conservatively	☐	☐	☐	…………………………
Major – Requiring return to theatre for re-suturing	☐	☐	☐	…………………………

Implant loss?

Total implant loss
i.e. any unplanned removal of the implant or expander for infection, **without replacement** of the prosthesis ☐ ☐ ☐ …………………………

OR ***Implant salvage*** i.e.

i. Implant removed, washed and replaced with fixed volume/ final implant ☐ ☐ ☐ …………………………

OR ii. Implant removed, washed and replaced with a tissue expander ☐ ☐ ☐ …………………………

(If "***Implant salvage***" selected, additional question:
Was implant salvage a success? (i.e. implant still in place at 3 months)

Yes ☐ No ☐

Mechanical implant problem?
(e.g. deflation) ☐ ☐ ☐ …………………………

Other complication? ☐ ☐ ☐ …………………………

Details…………………………………………………………………………………

Pre-BRA Section 10 – Learning from Post-Operative Problems

Would the operating surgeon consider changing or modifying their **surgical technique** as a result of this complication? Yes ☐ No ☐

Please supply as much detail as possible: …………………………………………………………
……
……

31

The Pre-BRA (Pre-pectoral Breast Reconstruction Evaluation) Feasibility Study: Protocol
KL Harvey, N Mills, P White, C Holcombe, S Potter
Appendix 2.
……………………………………………………………………………………………………

Would the operating surgeon consider changing or modifying their **patient selection criteria** as a result of this complication? Yes ☐ No ☐

Please supply as much detail as possible: …………………………………………………………

……………………………………………………………………………………………………

……………………………………………………………………………………………………

……………………………………………………………………………………………………

Please note: The study team are **very interested** in speaking to surgeons who report learning from post-operative problems.
Does the operating surgeon agree to be contacted for a brief telephone interview?

Yes ☐ No ☐

Please supply the email address of the operating surgeon if yes:

……………………………………………………………………………………

Pre-BRA Section 11 – Post-Operative Patient Reported Outcome Measures (PROMS)

7.1 3-month Breast-Q completed? Yes ☐ No ☐

7.2 18-month Breast-Q completed? Yes ☐ No ☐

END OF QUESTIONS

THANK YOU FROM THE PRE-BRA TEAM!

Pre-BRA

Harvey KL, *et al. BMJ Open* 2020; 10:e033641. doi: 10.1136/bmjopen-2019-033641
