# Supplementary material for: The Pre-BRA (pre-pectoral Breast Reconstruction EvAluation) feasibility study: protocol for a mixed-methods IDEAL 2a/2b prospective cohort study to determine the safety and effectiveness of prepectoral implant-based breast reconstruction
Source: BMJ Open. 2020 Jan 26;10(1):e033641. doi: 10.1136/bmjopen-2019-033641 (PMC7044855; doi:10.1136/bmjopen-2019-033641)
Supplement: Supplementary data [file bmjopen-2019-033641supp003.pdf]

The Pre-BRA (Pre-pectoral Breast Reconstruction Evaluation) Feasibility Study: Protocol
KL Harvey, N Mills, P White, C Holcombe, S Potter
Appendix 3

Appendix 3

Definitions of Complications

Implant loss - any unplanned removal of the implant without replacement of the prosthesis (implant or expander) for infection, wound problems or other indication within the first 3 months following surgery.

Implant salvage – return to theatre for debridement of wound/ re-suturing /drainage of infection/ washout/ or other indication in which the implant is removed and immediately replaced either with the same device or a tissue expander with primary closure of the wound.

Unsuccessful implant salvage (considered equivalent to implant loss) - the implant is no longer in situ 3 months after the salvage procedure was performed.

Infection - A hot, red swollen breast associated with one of the following; a temperature, pus at the wound site, a raised white cell count and/or; a positive wound swab within the first 3 months following surgery. This will be further classified as:

- Minor – requiring oral antibiotics only;
- Major 1 – requiring admission for IV antibiotics and/or debridement;
- Major 2 – requiring surgical drainage/debridement

Red breast syndrome – Persistent non-infective breast erythema

Readmission to hospital – any re-admission to hospital in the 3 months following surgery directly related to the procedure (e.g. with infection requiring antibiotics).

The Pre-BRA (Pre-pectoral Breast Reconstruction Evaluation) Feasibility Study: Protocol
KL Harvey, N Mills, P White, C Holcombe, S Potter
Appendix 3

Return to theatre – Return to the operating theatre at any time during the first 3 months following surgery to deal with any complication of the reconstruction. This will not include any secondary oncological procedures such as axillary clearance or planned procedures including exchange of expander for a fixed volume implant or lipo-modelling.

Seroma - A symptomatic collection of fluid around the reconstructed breast following surgery requiring aspiration. The total number of aspirations will be collected at 3 months.

Haematoma - A collection of blood in the reconstructed breast

- Minor – managed conservatively or by aspiration in clinic;
- Major – requiring surgical evacuation

Mastectomy skin flap necrosis - Any area of skin loss on the reconstructed breast

- Minor – managed conservatively with dressings;
- Major 1 – requiring debridement (in clinic or theatre) under local anaesthetic (LA);
- Major 2 – requiring surgical debridement under general anaesthesia (GA)

Nipple necrosis – Any area of necrosis of the nipple areolar complex (NAC) (if nipple preserving mastectomy)

- Minor – managed conservatively with dressings;
- Major 1 – requiring surgical debridement under LA in clinic or theatre;
- Major 2 – requiring surgical debridement under GA in theatre

Wound dehiscence – separation of the skin edges at the wound site.

- Minor – treated conservatively;

The Pre-BRA (Pre-pectoral Breast Reconstruction Evaluation) Feasibility Study: Protocol
KL Harvey, N Mills, P White, C Holcombe, S Potter
Appendix 3

- Major – requiring return to theatre for re-suturing under GA

Displaced implant requiring repositioning under GA – any implant displacement that requires surgical correction to restore its position

In hospital complication – any complication that occurs during the patient's initial hospital stay at the time of their reconstructive surgery. This includes systematic complications such as DVT/PE and procedure specific complications such as haematoma.

Major complication - Any complication requiring readmission to hospital or return to theatre

Minor complication - Any other complication

Surgeons will be asked to report any learning arising from complications and whether they would be willing to be contacted to discuss this in more detail.
