# Supplementary material for: The Pre-BRA (pre-pectoral Breast Reconstruction EvAluation) feasibility study: protocol for a mixed-methods IDEAL 2a/2b prospective cohort study to determine the safety and effectiveness of prepectoral implant-based breast reconstruction
Source: BMJ Open. 2020 Jan 26;10(1):e033641. doi: 10.1136/bmjopen-2019-033641 (PMC7044855; doi:10.1136/bmjopen-2019-033641)
Supplement: Supplementary data [file bmjopen-2019-033641supp004.pdf]

The Pre-BRA (Pre-pectoral Breast Reconstruction Evaluation) Feasibility Study: Protocol
KL Harvey, N Mills, P White, C Holcombe, S Potter
Appendix 4.

Appendix 4.

The Pre-Bra Study: Surgeon Survey

This survey is for surgeons performing pre-pectoral reconstruction who plan to recruit patients to the Pre-Bra study.

<u>Part 1: Your Details</u>

1. **Your name**……………………………………………………………………………………………
2. **Name of your hospital**…………………………………………………………………………
3. **City where your hospital is based** ……………………………………………………………
4. **What year did you become a consultant surgeon/ associate specialist?**……………………
5. **Do you perform pre-pectoral breast reconstruction (PPBR)?** Yes ☐ No ☐

   (If you answered 'no" to question 5, no further answers are required. Thank you, you are not eligible to complete the questionnaire)

6. **If yes, how long have you been practising the technique of PPBR?**

   <6 months ☐ 2-5 years ☐

   6 -12 months ☐ 5-10 years ☐

   1-2 years ☐ > 10 years ☐

7. **a. IF <6 months or 6-12 months selected:** approximately how many PPBR have you performed to date?

   0-5 ☐ 11-20 ☐ 51-100 ☐

   6-10 ☐ 21-50 ☐ >100 ☐

   b. OR, IF >1 year selected: How many pre-pectoral reconstructions do you perform annually?

The Pre-BRA (Pre-pectoral Breast Reconstruction Evaluation) Feasibility Study: Protocol
KL Harvey, N Mills, P White, C Holcombe, S Potter
Appendix 4.

0-5	☐	11-20	☐	51-100	☐
6-10	☐	21-50	☐	>100	☐

8. **Have you had training or mentorship in the technique of pre-pectoral reconstruction?**

Yes ☐ No ☐

9. **If you have answered yes to Q8. RedCAP^{TM} offers the following options:**

As a trainee? ☐ **<u>OR</u>** As a consultant? ☐

10. **If you have answered yes to Q8. RedCAP^{TM} offers the following options, please tick all that apply:**

A National TIG Fellowship ☐

A training course ☐

An industry workshop/study day ☐

Visit to an established PPBR centre before performing your first case(s) ☐

Experienced PPBR surgeon-mentor in theatre during your first case(s) ☐

Another Oncoplastic fellowship (details below please) ☐

………

………

Other (please state) ☐ …………………………………………………………………….............

………

………

The Pre-BRA (Pre-pectoral Breast Reconstruction Evaluation) Feasibility Study: Protocol
KL Harvey, N Mills, P White, C Holcombe, S Potter
Appendix 4.

Part 2: Your Patient Selection Criteria

11. Which factors affect your decision-making when offering a patient PPBR? (please tick all that apply)

Breast size ☐ Patient age ☐

Patient BMI ☐ Degree of ptosis ☐

Patient hobbies or levels of activity ☐

Patient preference (e.g. to avoid implant animation) ☐

Likely need for post-mastectomy radiotherapy ☐

12. Which (if any) of the following would you consider a contraindication to pre-pectoral breast reconstruction? (please tick all that apply, RedCAP matrix)

	Not a contraindication	Relative contraindication	Absolute contraindication
Current smoker			
BMI >30			
Grade 3 or 4 ptosis			
Predicted implant volume >600 cc			
Breast size D cup or more			
Likely requirement for post-mastectomy radiotherapy			
Previous radiotherapy			
Thin skin flaps (e.g. pinch test <1cm)			

The Pre-BRA (Pre-pectoral Breast Reconstruction Evaluation) Feasibility Study: Protocol
KL Harvey, N Mills, P White, C Holcombe, S Potter
Appendix 4.

Diabetes			
Steroid use			
Connective tissue disease			

Part 3: Your Peri-operative Practice

13. What mesh(es) or ADMs do you use? (Please tick all that apply)

BRAXON ☐ TiLOOP ☐ MESO ☐

Strattice ☐ Fortiva ☐ TIGR ☐

Surgimend ☐ Artia ☐ Other ☐

(If other please give details –name and manufacturer)...

....

14. What is your mesh of choice/ preference? ....

....

15. Would you try a new mesh? Yes ☐ No ☐ Maybe ☐

16. If yes, what would make you consider trying something new?

Evaluation within a protocoled study ☐

Industry representative (rep) recommendation ☐

Evidence of Safety ☐

Any other reason ☐ ....

....

The Pre-BRA (Pre-pectoral Breast Reconstruction Evaluation) Feasibility Study: Protocol
KL Harvey, N Mills, P White, C Holcombe, S Potter
Appendix 4.

17. What implant(s) do you use? (Please tick all that apply)

Mentor	☐	Nagor	☐	B-Lite	☐
Allergan	☐	Eurosurgical	☐	Sebbin	☐

Other ☐ (please give details –name and manufacturer).......................................

....

18. Do you use tissue expanders in the PPBR population? Yes ☐ No ☐

19. If yes, how? (please select all that apply)

☐ I routinely perform two-stage PPBR with tissue expanders in all/ most cases

☐ I use two-stage surgery with tissue expanders in some patients I consider to be high risk (i.e. this is your planned pre-operative strategy)

☐ I convert to two-stage surgery with tissue expanders in some patients I consider to be high risk (i.e. this is an intra-operative decision based upon patient factors such as skin flaps)

☐ I use tissue expanders for PPBR in another way (please give details)

....

....

20. Do you have a protocol for the management of patients undergoing pre-pectoral reconstruction? (e.g. re: drains and antibiotics) Yes ☐ No ☐

21. How many post-operative drains to you routinely use?

None ☐ One ☐ Two ☐

other (please state) ☐……………………………………………………………………………..

The Pre-BRA (Pre-pectoral Breast Reconstruction Evaluation) Feasibility Study: Protocol
KL Harvey, N Mills, P White, C Holcombe, S Potter
Appendix 4.

……………………………………………………………………………………………………….

22. **If one/ two/ other: How long do the drains stay in?** (free text)………………………………

……………………………………………………………………………………………………….

23. **What antibiotics do you use (name and dose) and for how long**?..................................

……

24. **Do you routinely use any of the following strategies to reduce infection? (Please tick all that apply):**

Laminar flow theatre	☐	'Clean' drapes	☐
Pocket wash	☐	'Clean' instruments	☐
Tunnelling of drains	☐		

Refreshing/trimming of skin edges prior to closure ☐

Glove change prior to implant insertion ☐

Part 4: Your Post-operative Practice

25. **How many routine post-operative attendances are your implant-reconstruction patients invited to attend?**

One	☐	Three	☐
Two	☐	>3 times	☐
Varies between cases	☐		

Other…………………………………………………………………………………………………

26. **When do you see patients in the early post-operative period?** (please select all that apply)

Before 1 week	☐	At 1 week	☐

The Pre-BRA (Pre-pectoral Breast Reconstruction Evaluation) Feasibility Study: Protocol
KL Harvey, N Mills, P White, C Holcombe, S Potter
Appendix 4.

At 2 weeks	☐	At 6 weeks	☐
At 3 weeks	☐	Beyond 6 weeks	☐
At 4 weeks	☐	It varies between cases	☐
At 5 weeks	☐	No routine follow-up	☐

Other ☐…………………………………………………………………………………………

Part 5: Interviews with Surgeons

The study team are interested in identifying best practice for pre-pectoral breast reconstruction and would like to speak to surgeons performing the technique to understand how they do the operation, what works well and why.

27. Would you be prepared to be contacted by the study team to take part in a brief interview to discuss your approach to pre-pectoral breast reconstruction? All interviews will be conducted by telephone, should take no more than 20 minutes and information collected will be anonymised. Yes ☐ No ☐

28. If yes, please supply an Email address…………………………………………………………

END

Thank you for your answers
