# Supplementary material for: The Pre-BRA (pre-pectoral Breast Reconstruction EvAluation) feasibility study: protocol for a mixed-methods IDEAL 2a/2b prospective cohort study to determine the safety and effectiveness of prepectoral implant-based breast reconstruction
Source: BMJ Open. 2020 Jan 26;10(1):e033641. doi: 10.1136/bmjopen-2019-033641 (PMC7044855; doi:10.1136/bmjopen-2019-033641)
Supplement: Author's manuscript [file bmjopen-2019-033641.draft_revisions.pdf]

BMJ Open

BMJ Open is committed to open peer review. As part of this commitment we make the peer review history of every article we publish publicly available.

When an article is published we post the peer reviewers' comments and the authors' responses online. We also post the versions of the paper that were used during peer review. These are the versions that the peer review comments apply to.

The versions of the paper that follow are the versions that were submitted during the peer review process. They are not the versions of record or the final published versions. They should not be cited or distributed as the published version of this manuscript.

BMJ Open is an open access journal and the full, final, typeset and author-corrected version of record of the manuscript is available on our site with no access controls, subscription charges or pay-per-view fees (http://bmjopen.bmj.com).

If you have any questions on BMJ Open's open peer review process please email editorial.bmjopen@bmj.com

BMJ Open

The Pre-BRA (Pre-pectoral Breast Reconstruction Evaluation) Feasibility Study: Protocol for a mixed-methods IDEAL 2a/2b prospective cohort study to determine the safety and effectiveness of pre-pectoral implant-based breast reconstruction

Journal:	*BMJ Open*
Manuscript ID	bmjopen-2019-033641
Article Type:	Protocol
Date Submitted by the Author:	14-Aug-2019
Complete List of Authors:	Harvey, Kate; University of Bristol, Centre for Surgical Research, Bristol Medical School<br>Mills, Nicola; University of Bristol, Department of Social Medicine<br>White, Paul; University of the West of England, Faculty of Environment and Technology<br>Holcombe, Christopher; Royal Liverpool and Broadgreen University Hospitals NHS Trust, Linda McCartney Breast Unit<br>Potter, Shelley; University of Bristol,
Keywords:	Breast surgery < SURGERY, Breast tumours < ONCOLOGY, Protocols & guidelines < HEALTH SERVICES ADMINISTRATION & MANAGEMENT, Quality in health care < HEALTH SERVICES ADMINISTRATION & MANAGEMENT

SCHOLARONE™
Manuscripts

The Pre-BRA (*Pre*-pectoral *Breast Reconstruction Evaluation*) Feasibility Study: Protocol for a mixed-methods IDEAL 2a/2b prospective cohort study to determine the safety and effectiveness of pre-pectoral implant-based breast reconstruction

Kate L Harvey[1], Nicola Mills[1], Paul White[2], Chris Holcombe[3], Shelley Potter[1,4] on behalf of The Pre-BRA Feasibility Study Steering Group*

[1]National Institute for Health Research Bristol Biomedical Research Centre, University Hospitals Bristol NHS Foundation Trust and University of Bristol.

[2]Applied Statistics Group, University of the West of England, Bristol, UK

[3]Breast Unit, Royal Liverpool University Hospital, Prescot Street, Liverpool, L7 8XP, UK

[4]Bristol Breast Care Centre, North Bristol NHS Trust, Southmead Road, Bristol, BS10 5NB, UK

*Members of the Pre-BRA steering group are listed at the end of the manuscript and are PUBMED citable

Correspondence to: Kate Harvey, National Institute for Health Research Bristol Biomedical Research Centre, University Hospitals Bristol NHS Foundation Trust and University of Bristol., Whatley Road, Bristol, BS8 2PS

kate.harvey@bristol.ac.uk

Keywords: Pre-pectoral breast reconstruction, IDEAL, cohort study, mixed methods, safety

Word count: 3654

For peer review only

ABSTRACT

Introduction: Implant-based breast reconstruction (IBBR) is the most commonly-performed reconstructive technique worldwide. Sub-pectoral reconstruction with mesh is the current standard of care but new pre-pectoral techniques have recently been introduced. Pre-pectoral breast reconstruction (PPBR) may improve outcomes for patients but robust evaluation is required. Randomised clinical trials (RCTs) are ideally-needed but the short-term safety of PPBR is yet to be established; the technique and its indications are evolving and it has yet to be adopted by a sufficient number of surgeons for an RCT to be feasible.

Pre-BRA aims to determine the feasibility of using mixed-methods within an IDEAL 2a/2b study to explore the short-term safety of PPBR and determine when the technique is sufficiently stable for evaluation in a pragmatic RCT.

Methods and analysis: Pre-BRA is an IDEAL stage 2a/2b prospective multicentre cohort study with embedded qualitative research.

Consecutive patients electing to undergo immediate PPBR at participating centres will be invited to participate. Demographic, operative, oncology and complication data will be collected and patient-reported outcomes will be assessed at baseline, 3 and 18 months post-operatively. The primary safety endpoint will be implant loss at 3 months.

Surgeons performing PPBR will be asked to complete questionnaires regarding their practice and report any modifications made to the procedure or learning arising from complications via free-text response fields on electronic case-report forms. Semi-structured will explore surgeons' experiences in detail to identify emerging best practice. This will be fed back to participating surgeons to promote shared learning.

The Pre-BRA study will aim to recruit 341 patients from 30-40 UK centres over a 12-month period. Recruitment will commence Spring 2019.

For peer review only - http://bmjopen.bmj.com/site/about/guidelines.xhtml

Ethics and dissemination

The study has full ethical approval from OXFORD-B South Central Committee Ref:19/SC/0129. Results will be presented at national and international meetings and published in peer-reviewed journals.

Study registration: ISRCTN11898000

For peer review only

For peer review only - http://bmjopen.bmj.com/site/about/guidelines.xhtml

ARTICLE SUMMARY:

Strengths and Limitations of This Study

- Multicentre prospective cohort study which will generate high-quality data to support the safety of a novel approach to implant-based breast reconstruction prior to undertaking a definitive RCT.
- The study will assess the feasibility of using an innovative mixed-methods approach to promote shared surgical learning and establish when a novel surgical intervention is sufficiently stable for evaluation in the context of a RCT.
- The Pre-BRA Study is a single arm study without an in-built comparison group but the results will be compared safety data from recently published iBRA study and national quality standards.
- The feasibility of using mixed-methods to share learning and determine when the procedure has stabilised will depend on surgeon engagement and the feasibility of this approach is yet to be explored.

For peer review only

INTRODUCTION

Up to 40%[1] of the 55,000[2] women diagnosed with breast cancer each year in the UK will require a mastectomy and of these, approximately one in four will elect to have an immediate breast reconstruction[3] to improve their quality of life[4]. Implant-based breast reconstruction is the most commonly performed technique world-wide[5,6] and sub-pectoral implant-based breast reconstruction (IBBR) with biological or synthetic mesh has become the standard of care despite the lack of high-quality data to support the safety or effectiveness of the technique[7,8].

Recently, however, mesh-based techniques have evolved further with early studies[9] suggesting good results when fixed-volume implants are completely covered in a biological[10-12] or synthetic mesh[13-16] and placed on top of the muscle in a pre-pectoral position. This 'muscle-sparing' technique may result in less post-operative pain, more natural results and may prevent 'implant animation', the upwards movement of the implant seen when the pectoral muscle contracts[17].

While early results[18] of this pre-pectoral technique are promising in the hands of a few expert surgeons[10,11,13-15,19,20], historically subcutaneous implant placement without mesh, was abandoned by the reconstructive community due to high complication rates[21-24]. To date, few well-designed prospective studies have directly compared pre and sub-pectoral IBBR techniques[25] and evidence to support the proposed benefits of pre-pectoral implant placement is inconsistent[17,26,27]. There is therefore a need to robustly evaluate pre-pectoral implant reconstruction (PPBR) before it becomes standard practice[18]. Randomised clinical trials (RCTs) provide the best evidence, but the safety of PPBR has yet to be established; the technique and its indications are still evolving, and it has not yet been adopted by a sufficient number of surgeons in the UK for a randomised trial to be feasible. Preliminary work is therefore needed before definitive evaluation can be considered.

The IDEAL (Idea-Development-Exploration-Assessment-Long-term Study) Framework provides recommendations for the evaluation of a surgical innovation from first-in-man to long-

For peer review only - http://bmjopen.bmj.com/site/about/guidelines.xhtml

term study[28,29]. Phases 2a (Development)/2b (Exploration) focus on feasibility work for a definitive future trial including establishing the risks and benefits of the technique; the stability of the intervention, its indications and the adoption of the procedure by a wider group of surgeons from the initial innovators[30]. Whilst the scope and intention of IDEAL is clearly defined, the system is not well operationalised, and methods for the optimal conduct of early-phase surgical studies are unclear. Embedding qualitative methods within an IDEAL study may provide a novel way of enhancing shared learning within the surgical community to reduce learning curve effects and allow a new procedure to be introduced more safely and efficiently. Monitoring and sharing modifications may also provide an effective way of determining when the procedure is sufficiently stable for formal evaluation in the context of a definitive RCT.

METHODS AND ANALYSIS

Primary aim

The aim of Pre-BRA is to determine the feasibility of using mixed-methods within an IDEAL 2a/2b study to explore the short-term safety and effectiveness of pre-pectoral breast reconstruction and determine when it is sufficiently stable for formal evaluation in a definitive RCT.

Specific objectives will be

1. To establish the short-term safety (implant loss; infection; re-operation and readmission at 3 months) and effectiveness (patient-reported outcomes using BREAST-Q at 3 and 18 months) of pre-pectoral breast reconstruction compared with published national standards from the National Mastectomy and Breast Reconstruction Audit (NMBRA)[31] and iBRA[7] studies
2. To explore the feasibility of using a novel mixed-methods approach in an IDEAL 2a/2b study to promote shared learning and identify intervention stability including the acceptability of the methodology to surgeons and the most effective methods for providing feedback

For peer review only - http://bmjopen.bmj.com/site/about/guidelines.xhtml

3. To inform the feasibility, design and conduct of a future RCT including the numbers of patients at participating centres undergoing IBBR who are eligible for PPBR; types of meshes used and approach to concomitant interventions
4. To build capacity to deliver a future RCT by establishing a network of surgeons engaged in the need for evaluation who are able to perform pre-pectoral breast reconstruction to a high standard and who will participate in a future study

Study design

The Pre-BRA Study is a multicentre, IDEAL Phase 2a/2b prospective observational cohort study with embedded qualitative methods.

Patient and public involvement

This project is an extension of the iBRA study[7] which was designed with patient co-applicants. A patient group will be established to review and comment on the results. This patient group will input on the design of the future RCT, in particular, selection of the most appropriate primary outcome measure and timing of assessment.

Setting

All UK breast and plastic surgical centres currently performing pre-pectoral breast reconstruction using any technique will be invited to participate through the UK professional associations (Association of Breast Surgery [ABS], British Association of Plastic Reconstructive and Aesthetic Surgeons [BAPRAS]).

All surgeons currently performing PPBR will be eligible to participate in the study but those wishing to start performing the technique will be encouraged to follow the ABS/BAPARS guidelines regarding mentorship and training[32] prior to entering patients.

Participants

For peer review only - http://bmjopen.bmj.com/site/about/guidelines.xhtml

Inclusion criteria: Consecutive women aged 16 or over who require a mastectomy for breast cancer or risk-reduction who elect to undergo IBBR; are considered technically suitable for PPBR by their surgeon and consent to undergo the procedure are eligible to participate in the study. Patients undergoing PPBR will be informed that the procedure is innovative and that outcome data are limited as part of their discussion with the surgical team.

Exclusion criteria: Absolute and relative exclusion criteria will be used to reflect current practice and account for the variable experience of surgeons performing PPBR across the UK. Absolute exclusion criteria will be applied to all patients. Relative exclusion criteria can be applied flexibly according to the surgeon's experience with the procedure in conjunction with the patient's consent following discussion that the risk of complications may be increased in this setting.

Absolute exclusion criteria will be:

i. Patients assessed as having thin/insufficiently vascularised skin-flaps as assessed by the operating surgeon
ii. Patients having revisional or delayed breast reconstruction
iii. Patients unable or unwilling to provide informed consent.

Relative exclusion criteria reflect ABS/BAPRAS guidelines for mesh-assisted breast reconstruction[32] and include

i. Current smokers or recent ex-smokers (<6 weeks)
ii. Previous breast/chest wall radiotherapy
iii. Body mass index (BMI) of greater than 30
iv. Reconstruction with an implant of more than 600cc
v. Patients anticipated to require post mastectomy radiotherapy
vi. Poorly-controlled diabetes

For peer review only - http://bmjopen.bmj.com/site/about/guidelines.xhtml

Participant identification, screening and recruitment

All female patients aged 16 or over undergoing mastectomy for breast cancer or risk-reduction who elect to undergo immediate IBBR will be screened to inform the feasibility of a future trial comparing different approaches to IBBR.

Participant screening

Potential participants will be identified from clinics and multidisciplinary meetings by the clinical and research teams.

A comprehensive screening log will be maintained at participating sites of all women who elect to undergo immediate IBBR under the care of participating surgeons to determine the proportion of patients who:

i. Are considered technically suitable for pre-pectoral technique by the operating surgeon.
ii. For those patients who are considered not suitable for PPBR – with reasons for this (e.g. relative exclusion criteria: smoker; previous RT etc. or other reason).
iii. For patients considered technically suitable for PPBR, the proportion who accept the technique.
iv. For those patients accepting PPBR, the proportion who consent to study participation

Patient recruitment

Patients who elect to undergo PPBR will be given a patient information sheet (PIS) explaining the study. They will be seen at a follow up appointment by a member of the clinical or research team and asked to sign a consent form prior to participating in the study. (Appendix 4.) Patients will be allocated a study ID number.

For peer review only - http://bmjopen.bmj.com/site/about/guidelines.xhtml

Patients will be given a date for surgery as per unit protocol and undergo pre-operative assessment. They will be required to complete baseline patient-reported outcome questionnaires (BREAST-Q) and have pre-operative photographs as per unit policy prior to surgery.

Study procedures

All patients in whom pre-pectoral reconstruction with or without mesh is planned will be eligible for inclusion in the study. All meshes must be licensed for use in the UK and have a CE mark. Composite pre-pectoral reconstructions using dermal sling and mesh or two different types of mesh will be permitted and the details of products used will be recorded. Reconstructions using full dermal sling coverage will also be included.

Standardisation of surgical technique and procedure fidelity

All patients will undergo a skin or nipple-preserving mastectomy followed by an immediate implant-based breast reconstruction.

Participating surgeons will undertake the procedure as per their standard practice. Mesh choice (biological or synthetic and the product used) and implant selection (fixed-volume; adjustable implants or tissue expanders) will be as per surgeon preference. All patients will be planned for a pre-pectoral reconstruction prior to surgery.

The following steps of the PPBR procedure will be considered mandatory, prohibited and discretionary according to the typology described by Blencowe et al.[33]

- **Mandatory** – Insertion of a tissue expander/adjustable implant or fixed-volume implant
- **Prohibited** –Raising the pectoralis muscle
- **Discretionary** – All other steps of the procedure

If the surgeon considers that pre-pectoral reconstruction is not possible at the time of surgery or modifies the planned procedure (e.g. inserts tissue expander rather than fixed-volume

For peer review only - http://bmjopen.bmj.com/site/about/guidelines.xhtml

implant), this will be recorded on the case report form (CRF) with reasons for the modification (e.g. thin skin-flaps). Details of the alternative procedure performed (e.g. sub-pectoral reconstruction) will be collected. Surgeons will also be encouraged to record any learning arising from the case.

Strategies to minimise infection (e.g. use of laminar flow, cavity irrigation, glove change) will be as per local practice but participating centres will be encouraged to adhere to published best practice guidelines[32,34] and use the evidence-based Manchester checklist[35].

Use of drains and other concomitant interventions (e.g. antibiotics, dressings) will be permitted as per local practice.

Deviations from planned pre-pectoral reconstruction

As detailed above, surgeons will be asked to report the following via the CRF:

a. Patients in whom PBR was planned but had to be abandoned completely (i.e. another form of reconstruction or no reconstruction performed)

    i. Reasons why pre-pectoral approach was abandoned
    ii. Procedure performed instead
    iii. Any reflections or learning regarding the case

b. Patients in whom the planned procedure was modified (but pre-pectoral reconstruction still performed)

    i. Details of the modification made (e.g. insertion of tissue expander rather than fixed volume implant)
    ii. Reasons for the modification (e.g. thin skin flaps)
    iii. Any reflections or learning regarding the case

For peer review only - http://bmjopen.bmj.com/site/about/guidelines.xhtml

Outcome measures

Safety and effectiveness outcomes

The primary outcome for the pre-pectoral safety study will be implant loss at 3 months defined as the removal of the implant or tissue-expander without replacement as a result of a complication. This is consistent with the definition used in the iBRA study[7]. Secondary safety and effectiveness outcomes will be:

i. Post-operative pain assessed by patient self-report using a visual analogue scale at 24 hours, 1 week, 2 weeks and 3 months
ii. Complications requiring readmission and/or re-operation at 3 months
iii. Infection defined as i) minor – requiring treatment with oral antibiotics ii) major requiring admission for intravenous antibiotics and/or surgical debridement
iv. Quality of life including satisfaction with breasts; physical well-being; psychosocial well-being and animation assessed using the validated BREAST-Q[36] questionnaire at 3 and 18 months following surgery.
v. Further surgery for complications or cosmesis at 18 months

Feasibility outcomes

Surgeon engagement will be used to assess the feasibility of using mixed-methods to explore learning and intervention stability in the study. At study entry, the number of surgeons completing the surgeon questionnaire; the number of surgeons consenting to be contacted for interview and the number of surgeons interviewed will be compared. For modifications/learning arising from complications, the numbers of events (modifications and complications) reported will be compared with numbers of free-text responses entered; the number of surgeons consenting to interview and the actual number interviewed. The content of the free-text responses and interviews will also be assessed.

For peer review only - http://bmjopen.bmj.com/site/about/guidelines.xhtml

Screening logs will be reviewed to determine the relative proportions of patients electing to undergo IBBR who are i.) considered technically suitable for pre-pectoral reconstruction and ii) elect to undergo surgery to determine if a full-scale pragmatic RCT is feasible.

Data collection

Clinical data

Demographic, comorbidity and operative data will be collected for each participant via a standardised electronic case report form (CRF) hosted on REDCap[37] (Appendix 1).

Complications and oncological data will be collected at 30 days and 3 months by clinical or case-note review according to local follow up policies. Details of any additional surgery required for complications or cosmesis will be collected at 18 months by case note review. No additional clinic visits will be required for the study. All complications will be defined a priori using standardised definitions as per previous studies[7,38] (Appendix 2*)*

Patient-reported outcomes

Participants will be asked to complete the pre-operative breast reconstruction BREAST-Q questionnaire prior to surgery.

Post-operative pain scores will be collected by patient self-report using an electronic visual analogue scale at 24 hours, 1 week, 2 weeks and 3 months following surgery.

Patient satisfaction with breasts, physical well-being, psychosocial well-being, satisfaction with implants and patient-reported assessment of animation will be assessed at 3 and 18 months using the validated BREAST-Q questionnaire[36]. A reminder will be sent after one month if no response is received. Patients will be encouraged to complete on-line PROMs but paper versions will be made available to patients unable or unwilling to use electronic versions.

For peer review only - http://bmjopen.bmj.com/site/about/guidelines.xhtml

Shared learning and intervention stability

Best practice and opportunities for shared learning among participating surgeons will occur at the following points in the study.

i. Study entry

ii. As a result of procedure modifications or complications

Surgeons electing to participate in the study will be sent a surgeon information sheet outlining the proposed shared learning study and what participation may involve. Surgeons will be able to recruit patients to Pre-BRA without contributing to shared learning but full participation will be encouraged by the steering group.

At each time point a combination of free-text responses on standardised CRFs and focussed semi-structured qualitative interviews will be to explore group learning and intervention stability.

i. Study entry

Phase 1 - Surgeon questionnaire

Prior to commencing patient recruitment, participating surgeons will be asked to complete a surgeon questionnaire. The questionnaire (appendix 3) will explore techniques and products used; experience; approaches to concomitant interventions and patient selection criteria. The questionnaire will include consent to be contacted regarding a brief (20 min) semi-structured qualitative interview exploring their practice in more detail.

Phase 2 – Semi-structured study entry interviews

Sampling and data collection

For peer review only - http://bmjopen.bmj.com/site/about/guidelines.xhtml

A purposive sample of surgeons consenting to interview will be selected based on techniques and products used; experience; geographical location and differing approaches to concomitant interventions and patient selection.

Interviews will be organised at a time convenient to the participating surgeons. All interviews will be conducted by telephone using a semi-structured topic guide developed based on the literature and clinical expertise to explore issues surrounding current practice. All interviews will be audio-recorded and transcribed verbatim. Interview participants will be asked to give verbal consent to study participation and audio-recording prior to commencing the interview. The topic guide will be modified iteratively as the study progresses to allow new themes to emerge. Sampling, data collection and analysis will be conducted concurrently and iteratively until data saturation is achieved and no new themes emerge from the data.

ii. As a result of procedure modifications and learning from complications

Phase 1 – Case report forms

Surgeons will be asked to report (yes/no) whether i) a modification to the planned procedure was made at the time of the operation and ii) any learning (e.g. changes to future surgical technique) occurred as a result of a complication at 3 months. If modifications or learning as a result of complications are reported, surgeons will be asked to provide details in a free text response box on the electronic CRF and to indicate whether they would be willing to be contacted to participate in a brief telephone interview to explore this in more detail.

Phase 2 - Semi-structured surgeon modification/complication interviews

Sampling and data collection

Surgeons providing consent will be contacted to arrange a brief semi-structured qualitative interview. Interviews will be conducted as per study entry interviews outlined above but will

For peer review only - http://bmjopen.bmj.com/site/about/guidelines.xhtml

focus specifically on the modification/learning arising from the complication including details, rationale and outcome.

The feasibility of this approach is unknown so initially, it is planned that all surgeons reporting procedure modifications/learning from complications and providing consent will be interviewed. As the study progresses, depending on the number and content of the CRF reports, all consenting surgeons may continue to be interviewed or, it may be necessary to purposively sample consenting surgeons based on the details of the modification/learning reported in the CRF to explore new and emerging themes and allow the details of the modifications to be explored. Data collection will continue until no new learning or modifications emerge from the data.

Data analysis and sample size

Pre-pectoral safety and effectiveness

Sample Size

A single-arm 2-stage design will be used to assess the safety of PPBR based on the primary outcome of implant loss rate at 3 months. It is expected that the implant loss rate will be 9% or lower based on findings from the recent iBRA study[7]. 15% or greater will be deemed unacceptably high.

A sample size of 310 patients would result in a two-sided 95.0 % confidence interval for a single proportion, assumed to be 0.10, with a width equal to 0.070. Allowing for a 10% loss to follow-up at 3 months at least 341 patients will be recruited to inform a future trial with implant loss as the primary outcome.

Based on data from the iBRA study, the number of pre-pectoral reconstructions performed at each site is likely to be small (4-40 per year)[7]. We will therefore aim to recruit from between 20 and 30 sites over a period of 12 months.

For peer review only - http://bmjopen.bmj.com/site/about/guidelines.xhtml

For peer review only

Statistical analysis

Simple summary statistics will be calculated to describe demographic, procedure, process and outcome data. Categorical data will be summarised by counts and percentages and continuous data by median, interquartile range and range.

We will establish the proportion and 95% confidence intervals of patients experiencing implant loss and other key safety outcomes (re-admission for complications, re-operation and infection) at three months and compare these with findings with those reported in the iBRA study[7] .

An exploratory risk factor analysis will be conducted to explore the impact of type of mesh and known risk factors for complications including smoking; BMI and radiotherapy if sufficient numbers of patients are recruited to the study.

Planned interim analysis

A planned interim analysis will be performed when 155 patients have been recruited to the study and followed-up for a minimum of 3 months. The report will be reviewed by the study steering group and the results will be made available to the study sponsor.

If the overall implant loss rate at 3 months following surgery is 15% or more, the study will be stopped.

Shared learning and intervention stability feasibility study

Analysis of the free-text responses will be an ongoing and iterative process commencing soon after the study begins and will inform sampling for the semi-structured interview phase of the study. Text will be assigned codes and analysed using the constant comparative technique of grounded theory[39]. A combination of thematic and content analysis will be used to identify common themes[40,41]. These will inform the topic guide for the interviews and the feedback provided to study participants.

For peer review only - http://bmjopen.bmj.com/site/about/guidelines.xhtml

Sampling, data collection and analysis will be undertaken concurrently and iteratively until no new themes emerge from the data.

Dissemination of best practice and shared learning

The learning and modifications emerging from the CRFs and interviews will be consolidated and reviewed at regular intervals by the study team. Any common themes will be shared with participating surgeons using a combination of methods. These will include a ‘tips and tricks’ section on the study website; e-mail updates; study newsletters and potentially social media channels (e.g. ‘You Tube’). The acceptability and value of the different approaches to sharing learning will be discussed with individual surgeons at interview and the wider steering group. Metrics such as number of views/hits for online/social media resources will used to provide additional information regarding engagement if appropriate.

Design of the main study

If PPBR is considered safe and data from the screening logs suggests sufficient numbers of PPBR are being performed at participating centres, a consensus meeting with key participating surgeons, steering group members, patients and methodologists will be held to agree the final design for a large-scale pragmatic RCT comparing pre-pectoral and sub-pectoral implant-based breast reconstruction.

ETHICS AND DISSEMINATION

Full ethical approval has been obtained for the study (NRES OXFORD-B South Central Committee Ref:19/SC/0129. IRAS ID: 255421) and written informed consent will be obtained for study participation. All potential study participants will be informed that pre-pectoral reconstruction is a new technique and that outcome data is limited. An interim analysis is planned and if implant loss rates exceed 15%, the study will be stopped.

For peer review only

For peer review only - http://bmjopen.bmj.com/site/about/guidelines.xhtml

Results will be analysed and presented at national and international meetings and published in peer-reviewed journals. Findings will be fed back to the surgical community to promote engagement and recruitment to a future RCT.

AUTHORS' CONTRIBUTIONS

SP and CH conceived the study. KH, SP, NM, CH contributed to the study design. PW provided statistical support. SP, CH and KH secured funding for the project. KH wrote the paper, SP critically revised the manuscript. All authors read, critically revised and approved the manuscript before submission.

Members of the Pre-BRA Feasibility Study Steering group in alphabetical order are:

Peter Barry, Simon Cawthorn, Matthew Gardiner, Gareth Irwin, Cliona Kirwan, Mairead McKenzie, Shireen McKenzie, Rachel O'Connell, Georgette Oni, Tim Rattay, Pankaj Roy, Joanna Skillman, Soni Soumian, Raghavan Vidya, Lisa Whisker and Samantha Williams.

STUDY SPONSOR

The University of Bristol.

Research Governance Officer: Ms Anna Brooke: research-governance@bristol.ac.uk

DATA ACCESS STATEMENT

Data are available upon reasonable request. De-identified participant data from the safety study will be available from the senior author after completion of the study and planned analyses following review by the study steering group. Reuse will be permitted with consent of the study steering group. No additional information will be available. Data from the surgeon interviews will not be made available due to the sensitive nature of the information collected and the ability to identify study participants.

FUNDING STATEMENT

For peer review only - http://bmjopen.bmj.com/site/about/guidelines.xhtml

This work was supported by a One Year Royal College of Surgeons Blond McIndoe Foundation Research Fellowship, the Association of Breast Surgery and the NIHR Biomedical Research Centre at University Hospitals Bristol NHS Foundation Trust and the University of Bristol. SP is an NIHR Clinician Scientist (CS-2016-16-019). The views expressed are those of the authors and not necessarily those of the NIHR or the Department of Health and Social Care.

COMPETING INTERESTS STATEMENT

The authors have no conflict of interest to declare.

REFERENCES

1. Matala CM, McIntosh SA, Purushotham AD. Immediate breast reconstruction after mastectomy for cancer. *Br J Surg* 2000; **87**: 1455-72.

2. Cancer Research UK. https://www.cancerresearchuk.org/health-professional/cancer-statistics/statistics-by-cancer-type/breast-cancer#heading-Zero. 2018 (accessed 18th November 2018.

3. Jeevan R, Mennie JC, Mohanna PN, O'Donoghue JM, Rainsbury RM, Cromwell DA. National trends and regional variation in immediate breast reconstruction rates. *Br J Surg* 2016; **103**(9): 1147-56.

4. Harcourt D, Rumsey N, Ambler N, et al. The psychological effect of mastectomy with or without breast reconstruction: A prospective, multicentre study. *Plastic & Reconstructive Surgery* 2003; **111**(3): 1060-8.

5. Mennie JC, Mohanna PN, O'Donoghue JM, Rainsbury R, Cromwell DA. National trends in immediate and delayed post-mastectomy reconstruction procedures in England: A seven-year population-based cohort study. *Eur J Surg Oncol* 2017; **43**(1): 52-61.

6. Albornoz CR, Bach PB, Mehrara BJ, et al. A paradigm shift in U.S. Breast reconstruction: increasing implant rates. *Plast Reconstr Surg* 2013; **131**(1): 15-23.

For peer review only - http://bmjopen.bmj.com/site/about/guidelines.xhtml

7. Potter S, Conroy EJ, Cutress RI, et al. Short-term safety outcomes of mastectomy and immediate implant-based breast reconstruction with and without mesh (iBRA): a multicentre, prospective cohort study. *The Lancet Oncology* 2019; **20**(2): 254-66.

8. Potter S, Browning D, Savovic J, Holcombe C, Blazeby JM. Systematic review and critical appraisal of the impact of acellular dermal matrix use on the outcomes of implant-based breast reconstruction. *British Journal of Surgery* 2015; **102**(9): 1010-25.

9. Salibian AA, Frey JD, Choi M, Karp NS. Subcutaneous Implant-based Breast Reconstruction with Acellular Dermal Matrix/Mesh: A Systematic Review. *Plastic and Reconstructive Surgery – Global Open* 2016; **4**(11): e1139.

10. Berna G, Cawthorn SJ, Papaccio G, Balestrieri N. Evaluation of a novel breast reconstruction technique using the Braxon® acellular dermal matrix: a new muscle-sparing breast reconstruction. *ANZ Journal of Surgery* 2014: n/a-n/a.

11. Reitsamer R, Peintinger F. Prepectoral implant placement and complete coverage with porcine acellular dermal matrix: A new technique for direct-to-implant breast reconstruction after nipple-sparing mastectomy. *Journal of Plastic, Reconstructive & Aesthetic Surgery* 2015; **68**(2): 162-7.

12. Zhu L, Mohan AT, Abdelsattar JM, et al. Comparison of subcutaneous versus submuscular expander placement in the first stage of immediate breast reconstruction. *Journal of Plastic, Reconstructive & Aesthetic Surgery* 2016; **69**(4): e77-e86.

13. Casella D, Bernini M, Bencini L, et al. TiLoop® Bra mesh used for immediate breast reconstruction: comparison of retropectoral and subcutaneous implant placement in a prospective single-institution series. *European Journal of Plastic Surgery* 2014; **37**(11): 599-604.

14. Casella D, Calabrese C, Bianchi S, Meattini I, Bernini M. Subcutaneous Tissue Expander Placement with Synthetic Titanium-Coated Mesh in Breast Reconstruction: Long-term Results. *Plastic and Reconstructive Surgery – Global Open* 2015; **3**(12): e577.

For peer review only

For peer review only - http://bmjopen.bmj.com/site/about/guidelines.xhtml

15. Bernini M, Calabrese C, Cecconi L, et al. Subcutaneous Direct-to-Implant Breast Reconstruction: Surgical, Functional, and Aesthetic Results after Long-Term Follow-Up. *Plastic and Reconstructive Surgery – Global Open* 2015; **3**(12): e574.

16. Kobraei EM, Cauley R, Gadd M, Austen WGJ, Liao EC. Avoiding Breast Animation Deformity with Pectoralis-Sparing Subcutaneous Direct-to-Implant Breast Reconstruction. *Plastic and Reconstructive Surgery – Global Open* 2016; **4**(5): e708.

17. Cattelani L, Polotto S, Arcuri MF, Pedrazzi G, Linguadoca C, Bonati E. One-Step Prepectoral Breast Reconstruction With Dermal Matrix-Covered Implant Compared to Submuscular Implantation: Functional and Cost Evaluation. *Clinical breast cancer* 2018; **18**(4): e703-e11.

18. Tasoulis MK, Iqbal FM, Cawthorn S, MacNeill F, Vidya R. Subcutaneous implant breast reconstruction: Time to reconsider? *European Journal of Surgical Oncology (EJSO)* 2017.

19. Becker H, Lind JGI, Hopkins EG. Immediate Implant-based Prepectoral Breast Reconstruction Using a Vertical Incision. *Plastic and Reconstructive Surgery – Global Open* 2015; **3**(6): e412.

20. Vidya R, Masià J, Cawthorn S, et al. Evaluation of the effectiveness of the prepectoral breast reconstruction with Braxon dermal matrix: First multicenter European report on 100 cases. *The Breast Journal*: n/a-n/a.

21. SNYDERMAN RK, GUTHRIE RH. RECONSTRUCTION OF THE FEMALE BREAST FOLLOWING RADICAL MASTECTOMY. *Plastic and Reconstructive Surgery* 1971; **47**(6): 565-7.

22. Gruber RP, Kahn RA, Lash H, Maser MR, Apfelberg DB, Laub DR. Breast Reconstruction Following Mastectomy: A Comparison of Submuscular and Subcutaneous Techniques. *Plastic & Reconstructive Surgery* 1981; **67**(3): 312-7.

23. Rebowe RE, Allred LJ, Nahabedian MY. The Evolution from Subcutaneous to Prepectoral Prosthetic Breast Reconstruction. *Plastic and reconstructive surgery Global open* 2018; **6**(6): e1797.

For peer review only - http://bmjopen.bmj.com/site/about/guidelines.xhtml

For peer review only

24. Wagner RD, Braun TL, Zhu H, Winocour S. A systematic review of complications in prepectoral breast reconstruction. *Journal of Plastic, Reconstructive & Aesthetic Surgery* 2019; **72**(7): 1051-9.

25. Li L, Su Y, Xiu B, et al. Comparison of prepectoral and subpectoral breast reconstruction after mastectomies: A systematic review and meta analysis. *European Journal of Surgical Oncology* 2019.

26. Baker BG, Irri R, MacCallum V, Chattopadhyay R, Murphy J, Harvey JR. A Prospective Comparison of Short-Term Outcomes of Subpectoral and Prepectoral Strattice-Based Immediate Breast Reconstruction. *Plast Reconstr Surg* 2018; **141**(5): 1077-84.

27. Bettinger LN, Waters LM, Reese SW, Kutner SE, Jacobs DI. Comparative Study of Prepectoral and Subpectoral Expander-Based Breast Reconstruction and Clavien IIIb Score Outcomes. *Plastic and reconstructive surgery Global open* 2017; **5**(7): e1433.

28. Ergina PL, Cook JA, Blazeby JM, et al. Challenges in evaluating surgical innovation. *Lancet* 2009; **374**: 1097-104.

29. Pennell CP, Hirst AD, Campbell WB, et al. Practical guide to the Idea, Development and Exploration stages of the IDEAL Framework and Recommendations. *British Journal of Surgery* 2016; **103**(5): 607-15.

30. Hirst A, Phillippou Y, Blazeby J, et al. No Surgical Innovation without Evaluation: Evolution and Further Development of the IDEAL Framework and Recommendations. *Annals of Surgery* 2019; **269**(2): 211-20.

31. Jeevan R, Cromwell DA, Browne JP, et al. Findings of a national comparative audit of mastectomy and breast reconstruction surgery in England. *J Plast Reconstr Aesthet Surg* 2014; **67**(10): 1333-44.

32. Martin L, O'Donoghue JM, Horgan K, et al. Acellular dermal matrix (ADM) assisted breast reconstruction procedures: joint guidelines from the Association of Breast Surgery and the British Association of Plastic, Reconstructive and Aesthetic Surgeons. *European Journal of Surgical Oncology* 2013; **39**(5): 425-9.

For peer review only

For peer review only - http://bmjopen.bmj.com/site/about/guidelines.xhtml

33. Blencowe NS, Mills N, Cook JA, et al. Standardizing and monitoring the delivery of surgical interventions in randomized clinical trials. *British Journal of Surgery* 2016; **103**(10): 1377-84.

34. Rainsbury D, Willett A. Oncoplastic breast reconstruction: guidelines for best practice. UK, 2012.

35. Barr SP, Topps AR, Barnes NL, et al. Infection prevention in breast implant surgery - A review of the surgical evidence, guidelines and a checklist. *Eur J Surg Oncol* 2016; **42**(5): 591-603.

36. Pusic AL, Klassen A, Scott AF, Klok JA, ordeiro P G, Cano S. Development of a new patient-reported outcome measure for breast surgery: the BREAST-Q. *Plastic and Reconstructive Surgery* 2009; **124**(2): 345-53.

37. Harris PA, Taylor R, Thielke R, Payne J, Gonzalez N, Conde JG. Research electronic data capture (REDCap)--a metadata-driven methodology and workflow process for providing translational research informatics support. *J Biomed Inform* 2009; **42**(2): 377-81.

38. O'Connell RL, Rattay T, Dave RV, et al. The impact of immediate breast reconstruction on the time to delivery of adjuvant therapy: the iBRA-2 study. *Br J Cancer* 2019.

39. Glaser B, Strauss AL. The Discovery of Grounded Theory. Chicago: Adeline; 1967.

40. Braun V, Clarke V. Using thematic analysis in psychology. *Qualitative Research in Psychology* 2006; **3**(2): 77-101.

41. Krippendorff K. Values, modes and domains of inquiry into communication. *The Journal of communication* 1969; **19**(2): 105-33.

For peer review only

The Pre-Bra Feasibility Study (*Pre*-pectoral *B*reast *R*econstruction Ev*a*luation)

A mixed-methods IDEAL 2a/2b prospective cohort study to determine the safety and effectiveness of pre-pectoral implant based breast reconstruction

Study Protocol

Version 1.0
19th February 2019

For peer review only

For peer review only - http://bmjopen.bmj.com/site/about/guidelines.xhtml

RESEARCH TEAM

Chief Co-Investigators:	
**Shelley Potter PhD FRCS**<br>Consultant Senior Lecturer in Oncoplastic Breast Surgery, NIHR Clinician Scientist<br>Centre for Surgical Research, Bristol Medical School, Canynge Hall, Whatley Road,<br>Bristol, BS8 2PS<br>shelley.potter@bristol.ac.uk	**Professor Chris Holcombe MD FRCS**<br>Consultant Breast Surgeon<br>Royal Liverpool University Hospital.<br>Prescot Street,<br>Liverpool, L7 8XP<br>chris.holcombe@rlbuht.nhs.uk
**Postgraduate Research Fellow:**<br><br>Kate Harvey MBBS MRCS<br>RCSE Blond-McIndoe Research Fellow<br>Centre for Surgical Research, Bristol Medical School, Canynge Hall, Whatley Road,<br>Bristol, BS8 2PS<br>kate.harvey@bristol.ac.uk	**Patient and Public Involvement Lead:**<br><br>Mairead MacKenzie<br>Independent Cancer Patients' Voice,<br>London,<br>UK<br>mairead@icpv.org.uk
**Pre-Bra Steering Group Members:**<br><br>Simon Cawthorn<br>Tim Rattay<br>Pankaj Roy<br>Soni Soumian<br>Georgette Oni<br>Joanna Skillman<br>Peter Barry<br>Shireen McKenzie<br>Rosie O'Connell<br>Matthew Gardiner<br>Samantha Williams	**Study Statistician:**<br><br>Dr Paul White BSc, MSc, PhD<br>Associate Professor (Applied Statistics)<br>University of the West of England,<br>Bristol<br>paul.white@uwe.ac.uk

For peer review only - http://bmjopen.bmj.com/site/about/guidelines.xhtml

GLOSSARY OF TERMS

AE	Adverse Event
AR	Adverse Reaction
CI	Chief Investigator
CRF	Case Report Form
CTU	Clinical Trials Unit
GP	General Practitioner
HRA	Health Research Authority
HRQL	Health-related quality of life
IB	Investigator's Brochure
IBBR	Implant based breast reconstruction
ICF	Informed Consent Form
IDSMC	Independent Data and Safety and Monitoring Committee
IEC	Independent Ethical Committee
IMP	Investigational Medicinal Product
MREC	Multi-Centre Research Ethics Committee
PI	Principal Investigator
PIS	Patient Information Sheet
PPBR	Pre-pectoral breast reconstruction
PROMS	Patient reported outcomes measures
RCT	Randomised controlled trial
R&D	Research & Development
SAE	Serious Adverse Event
SAR	Serious Adverse Reaction
SSG	Study Steering Group
SUSAR	Suspected Unexpected Serious Adverse Reaction
UAR	Unexpected Adverse Reaction

For peer review only

For peer review only - http://bmjopen.bmj.com/site/about/guidelines.xhtml

TABLE OF CONTENTS

1. PROTOCOL SUMMARY
1.1 Schematic of Study Design

2. DEFINITIONS

3. BACKGROUND

4. AIMS AND OBJECTIVES
4.1. Part I: Pre-Pectoral Reconstruction Safety Study
4.2. Part II: Shared Learning Feasibility Study
4.3. Study Endpoints:
Pre-Pectoral Reconstruction Safety Study Endpoints
Shared Learning Feasibility Study Outcomes

5. METHODS
5.1. Overall Study Design
5.2. Surgeon-Participants:
Inclusion Criteria
Exclusion Criteria
Recruitment and Consent
5.3. Patient-Participants:
Inclusion Criteria
Exclusion Criteria
Screening
Recruitment and Consent

6. DATA COLLECTION PART I:
Pre-Pectoral Reconstruction Safety Study
6.1. Pre-Pectoral Breast Reconstruction
Eligible Meshes and ADM products
Standardisation of Surgical Technique
Components of PPBR
6.2. Case Report Form (CRF)
Assessing Complications
Schedule of Trial Procedures
6.3. Patient Reported Outcome Measures (PROMs):
Shoulder function; physical well-being and satisfaction
Pain Scores
6.4. Protocol Deviations

7. DATA COLLECTION PART II:
Shared Learning Feasibility Study
7.1. Opportunities for Shared Learning
7.2. Study Entry
Surgeon Questionnaire

For peer review only - http://bmjopen.bmj.com/site/about/guidelines.xhtml

Sampling
Semi-Structured Study Entry Interviews
7.3. Following Selection Criteria or Procedure Modifications
Modification Case Report Forms (CRFs)
Sampling
Semi-Structured Modification Interviews
7.4. Following Complications
Complication Case Report Forms (CRFs)
Sampling
Semi-Structured Complication Interviews

8. ANALYSIS PART I:
Pre-Pectoral Reconstruction Safety Study
8.1. Sample Size
8.2. Analytical Methods:
Data Summaries
Patient Groups for Analysis
Missing Data
Other Analyses
8.3. Interim Analysis and Monitoring

9. ANALYSIS PART II:
Shared Learning Feasibility Study
9.1. Analysis of Results
9.2. Distribution of Shared Learning

10. PUBLICATION

11. TRIAL OVERSIGHT
11.1. Study Steering Group (SSG)

12. ETHICAL AND REGULATORY CONSIDERATIONS
10.1 Ethical Approval
10.2. Informed Consent Process
10.3. Adverse Events
10.4 Sponsorship and Indemnity
10.5. Data Protection and Confidentiality
10.6. Trial Closure

13. REFERENCES

14. APPENDICES
i. Site Survey
ii. Surgeon Survey
iii. Surgeon Information Sheet
iv. Patient Information Sheet (PIS)
v. Case Report Form (CRF)

For peer review only - http://bmjopen.bmj.com/site/about/guidelines.xhtml

vi. Informed Consent Form (ICF)
vii. Breast-Q Questionnaire
viii. Screening Log
ix. NWBRC 'TIC' List
x. Topic Guides for Surgeon Interviews (1,2, and 3)
xi. Local Site Proposed Patient Identifiable Data Sheet
xii. Schedule of Insurance
xiii. HRA Statement of activity
xiv. HRA Schedule of activities

For peer review only

For peer review only - http://bmjopen.bmj.com/site/about/guidelines.xhtml

1. PROTOCOL SUMMARY

Title: A mixed methods IDEAL 2a/2b prospective cohort study to determine the safety and effectiveness of pre-pectoral implant based breast reconstruction

Phase: IDEAL Phase 2a/2b

Sample Size: 341 patients

Inclusion criteria: Female patients aged 16 or over electing to undergo immediate implant based breast reconstruction for breast cancer or risk reduction

Exclusion Criteria: Patients undergoing revisional or delayed breast reconstruction

Number of Sites: 30-40 UK sites

Study Duration: Recruitment: 18 months

Intervention: Pre-pectoral breast reconstruction using biological or synthetic mesh +/- dermal sling

Primary Outcome: Evaluate the **safety** of pre-pectoral breast reconstruction as a technique; defined as **implant loss rate at 3 months <10%**

Secondary Outcomes:

1. To evaluate other key safety outcomes for pre-pectoral breast reconstruction including infection; return to theatre and readmission at 3 months compared with published national audit data
2. To determine the feasibility of using mixed methods to promote shared learning and identify when the technique is sufficiently stable for evaluation in the context of an RCT
3. To explore the feasibility, design and conduct of a future RCT by exploring the numbers of patients potentially eligible for pre-pectoral reconstruction; the types and frequencies of products used and approaches to concomitant interventions
4. To build capacity to deliver a future RCT

For peer review only - http://bmjopen.bmj.com/site/about/guidelines.xhtml

1.1. Schematic of Study Design

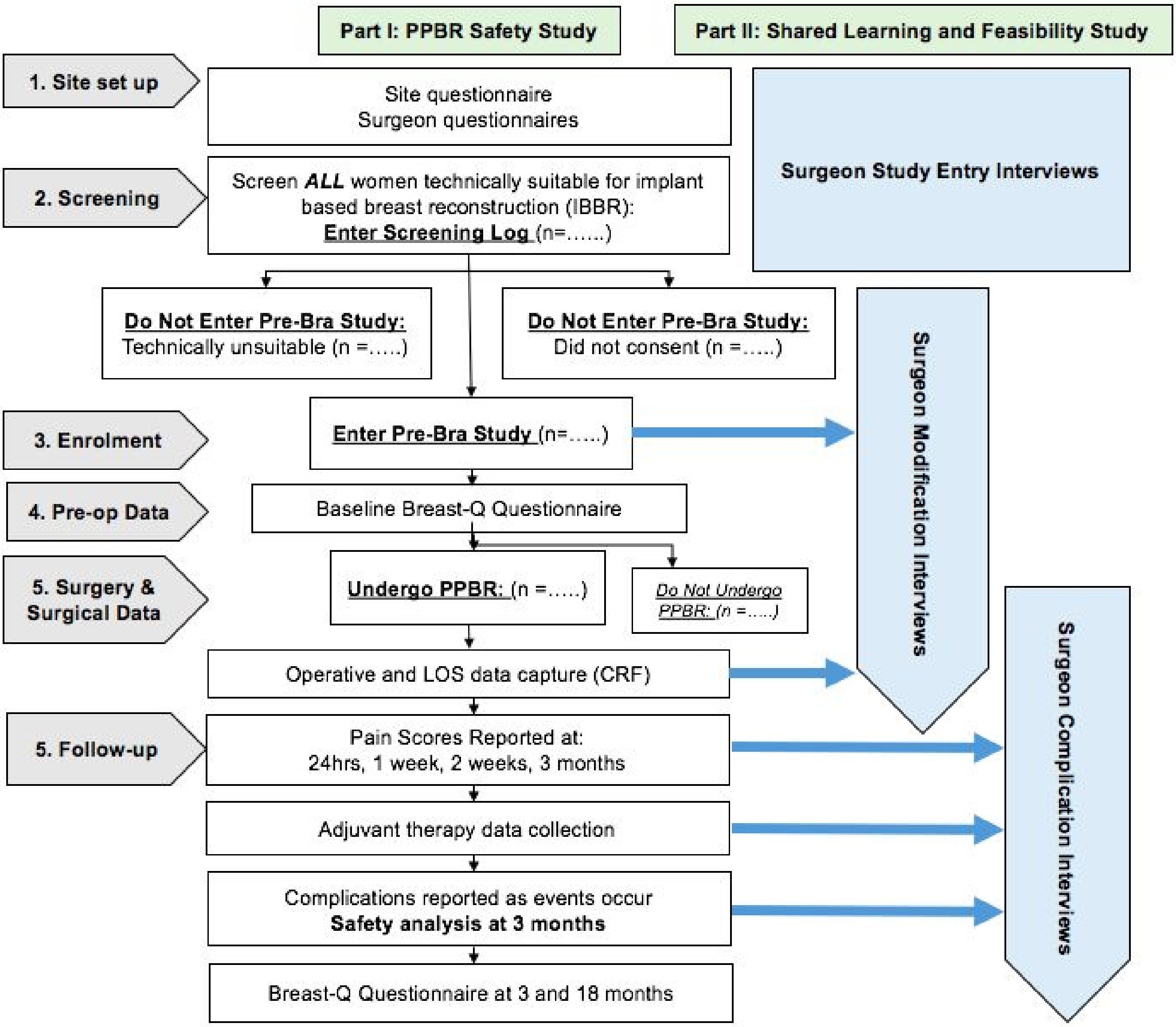

For peer review only - http://bmjopen.bmj.com/site/about/guidelines.xhtml

2. DEFINITIONS

Implant loss - any unplanned removal of the implant without replacement of the prosthesis (implant or expander) for infection, wound problems or other indication within the first 3 months following surgery.

Implant salvage – return to theatre for debridement of wound/ re-suturing /drainage of infection/ washout/ or other indication in which the implant is removed and immediately replaced either with the same device or a tissue expander with primary closure of the wound.

Unsuccessful implant salvage (considered equivalent to implant loss) - the implant is no longer in situ 3 months after the salvage procedure was performed.

Infection - A hot, red swollen breast associated with one of the following; a temperature, pus at the wound site, a raised white cell count and/or; a positive wound swab within the first 3 months following surgery. This will be further classified as:

- Minor – requiring oral antibiotics only;
- Major 1 – requiring admission for IV antibiotics and/or debridement;
- Major 2 – requiring surgical drainage/debridement

Readmission to hospital – any re-admission to hospital in the 3 months following surgery directly related to the procedure (e.g. with infection requiring antibiotics).

Return to theatre – Return to the operating theatre at any time during the first 3 months following surgery to deal with any complication of the reconstruction. This will not include any secondary oncological procedures such as axillary clearance or planned procedures including exchange of expander for a fixed volume implant or lipo-modelling.

Seroma - A symptomatic collection of fluid around the reconstructed breast following surgery requiring aspiration. The total number of aspirations will be collected at 3 months.

Haematoma - A collection of blood in the reconstructed breast

- Minor – managed conservatively or by aspiration in clinic;
- Major – requiring surgical evacuation

Mastectomy skin flap necrosis - Any area of skin loss on the reconstructed breast

- Minor – managed conservatively with dressings;
- Major 1 – requiring debridement (in clinic or theatre) under local anaesthetic (LA);
- Major 2 – requiring surgical debridement under general anaesthesia (GA)

Nipple necrosis – Any area of necrosis of the nipple areolar complex (NAC) (if nipple preserving mastectomy)

For peer review only - http://bmjopen.bmj.com/site/about/guidelines.xhtml

- Minor – managed conservatively with dressings;
- Major 1 – requiring surgical debridement under LA in clinic or theatre;
- Major 2 – requiring surgical debridement under GA in theatre

Wound dehiscence – separation of the skin edges at the wound site.

- Minor – treated conservatively;
- Major – requiring return to theatre for re-suturing under GA

Displaced implant requiring repositioning under GA – any implant displacement that requires surgical correction to restore its position

In hospital complication – any complication that occurs during the patient's initial hospital stay at the time of their reconstructive surgery. This includes systematic complications such as DVT/PE and procedure specific complications such as haematoma.

Major complication - Any complication requiring readmission to hospital or return to theatre

Minor complication - Any other complication
Surgeons will be asked to report any learning arising from complications and whether they would be willing to be contacted to discuss this in more detail.

For peer review only - http://bmjopen.bmj.com/site/about/guidelines.xhtml

3. BACKGROUND

55,000 women per year in the UK are diagnosed with breast cancer [1]. 40% will require a mastectomy [2] and of those who choose breast reconstruction, the majority elect to have an implant-based procedure [3]. Sub-pectoral implant-based breast reconstruction with mesh has become the standard of care for immediate implant-based breast reconstruction (IBBR) in the UK despite a lack of high-quality evidence to support the safety or effectiveness of the technique [4].

Implant-based reconstruction is rapidly evolving and recently, mesh-based techniques have evolved further with early studies [5] suggesting good results when fixed-volume implants are completely covered in a biological [6-8] or synthetic mesh [9-12] and placed in a pre-pectoral position. This ‘muscle-sparing’ technique may result in less post-operative pain, more natural results and may prevent ‘implant animation’, the upwards movement of the implant seen when the pectoral muscle contracts.

While early results [13] are promising in the hands of a few expert surgeons [6, 7, 9-11, 14, 15], historically subcutaneous implant placement without mesh, was abandoned by the reconstructive community due to high complication rates [16, 17]. There is therefore a need to robustly evaluate pre-pectoral implant reconstruction (PPBR) before it becomes standard practice [13]. A recent large-scale audit of UK practice, The iBRA Study, published outcomes at 3 months for over 2000 breast reconstructions [18]. Implant loss rates at 3 months were 8.9%, but this data-set was comprised mostly of sub-pectoral reconstructions with few (2%) pre-pectoral cases. Randomised clinical trials (RCTs) provide the best evidence, but the safety of PPBR has yet to be established; its indications are still evolving and it has not yet been adopted by a sufficient number of surgeons for a randomised trial to be feasible. Preliminary work is therefore needed before definitive evaluation can be considered.

The IDEAL (Idea-Development-Exploration-Assessment-Long-term Study) Framework provides recommendations for the evaluation of a surgical innovation from first-in-man to long-term study [19, 20]. Phases 2a (Development)/2b (Exploration) focus on feasibility work for a definitive future trial including establishing the risks and benefits of the technique; the stability of the intervention, its indications and the adoption of the procedure by a wider group of surgeons from the initial innovators [21]. Whilst the scope and intention of IDEAL is clearly defined the system is not well operationalised, and methods for the optimal conduct of early phase surgical studies are unclear. Embedding qualitative methods within an IDEAL study may provide a novel way of enhancing shared learning within the surgical community to reduce learning curve effects and allow a new procedure to be introduced more safely and efficiently. Monitoring and sharing modifications and group learning may also provide effective way of determining when the procedure is sufficiently stable for formal evaluation in the context of a definitive RCT.

For peer review only - http://bmjopen.bmj.com/site/about/guidelines.xhtml

4. AIMS AND OBJECTIVES

The overall aim of this study is to determine the feasibility of using mixed methods within an IDEAL 2a/2b study to explore the short-term safety and effectiveness of pre-pectoral breast reconstruction and determine when it is sufficiently stable for formal evaluation in an RCT.

4.1. Specific Objectives

1. Safety and effectiveness of pre-pectoral breast reconstruction

i. To establish the short-term safety (implant loss; infection; re-operation and readmission at 3 months) of pre-pectoral breast reconstruction compared with published audit standards [18]

ii. To determine post-operative pain scores at 24 hours, 1 week, 2 weeks and 3 months

iii. To explore patient reported outcomes at 3 and 18 months using the BREAST-Q

2. Feasibility of using novel mixed methods to promote group learning and identify intervention stability in an IDEAL 2a/2b study

iv. To determine the acceptability and feasibility of using qualitative methods to promote shared learning by comparing the total number of surgeons participating in the study; the number completing the free-text boxes within the case report forms (CRFs) and the numbers participating in brief semi-structured interviews.

v. To explore the most effective methods of providing feedback to disseminate best practice.

vi. To determine the feasibility of using qualitative methods to determine intervention stability by evaluating the number and content of surgeon reported modifications to technique and patient selection criteria over time.

3. Inform the design and conduct of a future RCT

vii. To determine the numbers of patients undergoing IBBR at participating centres; the numbers considered technically suitable for pre-pectoral reconstruction and the numbers consenting to undergo the procedures (using screening log data to inform sample size).

viii. To explore the products used and proportions of patients receiving each product to inform the selection of comparators in the main trial.

ix. To explore use of concomitant interventions (e.g. drains, antibiotics) to inform the degree of pragmatism acceptable in the main trial.

x. To explore the feasibility of using electronic patient self-report to inform the use of e-PROMS in the main study.

xi. To determine consensus regarding the feasibility, design and conduct of a future RCT.

4. Building capacity to deliver a future RCT

For peer review only - http://bmjopen.bmj.com/site/about/guidelines.xhtml

xii. To establish a network of surgeons engaged in the need for evaluation who are able to perform pre-pectoral breast reconstruction to a high standard and who will participate in a future study.

4.2. Part I: The Pre-Pectoral Reconstruction Safety Study

The primary aim of The Pre-Bra Study is to assess safety.

Therefore, Objective 1 will be addressed within the Pre-Pectoral Reconstruction Safety Study. The safety study aspect of Pre-Bra will measure quantitative clinical outcomes using the methods outlined below.

4.3. Part II: The Shared Learning Feasibility Study

Objectives 2, 3 and 4 will be addressed within a Shared Learning and Intervention Stability Feasibility Study. This aspect of the Pre-Bra study will use mixed quantitative and qualitative data, collected via semi-structured interviews with participating surgeons and free text responses entered into individual patient case report forms (CRFs).

4.4. Study Endpoints

Pre-Pectoral Reconstruction Safety Study Endpoints

The primary outcome for the pre-pectoral safety study will be implant loss at 3 months defined as removal of the implant or expander without replacement as a result of complications (see section 2. 'Definitions').

Secondary safety outcomes will be:

- Infection, re-operation of complications and readmission at 3 months
- Major complications defined as any complication that requires re-operation or readmission at 3 months.
- Patient reported pain at 24 hours; 1 week 2 weeks and 3 months.
- Patient satisfaction at 3 and 18 months using validated BREAST-Q breast reconstruction questionnaire.

Shared Learning Feasibility Study Outcomes

The feasibility of using the combination of free text reporting within the CRF and brief semi-structured interviews will be assessed by comparing:

The number of surgeons recruiting to the study with:

At study entry

For peer review only - http://bmjopen.bmj.com/site/about/guidelines.xhtml

The number of surgeons completing the surgeon questionnaire; the number of surgeons consenting to be contacted for interview and the number of surgeons interviewed

For modification and complication learning

The numbers of cases for which i) modifications or ii) complications are reported via the CRFs; the proportion of these cases for which details are provided; the proportion of these cases in which the surgeon consent to be contacted for interview and the number who are interviewed.

The themes emerging from the interviews will be reviewed by the study team. Any common themes emerging from modification and complication interviews will be shared with participating surgeons using a combination of methods. These will include a 'tips and tricks' section on the study website (www.ibranet.co.uk); weekly email updates; study newsletters and 'YouTube' videos demonstrating best practice.

The study steering group (SSG) will hold a consensus meeting at the end of the study to agree best practice and the final design for the main study if this is considered feasible.

5. METHODS

5.1. Overall Study design

The Pre-Bra Feasibility Study is a multicentre, IDEAL Phase 2a/2b prospective observational cohort study with embedded qualitative methods. The study comprises two component parts; one assessing safety of the technique of PPBR and one evaluating the feasibility of sharing learning and building capacity for a future RCT. The two aspects of the study are integrated and inform each other.

5.2. Surgeon Participants

Surgeon Inclusion Criteria

All UK breast and plastic surgical centres currently performing pre-pectoral breast reconstruction using any technique will be invited to participate via professional associations (Association of Breast Surgery [ABS], British Association of Plastic Reconstructive and Aesthetic Surgeons [BAPRAS] and the Royal College of Surgeons of England [RCSE]).

Surgeon Exclusion Criteria

There are no exclusion criteria precluding consultant surgeons currently performing PPBR from participating in the study. However, surgeons wishing to start performing

For peer review only - http://bmjopen.bmj.com/site/about/guidelines.xhtml

PPBR are encouraged to follow the ABS/BAPARS guidelines [22] prior to entering patients in the study.

Surgeon Recruitment and Consent

Interested clinicians will be asked to nominate a principal investigator (PI) at their site to complete a short survey by following a link to the REDCap secure online database. Individual surgeons who wish to recruit patients to the study will be asked to complete a brief online survey about their practice before beginning recruitment. (See Appendix i. Site Survey and Appendix ii. Surgeon Survey)

Surgeons interested in taking part in The Pre-Bra Study will be offered an information sheet (See Appendix iii. Surgeon Information Sheet) outlining their role and responsibilities. Surgeons who consent to take part in qualitative interviews will be audio recorded, verbal consent will be recorded before interviews commence.

5.3. Patient Participants

Patient Inclusion Criteria

Consecutive women >16 who require a mastectomy for breast cancer or risk-reduction who:

a. Elect to undergo immediate implant based breast reconstruction.
b. Are considered suitable for PPBR by their surgeon.
c. Understand and accept that PBRR is innovative and that outcome data are limited.
d. Accept to take part in the study.

Patient Exclusion Criteria

These may be technique or mesh-specific and will be iteratively modified by the Steering Group (SG) as the study progresses. To allow for the scope of practice within the study, absolute and relative exclusion criteria to study entry have been proposed.

- **Absolute exclusion criteria** should be applied to all patients, irrespective of surgical experience or other factors.
- **Relative exclusion criteria** can be applied flexibly according to the surgeon's experience with the procedure in conjunction with the patient's consent following discussion that the risk of complications may be increased in this setting.

Proposed **absolute exclusion criteria** are:

a. Patient unwilling to give consent and participate in the study.

For peer review only - http://bmjopen.bmj.com/site/about/guidelines.xhtml

b. Thin/ insufficiently vascularised skin-flaps as assessed by the operating surgeon.
c. Revision /delayed breast reconstruction.

For surgeons in the learning phase or early practice of PPBR, it is recommended that the following additional proposed **relative exclusion criteria** are applied in line with the ABS/BAPRAS guidelines for mesh-assisted breast reconstruction: [22]

d. Smokers or ex-smokers <6 weeks
e. Previous radiotherapy to the ipsilateral breast/chest wall
f. BMI >30 (except for dermal sling procedures)
g. Implant size >600cc
h. Anticipated post mastectomy radiotherapy
i. Poorly controlled diabetes

Both absolute and relative exclusion criteria may be modified by the SSG during the study.

Patient Screening

All female patients >16 undergoing mastectomy for breast cancer or risk reduction who elect to undergo immediate implant-based breast reconstruction are eligible for inclusion in the study.

Potential participants will be identified from clinics and multidisciplinary meetings by the clinical and research teams.

Patients will be reviewed by their operating surgeon to determine whether they are suitable for implant-based breast reconstruction and whether they may be suitable for pre-pectoral techniques.

All patients electing to undergo immediate implant-based breast reconstruction should be entered onto the study screening log to determine the proportion of patients who:

i. Are considered technically suitable for pre-pectoral technique by the operating surgeon.
ii. For those patients who are considered not suitable for PPBR – reasons for this this (e.g. relative exclusion criteria: smoker; previous radiotherapy etc. or other reason).
iii. For patients considered technically suitable for PPBR, the proportion who accept the technique.
iv. For those patients accepting PPBR, the proportion who consent to study participation

This will inform the feasibility of a future trial.

Patient Recruitment and Consent

For peer review only - http://bmjopen.bmj.com/site/about/guidelines.xhtml

Patients considered suitable for pre-pectoral reconstruction and who elect to undergo the procedure will be given a study information leaflet at the time they have made a decision to have reconstructive surgery (see Appendix iv. Patient Information Sheet (PIS). They will be seen at a follow up appointment by a member of the clinical or research team as per local practice to discuss the study, if needed and to sign a consent form for study participation (see Appendix vi. Informed Consent Form (ICF).

Patients will be allocated a study ID number.

Patients will be asked to consent to the collection of anonymised clinical data about their care, this could be kept for up to 10 years after the close of the study in order to support future research studies. They will also be specifically asked about the collection and storage of identifiable data. The central study team will request access to each patient's name, date of birth, phone number, address and NHS number in order to continue to follow them up after the study closes. This could be via their electronic record using data-linkage studies, or patients may consent to being contacted in future to complete further questionnaires to assess long-term satisfaction with PPBR.

Patients will be given a date for surgery as per unit protocol and undergo pre-operative assessment. They will be required to complete baseline patient reported outcome questionnaires (see Appendix vii. Breast-Q Questionnaire).

6. DATA COLLECTION PART I: Pre-Pectoral Reconstruction Safety Study

6.1. Pre-Pectoral Breast Reconstruction

All patients in whom pre-pectoral reconstruction with any technique or product is planned will be eligible for inclusion in the study.

Eligible Mesh and ADM Products

Included products will include:

- Artia
- BRAXON
- Fortiva
- MESO
- Strattice
- Surgimend
- TiLOOP
- TIGR
- Cellis
- Complete dermal sling
- Hybrid dermal sling- with mesh

For peer review only - http://bmjopen.bmj.com/site/about/guidelines.xhtml

All products have been licensed for use in the UK and are CE marked. New products will be reviewed for study inclusion on a case by case basis by the Study Steering Group (SSG).

Standardisation of Surgical Technique

All patients will undergo a skin or nipple preserving mastectomy followed by an immediate implant-based breast reconstruction.

Participating surgeons will undertake the procedure as per their standard practice. Mesh choice (biological or synthetic and the product used) and implant selection (fixed volume; adjustable implants or tissue expanders) will be as per surgeon preference. All patients will be planned for a pre-pectoral reconstruction prior to surgery.

The following steps of the PPBR procedure will be considered mandatory, prohibited and flexible/discretionary according to the typology described by Blencowe et al [23]**.**

- **Mandatory** – Insertion of a tissue expander/adjustable implant or fixed volume implant
- **Prohibited** –Raising the pectoralis muscle)
- **Flexible/discretionary** – all other steps of the procedure (see table below

If the surgeon considers that pre-pectoral reconstruction is not possible at the time of surgery or modifies the planned procedure (e.g. inserts tissue expander rather than fixed volume implant), this will be recorded on the CRF with reasons for the modification (e.g. thin skin flaps). Details of the alternative procedure performed (e.g. sub-pectoral reconstruction) will be collected. Surgeons will also be encouraged to record any learning arising from the case.

Strategies to minimise infection (e.g. use of laminar flow, cavity irrigation, glove change) will be as per local practice but participating centres will be encouraged to adhere to published best practice and use the evidence-based Manchester "TIC List" wherever possible [24].

Use of drains and other concomitant interventions will be permitted as per local practice. Data regarding variation in practice between centres in operative procedure and concomitant interventions (e.g. drains and antibiotics) will be collected using a surgeon questionnaire at the start of the study (see appendix ii. Surgeon Survey)

Components of Pre-Pectoral Breast Reconstruction (PPBR) Table 1.

**Key**	
Green	Mandatory step
Amber	Flexible/ discretionary step
Red	Prohibited step

For peer review only - http://bmjopen.bmj.com/site/about/guidelines.xhtml

Component of the intervention	Examples of factors to be considered*
**Before skin incision**	Alcoholic skin prep
	Laminar flow
	Antibiotics
	Drapes
	Surgeons double-gloved
	Strategies to minimise theatre traffic (I.e. door signage)
	Patient warming technique (under patient/bear-hugger/other)
**Incisions and access**	Position of skin incision on breast (IMF, lateral, removing NAC, wise pattern etc.)
	Single or separate incisions for axillary procedures (if applicable)
	Whether space between axilla and breast is closed (if applicable)
	Nature of axillary procedure (SLNB vs ANC vs none)
**Dissection/resection**	Type of mastectomy (skin/nipple-sparing/skin reducing)
	Use of hydro dissection (saline/LA +/- adrenaline)
	Method of dissection of the skin flaps (scissors/monopolar diathermy; bipolar scissors/knife/plasma blade)
	Surgeon assessment of adequacy of skin flaps including use of SPY technology
	Raising the pectoralis major or serratus anterior muscle
**Haemostasis**	Use of wash (saline/water/none)
	Request for representative blood pressure (Y/N)
**Reconstruction**	Planned 1 or 2 stage
	Tissue expander/adjustable implant (e.g. Becker)/fixed volume implant + volume relative to mastectomy weight/volume
	Full wrap vs anterior wrap
	Product used
	Number and position of sutures (if any) to the pectoralis fascia and suture used
	Strategies to minimise infection – glove change/cavity irrigation (water/saline/antiseptic/antibiotics etc.)/ re-prep and drape/use of Ioban or Tegaderm dressing on wound prior to implant insertion/Keller or plastic funnel use/wash of implant in antibiotics/antibiotic sponge etc.)
**Closure**	Number of layers
**After skin closure**	Type of dressing (skin glue/occlusive dressings/PICO etc.)
	Pressure dressings
	Support e.g. sports bra

For peer review only - http://bmjopen.bmj.com/site/about/guidelines.xhtml

**Insertion of surgical adjuvants**	Number of drains and duration of drainage
	Tunnelling of drains (if used)
**Other**	Use of post-operative antibiotics

6.2. Case Report Forms

The pre-pectoral reconstruction safety aspect of the study is designed to assess safety and effectiveness of pre-pectoral breast reconstruction.

Demographic, comorbidity and operative data will be collected for each participant via a standardised electronic Case Report Form (see Appendix v. CRF) which will be directly uploaded onto the REDCap secure online database.

Complications and oncological data will be collected at 30 days and 3 months by clinical or case note review according to local follow up policies. No additional clinic visits will be required for the study. All complications are defined a priori (See section 2. 'Definitions')

Assessing Complications

Post-operative complications will be assessed clinically at hospital follow-up appointments according to each sites usual schedule i.e. routine standard of care. Post-operative complications affecting each patient will be recorded by their local clinical research team as the events occur. At 3 months the status of each patient regarding complications will be evaluated to determine the initial safety of the technique. The primary outcome by which safety is assessed will be implant loss at 3 months. (Implant loss is defined in Section 2. 'Definitions')

The remaining 3 relevant outcomes from the iBRA Study [18] will also be reviewed to determine the safety of PPBR. These are:

- Infection at 3 months
- Unplanned re-operation for complications relating to the implant reconstruction at 3 months
- Readmission to hospital for complications related to the implant reconstruction at 3 months

Other clinically relevant complications will also be assessed within the first 3 months after surgery including:
(All of the following terms are defined in section 2. 'Definitions')

- Seroma
- Haematoma (minor or major)
- Mastectomy skin flap necrosis (minor, major 1 or major 2)
- Nipple necrosis (minor, major 1 or major 2)
- Wound dehiscence (minor or major)
- Displaced implant requiring repositioning under GA

For peer review only - http://bmjopen.bmj.com/site/about/guidelines.xhtml

- In hospital complication
- Major complication
  Minor complication

Schedule of Trial Procedures (PPBR Safety Study)

It is anticipated that patients entering the study will be followed up for 18 months.

Screening (Using SEAR method)
Screening data will not include any patient identifiable information:

- All patients electing to undergo IBBR
- Number technically suitable for PPBR
- Number not considered technically suitable for PPBR and reasons for this
- Number accepting PPBR
- Number accepting study participation

Baseline Pre-Operative Assessment

- Patient demographic data captured
- BREAST-Q completed (Reconstruction-pre-operative version)

Surgical Admission

- PPBR procedure: operative data captured
- Length of post-operative hospital stay
- Post-operative pain at 24 hours or point of discharge
- Any immediate in-hospital complications

Early clinical follow-up

- Post-operative pain score at 1 and 2 weeks electronically self-reported by the patient if no longer in hospital
- Assessment and recording of complications at clinical review: timing as per each site's routine standard of care follow-up arrangements
- Oncological data and planned adjuvant treatment (if appropriate)

3-month Data

- Assessment of complications including re-admission and re-operation if these events occur
- Information taken from clinical notes and in discussion with each site's research team
- Pain Scores electronically self-reported by the patient at 3 months
- PROMs: Breast-Q reconstruction – satisfaction with information; patient reported complications

18-month Data

- BREAST-Q completed (Reconstruction-pre-operative version)

For peer review only - http://bmjopen.bmj.com/site/about/guidelines.xhtml

Tabulated Schedule of Trial Procedures (PPBR Safety Study)

**Study Time Point**		**Screening**	**Baseline Assessment**	**Surgical Admission**	**Early Clinical Follow-up**	**3-month data**	**18-month data**
**Data Capture:**							
Assessment of Eligibility Criteria		X	X				
Signed Consent Form			X				
Breast Reconstruction Surgical Data				X			
Patient Demographic data collection		X	X				
Length of Hospital Stay				X			
Patient Oncological and Adjuvant Data					X		
Clinical Examination	Assessment of suitability for PPBR	X	X	X			
	Assessment for complications			X	X	X	
PROMs	Breast-Q		X			X	X
	Post-operative Pain Scores			X	X	X	

6.3. Patient reported outcomes measures (PROMs)

Shoulder function; physical well-being and satisfaction

As the hypothesised benefits of PPBR include less post-operative pain and less impact on arm and shoulder function in addition to improved cosmetic outcome and patient satisfaction, these key patient reported outcomes will be assessed using validated PROMs.

Patients will complete the Breast-Q Reconstruction (Pre-operative) (satisfaction with breasts; psychosocial well-being; physical well-being (including arm/shoulder function) and sexual well-being modules) prior to surgery.

The Breast-Q Reconstruction (post-operative) questionnaire (satisfaction with information, satisfaction with breasts, satisfaction with breasts (implant); psychosocial well-being, physical well-being; satisfaction with outcome; satisfaction with nipple (if appropriate); sexual well-being) will be completed at 3 and 18 months.

Pain

Post-operative pain will be assessed on a visual analogue scale (VAS) from 1 to 10 at 24 hours post-operatively or prior to discharge if the patient is a day-case and 1 and 2-weeks

For peer review only - http://bmjopen.bmj.com/site/about/guidelines.xhtml

For peer review only

and 3 months' post-surgery. These scores will be patient self-reported electronically. Telephone or E-mail reminders will be initiated after 1 month if no response is received.

Pain will also be assessed using items contained within the Breast-Q Physical Well-being Chest and Upper body subscale pre-operatively and at 3 and 18 months post-operatively.

6.4. Protocol Deviations

As detailed above, surgeons will be asked to report the following via the CRF:

a. Patients in whom PPBR was planned but had to be abandoned completely (i.e. another form of reconstruction or no reconstruction performed)
   i. Reasons why pre-pectoral approach was abandoned
   ii. Procedure performed instead
   iii. Any reflections or learning regarding the case
b. Patients in whom the planned procedure was modified (but pre-pectoral reconstruction still performed)
   i. Details of the modification made (e.g. insertion of tissue expander rather than fixed volume implant)
   ii. Reasons that the modification was made (e.g. thin skin flaps)
   iii. Any reflections or learning regarding the case

This data will be collected via free-text responses in the CRF. If the surgeon involved consents to a telephone interview, issues arising will be explored in more detail.

7. DATA COLLECTION PART II: Shared Learning Feasibility Study

7.1. Opportunities for Shared Learning

Opportunities for identifying shared learning, best practice and addressing intervention stability between participating surgeons will occur at the following points in the study:

I. Study entry
II. Following procedure modifications
III. Following complications

Surgeons electing to participate in the study will be sent an information sheet which will outline the proposed shared learning study (see appendix iii. Surgeon Information Sheet) and what participation may involve. Surgeons will be able to recruit patients to The Pre-Bra Study without participating in the shared learning part of the study but full participation will be encouraged by the Study Steering Group (SSG).

At each time point a combination of free text responses on standardised case report forms (CRFs) combined with semi-structured qualitative interviews will be used to explore group learning as detailed below.

For peer review only - http://bmjopen.bmj.com/site/about/guidelines.xhtml

7.2. Study Entry

Surgeon questionnaire

Prior to commencing patient recruitment, participating surgeons will be asked to complete a surgeon questionnaire. The questionnaire (see appendix ii. Surgeon Survey) will explore techniques and products used; experience; approaches to concomitant interventions and patient selection criteria. The questionnaire will include consent to be contacted regarding a brief (20 min) semi-structured qualitative interview exploring their practice in more detail.

Sampling and data collection

A purposive sample of surgeons consenting to interview will be selected based on techniques and products used; experience; geographical location and differing approaches to concomitant interventions and patient selection.

Semi-structured study entry interviews

Interviews will be organised at a time convenient to the participating surgeons. All interviews will be conducted by telephone using a semi-structured topic guide developed based on the literature and clinical expertise to explore issues surrounding current practice and rationale for this (see appendix x. Topic guides for surgeon interviews). All interviews will be audio-recorded and transcribed in full. Interview participants will be asked to give verbal consent to study participation and audio-recording prior to commencing the interview. The topic guide will be modified iteratively as the study progresses to allow new themes to emerge. Sampling, data collection and analysis will be conducted concurrently and iteratively until data saturation is achieved and no new themes emerge from the data.

7.3. Following Selection Criteria or Procedure Modifications

Modification Case Report Forms (CRFs)

Surgeons will be asked to report whether a modification to the planned procedure was made and if so, to provide details in appropriate free text response boxes on the electronic CRF.

a. Patients in whom PBR was planned but had to be abandoned completely (i.e. another form of reconstruction or no reconstruction performed)
   i. Reasons why pre-pectoral approach was abandoned
   ii. Procedure performed instead
   iii. Any reflections or learning regarding the case
b. Patients in whom the planned procedure was modified (but pre-pectoral reconstruction still performed)
   i. Details of the modification made (e.g. insertion of tissue expander rather than fixed volume implant)
   ii. Reasons that the modification was made (e.g. thin skin flaps)
   iii. Any reflections or learning regarding the case

For peer review only - http://bmjopen.bmj.com/site/about/guidelines.xhtml

Surgeons reporting modifications will also be asked to consent to be contacted to participant on a brief telephone interviews to explore the modification; rationale and outcome in more detail.

Sampling and Data Collection

All surgeons reporting a modification to their patient selection criteria, or their operative technique and providing consent will be contacted to arrange a brief semi-structured qualitative interview.

Semi-Structured Procedure Modification Interviews

All interviews will be conducted by telephone and will focus specifically on the modification; rationale and outcome to minimise the burden on participants. It is anticipated that interviews will take no longer than 20 minutes. Interviews will be conducted using a topic guide developed based on the literature and expert input from the steering group and iteratively modified as the study progresses to allow emerging themes to be explored (see appendix x. Topic guides for surgeon interviews). All interviews will be audio-recorded and transcribed in full. Verbal consent will be obtained for audio-recording and study participation prior to commencing the interviews.

The feasibility of this approach is unknown so initially, it is planned that all surgeons reporting procedure modifications and providing consent will be interviewed. As the study progresses, depending on the number and content of the CRF reports, all consenting surgeons may continue be interviewed or, it may be necessary to purposively sample consenting surgeons based on the details of the modification/rationale/learning reported in the CRF to explore new and emerging themes and allow the details of the modifications to be explored.

Sampling, data collection and analysis will be conducted concurrently and iteratively until saturation is achieved and no new modifications or learning emerge from the data or no further modifications are reported depending on the success of the methodology.

7.4. Following Complications

Complication Case Report Forms (CRFs)

All complications occurring at 3 months will be recorded in individual patient's CRF (see appendix v. Case Report Form).

If complications occur, surgeons will be asked to reflect and comment ("yes/no") as to whether they felt that there had been any learning as a result of the complication either in terms of the surgical technique or patient selection. If surgeons indicate learning, they will be asked to complete a free text response box providing details of the learning. Surgeons

For peer review only - http://bmjopen.bmj.com/site/about/guidelines.xhtml

will also be asked to provide consent to be contacted to participate in a brief telephone interview to discuss the reported learning in more detail.

Sampling and Data Collection

Initially, as with the modification interviews, all surgeons reporting learning arising from complications who provide consent will be contacted for interview. As the study progresses, depending on the number and content of CRF reports, all consenting surgeons may continue be interviewed or it may be necessary to purposively sample consenting surgeons based on the details of the complication/resultant learning reported in the CRF to explore new and emerging themes and allow the details of the learning to be explored.

Semi-Structured Complication Interviews

The complication interviews will be conducted in a similar way to the modification interviews using pre-designed Topic Guides which will be modified iteratively as the study progresses (see section 7.3. 'Phase 3' above).
(See appendix x. Topic guides for surgeon interviews).

8. ANALYSIS PART I: Pre-Pectoral Reconstruction Safety Study

8.1. Sample Size

Sample size calculations are based on the primary outcome of implant loss rate at 3 months. It is expected that the implant loss rate will be 9% or lower based on findings from the recent iBRA study [18] 15% or greater will be deemed unacceptably high.

A sample size of 310 patients would result in a two-sided 95.0% confidence interval for a single proportion, assumed to be 0.10, with a width equal to 0.07. Allowing for a 10% loss to follow-up at 3 months at least 341 patients will be recruited to inform a future trial with implant loss as the primary outcome.

Based on data from The iBRA study, the number of pre-pectoral reconstructions performed at each site is likely to be small (4-40 per year). We will therefore aim to recruit from between 30 and 40 sites over a period of 18 months.

8.2. Analytical Methods

Data Summaries

Simple summary statistics will be calculated to describe demographic, procedure, process and outcome data. Categorical data will be summarised by counts and percentages and continuous data by median, interquartile range and range.

For peer review only - http://bmjopen.bmj.com/site/about/guidelines.xhtml

We will establish the proportion and 95% confidence intervals of patients experiencing implant loss and other key safety outcomes (re-admission for complications, re-operation and infection) at 3 months and compare these with findings with those reported in the iBRA study [18].

Patient Groups for Analysis

An exploratory risk factor analysis will be conducted to explore the impact of type of mesh and known risk factors for complications including smoking; BMI, radiotherapy if sufficient numbers of patients are recruited to the study.

Missing Data

It is anticipated that missing data will be minimal. Allowing for a 10% loss to follow-up at 3 months at least 341 patients will be recruited to inform a future trial with implant loss as the primary outcome.

If study records are incomplete at 3 months, consent will be obtained for clinical note review to collect data regarding complications, re-admissions or re-operations. The central Pre-Bra study team will be responsible for issuing polite reminders when patient–reported data is not electronically entered. Patients will be followed-up at 18 months post-operatively with a Breast-Q validated questionnaire. Participants will be given the option to decline to enter any further data by withdrawing from the study at any point.

8.3. Interim Analysis and Monitoring

A planned interim analysis will be performed when 155 patients have been recruited to the study and followed-up for a minimum of 3 months. The report will be reviewed by the study steering group (SSG) and the results will be made available to the study sponsor.

If the overall implant loss rate at 3 months following surgery is 15% or more, the study will be stopped.

8. ANALYSIS PART II: Shared Learning Feasibility Study

9.1. Analysis of Results

Analysis of the free text responses will be an ongoing and iterative process commencing soon after the study begins and will inform sampling for the semi-structured interview phase of the study. Text will be assigned codes and analysed using the constant comparative technique of grounded theory. A combination of thematic and content analysis will be used to identify common themes. These will inform the topic guide for the interviews and the feedback provided to study participants.

For peer review only - http://bmjopen.bmj.com/site/about/guidelines.xhtml

All interviews will be transcribed verbatim and analysis will commence soon after data collection commences. Transcripts will be systematically assigned codes using the qualitative data analysis package NVivo, and analysed thematically using the constant comparison technique of grounded theory to develop a coding framework which will be developed during the analysis.

The themes identified from the CRFs and the interviews will be used to identify potential areas of learning and best practice to be explored in future interviews and fed back to study participants. Data collection will continue until saturation is achieved and no new themes emerge or no further modifications or learning from complications are reported.

9.2. Distribution of Shared Learning

Themes relating to technical modifications, complications and learning will be identified from the CRFs and subsequent surgeon interviews. Valuable technical "tips and tricks" will be collated and distributed via email newsletters, the study website (www.ibranet.co.uk) and social media channels. It is hoped that these outlets will have interactive content and become a valuable resource for the community of surgeons taking part in the study.

10. PUBLICATION

The results from different centres will be analysed together and published as soon as possible. Individual clinicians will undertake not to submit any part of their individual data for publication without the prior consent of the study steering group (SSG).

The Trial Steering Group will form the basis of the Writing Committee and advise on the nature of publications. The Uniform Requirements for Manuscripts Submitted to Biomedical Journals (http://www.icmje.org/) will be respected.

11. STUDY OVERSIGHT

11.1. Study Steering Group (SG)

The study steering group (SSG) will be formed comprising the Chief Investigator and other lead investigators (clinical and non-clinical). The SSG will be responsible for the day-to-day running and management of the trial and will meet approximately 3 times a year.

Trial oversight will be conducted by the SSG, which will meet regularly during the study. An interim data analysis will be performed assessing the study's primary outcome of implant loss rate when 155 patients have been recruited and passed the 3-month follow-up point. Data collected subsequently will be reviewed on a regular basis by the SSG.

For peer review only - http://bmjopen.bmj.com/site/about/guidelines.xhtml

12. STUDY ADMINISTRATION AND ETHICAL ISSUES

12.1. Ethical Approval

Ethical approval will be sought from the National Research Ethics Service prior to initiation of the study

The trial protocol will be reviewed by the next available Multi-Centre Research Ethics Committee (MREC) and all participating sites will undergo site specific assessment of capacity and capability prior to commencing patient recruitment. A copy of all site approval documents and a copy of the patient information sheet (PIS) and informed consent form (ICF) on local headed paper will be forwarded to the study team before patients are entered. The central study team should receive a confirmation of capacity and capability for each new centre via the site's R&D department.

12.2. Sponsorship and Indemnity

Trial indemnity insurance is provided by the sponsor, The University of Bristol (see appendix xii. Schedule of Insurance document).

12.3. Informed Consent Process

Written informed consent will be obtained from all patients participating in the study. Patients will be informed they can withdraw at any time [see appendix vi. Informed Consent Form (ICF)].

Surgeons will receive a surgeon information sheet and will give verbal informed consent prior to participating in any interviews (see appendix iii. Surgeon Information Sheet).

12.4. Data Protection and Confidentiality

All study participants will be anonymised using unique study identifiers. Personal data will be stored in a separate encrypted file, in a separate location from the study data on the University of Bristol server. It will be accessible to the research team only. All study documentation will be identifiable by study identifiers only. Data will be stored and managed using REDCap software.

At each local NHS site, the research team (clinical staff in conjunction with the LCRN) will collect patient identifiable data for the patients recruited at their site. A template document is supplied to guide local teams for this purpose (see appendix xi. Local Site Proposed Patient Identifiable Data Sheet). This information will be kept in a database alongside the study ID which is used to identify the patient on REDCap secure online database. The two identifiers must be kept alongside each other in case a patient needs to be removed from the central, anonymised REDCap database. For example, if a participant wishes to withdraw or if they

For peer review only - http://bmjopen.bmj.com/site/about/guidelines.xhtml

lose capacity. Each NHS site will require permission form their local Caldecott guardian to store this data and will be responsible for doing so in a physical and electronically secure fashion.

Regarding surgeon-interviews: verbatim quotations that may be used for publications or presentations will be anonymised so that participants are not identifiable.

12.5. Adverse Events

No additional adverse events are anticipated for patients participating in this observational study. (Except those inherent risks of surgery which are independent of the study, and to which the patient must consent in association their surgeon and in accordance with local NHS trust policy.)

12.6. Study Closure

The Pre-Bra Study will close when all data required to satisfactorily address the primary outcome has been collected. At this point at least 341 women will have been recruited and followed-up for a minimum of 3 months.

At the point of trial closure, the central study team and Chief Investigator (CI) will notify the sponsor and all other relevant stakeholders of trial completion.

For peer review only - http://bmjopen.bmj.com/site/about/guidelines.xhtml

13. REFERENCES

1. Cancer Research UK. https://www.cancerresearchuk.org/health-professional/cancer-statistics/statistics-by-cancer-type/breast-cancer#heading-Zero. 2018 18th November 2018].
2. Office for National Statistics, *Long-term Breast Cancer Survival - England and Wales.* 1991-2003, Office for National Statistics,: https://www.ons.gov.uk/peoplepopulationandcommunity/healthandsocialcare/conditionsanddiseases/datasets/cancersurvivalrateslongtermbreastcancersurvivalenglandandwales.
3. Mennie, J.C., et al., *National Trends in Immediate and Delayed Post-Mastectomy Reconstruction Procedures in England: A Seven-Year Population-Based Cohort Study.* European Journal of Surgical Oncology, 2016. **43**: p. 52-61.
4. Potter, S., et al., *Systematic review and critical appraisal of the impact of acellular dermal matrix use on the outcomes of implant-based breast reconstruction.* British Journal of Surgery, 2015. **102**(9): p. 1010-1025.
5. Salibian, A.A., et al., *Subcutaneous Implant-based Breast Reconstruction with Acellular Dermal Matrix/Mesh: A Systematic Review.* Plastic and Reconstructive Surgery – Global Open, 2016. **4**(11): p. e1139.
6. Berna, G., et al., *Evaluation of a novel breast reconstruction technique using the Braxon® acellular dermal matrix: a new muscle-sparing breast reconstruction.* ANZ Journal of Surgery, 2014: p. n/a-n/a.
7. Reitsamer, R. and F. Peintinger, *Prepectoral implant placement and complete coverage with porcine acellular dermal matrix: A new technique for direct-to-implant breast reconstruction after nipple-sparing mastectomy.* Journal of Plastic, Reconstructive & Aesthetic Surgery, 2015. **68**(2): p. 162-167.
8. Zhu, L., et al., *Comparison of subcutaneous versus submuscular expander placement in the first stage of immediate breast reconstruction.* Journal of Plastic, Reconstructive & Aesthetic Surgery, 2016. **69**(4): p. e77-e86.
9. Casella, D., et al., *TiLoop® Bra mesh used for immediate breast reconstruction: comparison of retropectoral and subcutaneous implant placement in a prospective single-institution series.* European Journal of Plastic Surgery, 2014. **37**(11): p. 599-604.
10. Casella, D., et al., *Subcutaneous Tissue Expander Placement with Synthetic Titanium-Coated Mesh in Breast Reconstruction: Long-term Results.* Plastic and Reconstructive Surgery – Global Open, 2015. **3**(12): p. e577.
11. Bernini, M., et al., *Subcutaneous Direct-to-Implant Breast Reconstruction: Surgical, Functional, and Aesthetic Results after Long-Term Follow-Up.* Plastic and Reconstructive Surgery – Global Open, 2015. **3**(12): p. e574.
12. Kobraei, E.M., et al., *Avoiding Breast Animation Deformity with Pectoralis-Sparing Subcutaneous Direct-to-Implant Breast Reconstruction.* Plastic and Reconstructive Surgery – Global Open, 2016. **4**(5): p. e708.
13. Tasoulis, M.K., et al., *Subcutaneous implant breast reconstruction: Time to reconsider?* European Journal of Surgical Oncology (EJSO), 2017.
14. Becker, H., J.G.I. Lind, and E.G. Hopkins, *Immediate Implant-based Prepectoral Breast Reconstruction Using a Vertical Incision.* Plastic and Reconstructive Surgery – Global Open, 2015. **3**(6): p. e412.
15. Vidya, R., et al., *Evaluation of the effectiveness of the prepectoral breast reconstruction with Braxon dermal matrix: First multicenter European report on 100 cases.* The Breast Journal: p. n/a-n/a.
16. SNYDERMAN, R.K. and R.H. GUTHRIE, *RECONSTRUCTION OF THE FEMALE BREAST FOLLOWING RADICAL MASTECTOMY.* Plastic and Reconstructive Surgery, 1971. **47**(6): p. 565-567.

For peer review only - http://bmjopen.bmj.com/site/about/guidelines.xhtml

17. Gruber, R.P., et al., *Breast Reconstruction Following Mastectomy: A Comparison of Submuscular and Subcutaneous Techniques.* Plastic & Reconstructive Surgery, 1981. **67**(3): p. 312-317.
18. Potter, S., et al., *Short-Term Safety Outcomes of Mastectomy and Immedaite Implant-Based Breast reconstruction With and Without Mesh (iBRA): a Multicentre, Propospective Cohort Study.* Lancet Oncology, 2019. **20**(2): p. 254-266.
19. Ergina, P.L., et al., *Challenges in evaluating surgical innovation.* Lancet, 2009. **374**: p. 1097-1104.
20. Pennell, C.P., et al., *Practical guide to the Idea, Development and Exploration stages of the IDEAL Framework and Recommendations.* British Journal of Surgery, 2016. **103**(5): p. 607-615.
21. Hirst, A., et al., *No Surgical Innovation without Evaluation: Evolution and Further Development of the IDEAL Framework and Recommendations.* Annals of Surgery, 2019. **269**(2): p. 211-220.
22. Martin, L., et al., *Acellular dermal matrix (ADM) assisted breast reconstruction procedures: joint guidelines from the Association of Breast Surgery and the British Association of Plastic, Reconstructive and Aesthetic Surgeons.* European Journal of Surgical Oncology, 2013. **39**(5): p. 425-9.
23. Blencowe, N.S., et al., *Standardizing and monitoring the delivery of surgical interventions in randomized clinical trials.* British Journal of Surgery, 2016. **103**(10): p. 1377-1384.
24. Barr, S.P., et al., *Infection prevention in breast implant surgery - A review of the surgical evidence, guidelines and a checklist.* European Journal of Surgical Oncology, 2016. **42**: p. 591-603.

14. APPENDICECS

i. Site Survey
ii. Surgeon Survey
iii. Surgeon Information Sheet
iv. Patient Information Sheet (PIS)
v. Case Report Form (CRF)
vi. Informed Consent Form (ICF)
vii. Breast-Q Questionnaire
viii. Screening Log
ix. NWBRC 'TIC' List
x. Topic Guides for Surgeon Interviews (1,2, and 3)
xi. Local Site Proposed Patient Identifiable Data Sheet
xii. Schedule of Insurance
xiii. HRA Statement of activity
xiv. HRA Schedule of activities

For peer review only - http://bmjopen.bmj.com/site/about/guidelines.xhtml

The Pre-BRA (Pre-pectoral Breast Reconstruction Evaluation) Feasibility Study: Protocol
KL Harvey, N Mills, P White, C Holcombe, S Potter
Appendix 1.

Appendix 1.

CASE REPORT FORM

**Unit Name**	
**REDCap Record ID***											

Record ID = * Unit code+### e.g. NBT001, NBT002 etc.

**Participant email address**	

For peer review only

The Pre-BRA (Pre-pectoral Breast Reconstruction Evaluation) Feasibility Study: Protocol
KL Harvey, N Mills, P White, C Holcombe, S Potter
Appendix 1.

Pre-Op and Operative Data

Pre-BRA

For peer review only

The Pre-BRA (Pre-pectoral Breast Reconstruction Evaluation) Feasibility Study: Protocol
KL Harvey, N Mills, P White, C Holcombe, S Potter
Appendix 1.

Participant Data

Participant withdrawn? Yes ☐ No ☐

(*Only tick 'yes' if the patient no longer wishes to participate in the study)*

Participant questionnaire completion: *(includes BREAST-Q PROMS and Pain Scores)*

Baseline* on paper, follow-ups** online ☐

Everything online ☐

Everything on paper - *PLEASE INFORM CENTRAL STUDY TEAM* ☐

*Baseline defined as: baseline (pre-operative) BREAST-Q PROMS and 24-hours post-op pain score.
**Follow-ups defined as: 3-month and 18-month BREAST-Q PROMS and 1 week, 2 weeks and 3-months pain scores

IF the patient has chosen to complete baseline PROMS on paper and follow-ups online, please offer the participant:

i. a PAPER copy of the Pre-Operative BREAST-Q AND
ii. ask them to record 24-hour post-op pain score in person (/over the telephone if discharged)
(These results need to be transcribed into REDCap by a member of the research team)
iii. All other PROMS will be requested via email direct to the patient

IF the patient has chosen to complete all online PLEASE NOTE:

The patient's record including email address and date of surgery **must** be saved on REDCap on or before the day of surgery in order to generate electronic baseline (pre-operative) BREAST-Q and 24hours post-op pain score requests.

IF the patient has chosen to complete everything on paper, please offer the participant:

i. a PAPER copy of the Pre-Operative BREAST-Q for completion prior to surgery
ii. PAPER copies of the Post-operative BREAST-Q for completion at 3 and 18 months post-operatively (could be sent by post) AND
iii. Ask them to record 24 hour post-op pain score in person (/over the telephone if discharged)
iv. 1 week, 2 weeks and 3 months post-op pain scores can be collected by telephone call from a member of the research team.
(These results need to be transcribed into REDCap by a member of the research team)

For peer review only

For peer review only - http://bmjopen.bmj.com/site/about/guidelines.xhtml

Pre-BRA Section 1 - Pre-operative data

Co-morbidity data

Patient age (in years) on entering the study .... **Years**

Planned* **date of primary mastectomy and reconstruction**
*(*Required to generate automatic electronic BREAST-Q PROMS and post-operative pain score requests via REDCap)*

........./........../..........(DD/MM/YYYY)

Laterality of planned surgery?

Right breast ☐ Left Breast ☐ Bilateral ☐

Indication for RIGHT mastectomy and planned reconstruction? (leave blank if n/a)

Risk reducing ☐ Malignancy ☐

Indication for LEFT mastectomy and planned reconstruction? (leave blank if n/a)

Risk reducing ☐ Malignancy ☐

Answer ONLY IF indication for surgery is malignancy: (leave blank if n/a)

RIGHT BREAST:

First operation for cancer ☐
Completion mastectomy* ☐
Local recurrence ☐

LEFT BREAST:

First operation for cancer ☐
Completion mastectomy* ☐
Local recurrence ☐

** i.e. Mastectomy performed following unsuccessful breast conservation surgery for example wide local excision (WLE) or therapeutic mammoplasty (TM)*

Medical co-morbidities?

Yes ☐ No ☐

Answer ONLY IF medical comorbidities present: (please tick all relevant)

Diabetes ☐
Asthma/ COPD ☐
Ischaemic heart disease ☐
Connective tissue disease ☐
Current steroid therapy ☐
Anticoagulant therapy (prior to surgery) ☐
Other co-morbidity ☐

If other, please supply details: ……………………………………………………………………..

For peer review only - http://bmjopen.bmj.com/site/about/guidelines.xhtml

The Pre-BRA (Pre-pectoral Breast Reconstruction Evaluation) Feasibility Study: Protocol
KL Harvey, N Mills, P White, C Holcombe, S Potter
Appendix 1.

Prior and neo-adjuvant treatments

Previous radiotherapy to ipsilateral breast or chest wall
(MEETS RELATIVE EXCLUSION CRITERIA)

Yes ☐ No ☐

Neo-adjuvant chemotherapy prior to mastectomy and reconstruction?

Yes ☐ No ☐

Previous surgery to ipsilateral breast?

No ☐ (Please go to Section 2))

Yes ☐

(REDCap™ will open the following options, please tick all relevant and indicate date of surgery:)

Previous axillary surgery ☐ Year................................

Wide local excision ☐ Year...............................

Augmentation ☐ Year................................

Reduction ☐ Year................................

Other ☐ Year................................

Pre-BRA Section 2 - Assessment of suitability for pre-pectoral breast reconstruction

Smoking status **(RELATIVE EXCLUSION if current smoker)**

Non-smoker ☐

Current smoker ☐

Nicotine replacement/ vaping with nicotine ☐

Recent ex-smoker (i.e. stopped at point of diagnosis) ☐

Grade of ptosis

0 No ptosis ☐

1 Mild - Nipple at level of IMF and above most of lower breast tissue ☐

2 Moderate - Nipple below IMF, but higher than most of the breast tissue ☐

3 Advanced - Nipple below IMF and at level of maximal breast projection ☐

4 Severe - Nipple far below IMF and points towards the floor ☐

Predicted implant vol (cc) ....

For peer review only - http://bmjopen.bmj.com/site/about/guidelines.xhtml

The Pre-BRA (Pre-pectoral Breast Reconstruction Evaluation) Feasibility Study: Protocol
KL Harvey, N Mills, P White, C Holcombe, S Potter
Appendix 1.

(>600cc fixed volume **MEETS RELATIVE EXCLUSION CRITERIA***)*

Anticipated adjuvant therapy (RELATIVE EXCLUSION if PMRT is anticipated)

Post-mastectomy radiotherapy?

Not required (risk reduction) ☐
No, previously received ☐
Unlikely (e.g. DCIS only) ☐
Possible ☐
Probable
(positive nodes, large tumour) ☐

Body Mass Index

Patient height (cm) .... Patient weight (kg) ....

BMI (REDCap™ calculates) .... **(RELATIVE EXCLUSION if >30)**

Manual BMI entry *(only answer if height/ weight values unknown)* ....

Suitability for pre-pectoral reconstruction

RELATIVE CONTRA-INDICATIONS:

- Previous breast/ chest wall radiotherapy or mantle radiotherapy
- Anticipated post-mastectomy radiotherapy
- BMI >30
- Current smoker (or nicotine dependent)
- Implant volume >600cc

What is your clinical impression of this patient's risk of experiencing a post-operative complication?

They are at low risk ☐ (Defined as no relative contraindications)

They are at moderate risk ☐ (Defined as 1 relative contra-indication)

They are at high risk ☐ (Defined as >1 relative contraindication)

They are high risk for another reason ☐

Please supply details if you have specified another reason

....

....

....

Are any additional surgical procedures planned at a later date? *(please select all that apply)*

For peer review only - http://bmjopen.bmj.com/site/about/guidelines.xhtml

The Pre-BRA (Pre-pectoral Breast Reconstruction Evaluation) Feasibility Study: Protocol
KL Harvey, N Mills, P White, C Holcombe, S Potter
Appendix 1.

Lipo-modelling ☐

Nipple reconstruction ☐

Contralateral symmetrising Reduction/ Mastopexy/ Augmentation ☐

No further procedures planned ☐

Pre-BRA Section 3 - Modifications to Usual Patient Selection Criteria

Have you modified your usual patient selection criteria in order to offer this patient a pre-pectoral implant reconstruction?

Yes ☐ No ☐

If you have answered "yes", please explain in what way: ....

....

....

....

....

Note: The study team are very interested in speaking to surgeons to explore any modifications made to their usual patient selection criteria.

Does the operating surgeon consent to be contacted for a brief telephone interview?

Yes ☐ No ☐

Please supply the email address of the operating surgeon if yes:

....

2.12 FINAL STUDY ENTRY CHECKLIST

**2.8.1 Meets inclusion/exclusion criteria for the study**	**Yes/No**
**2.8.2 Written consent to participate in the study**	**Yes/No**
**2.8.3 Pre-op PROMs (Breast-Q) completed**	**Yes/No**

For peer review only - http://bmjopen.bmj.com/site/about/guidelines.xhtml

The Pre-BRA (Pre-pectoral Breast Reconstruction Evaluation) Feasibility Study: Protocol
KL Harvey, N Mills, P White, C Holcombe, S Potter
Appendix 1.

Pre-BRA Section 4 - Operative data

Actual* **date of primary mastectomy and reconstruction**

………/………../……….(DD/MM/YYYY)

**If the planned (see section 1.) and actual dates of surgery differ, please contact central study team to confirm which date is correct. This is important for generating electronic BREAST-Q PROMS and post-operative pain score requests.*

ASA grade

1	Normal healthy individual	☐
2	Mild systemic disease that does not limit activities	☐
3	Severe systemic disease that limits activities but is not incapacitating	☐
4	Incapacitating systematic disease which is constantly life-threatening	☐

Name of operating Consultant surgeon

………

Procedure performed in theatre with laminar flow system in situ?

Yes ☐ No ☐

Type of skin prep used at time of surgery

Iodine (Betadine) (brown)	☐	Chlorhexidine (pink)	☐
2% Chlorprep	☐	Other (please specify)	☐

………

Planned antibiotic use

Prophylactic only (<24 hours)	☐	1-5 days	☐
Extended course (5+ days)	☐	Until drains removed	☐
Other (please specify)	☐		

………

Antibiotics used, please include dose/ frequency (free text)

………

Duration of procedure (knife to skin to dressings on) ……………………………(Minutes)

For peer review only - http://bmjopen.bmj.com/site/about/guidelines.xhtml

The Pre-BRA (Pre-pectoral Breast Reconstruction Evaluation) Feasibility Study: Protocol
KL Harvey, N Mills, P White, C Holcombe, S Potter
Appendix 1.

Did you adhere to the steps advised in The Theatre Implant Checklist or 'TIC List'? (REDCap™ displays "TIC" List) Please tick all steps which were completed in this case:

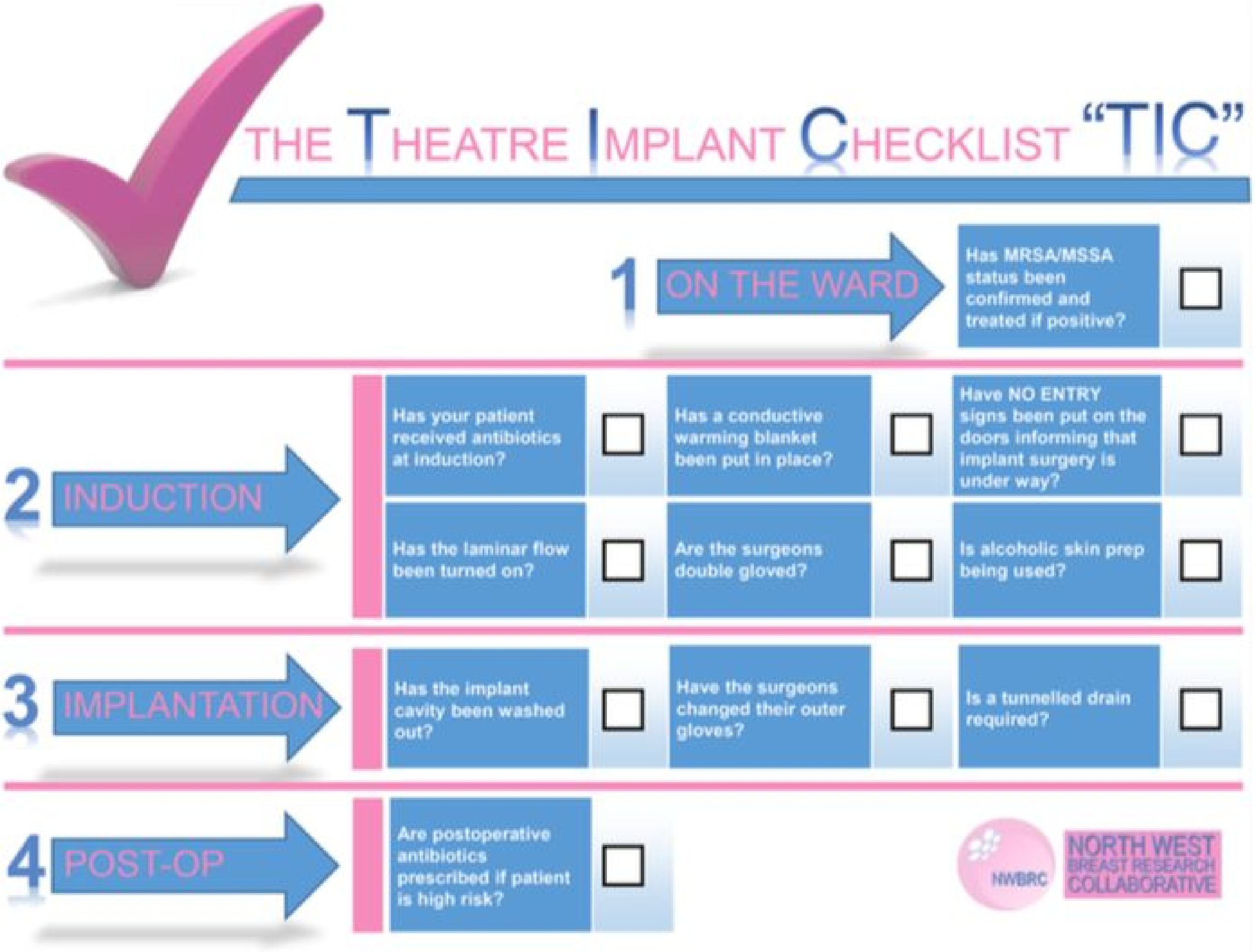

(Image reproduced from Barr, S.P., et al., *Infection prevention in breast implant surgery - A review of the surgical evidence, guidelines and a checklist.* European Journal of Surgical Oncology, 2016. **42**: p. 591-603.)

	<u>**Yes /**</u>	<u>**No**</u>
Patient received antibiotics at induction?	☐	☐
Conductive warming blanket been put in place?	☐	☐
NO ENTRY signs been put on the doors informing that implant surgery is under way?	☐	☐
Are the surgeons double gloved?	☐	☐
Is alcoholic skin prep being used?	☐	☐
Implant cavity washed out?	☐	☐
(Before implantation) Have the surgeons changed their outer gloves?	☐	☐
Is a tunnelled drain required?	☐	☐
Are post-operative antibiotics prescribed if patient is high risk?	☐	☐

For peer review only - http://bmjopen.bmj.com/site/about/guidelines.xhtml

The Pre-BRA (Pre-pectoral Breast Reconstruction Evaluation) Feasibility Study: Protocol
KL Harvey, N Mills, P White, C Holcombe, S Potter
Appendix 1.

Regarding length of stay, is the patient planned for:

Day case (i.e. home the same day as surgery) ☐

Overnight stay (i.e. home the day after surgery) ☐

Inpatient stay (>1 night) ☐

Was surgery performed on: (please tick all that apply)

Right breast ☐ Left breast ☐

**Right breast procedure:**		**Left breast procedure:**	
Implant reconstruction	☐	Implant reconstruction	☐
Other reconstruction	☐	Other reconstruction	☐
Augmentation	☐	Augmentation	☐
Reduction	☐	Reduction	☐
Mastopexy	☐	Mastopexy	☐
Other	☐	Other	☐

If other selected, please give details: ……………………………………………………………..

………

For peer review only - http://bmjopen.bmj.com/site/about/guidelines.xhtml

The Pre-BRA (Pre-pectoral Breast Reconstruction Evaluation) Feasibility Study: Protocol
KL Harvey, N Mills, P White, C Holcombe, S Potter
Appendix 1.

Pre-BRA Section 5a. RIGHT BREAST DATA

(Please only answer Section 5a. if you have performed a PRE-PECTORAL IMPLANT RECONSTRUCTION on the patient's RIGHT BREAST)

Pre-operative Planning: RIGHT BREAST

Right breast – What do you plan to do upfront i.e. prior to starting the operation?

One stage reconstruction	☐	Two-stage reconstruction	☐

Intra-operative Detail

Right breast - Grade of primary operating surgeon

Consultant	☐	SAS grade	☐
Senior trainee (OPF/ST8+)	☐	ST6/7	☐
ST5 or below	☐	Other	☐

If other selected, please give details: ………………………………………………………………..

Right breast - Number of pre-pectoral implant-based reconstructions using this method/ technique the primary surgeon has **previously performed:**

In total (i.e. both supervised and unsupervised):

Less than 5	☐	11-25	☐
5-10	☐	More than 25	☐

Supervised:

Less than 5	☐	11-25	☐
5-10	☐	More than 25	☐

Right breast - Type of mastectomy

Skin sparing mastectomy	☐	Skin and nipple preserving	☐
Reduction (Wise) pattern	☐	Other	☐

If other selected, please give details: ………………………………………………………………..

……

For peer review only - http://bmjopen.bmj.com/site/about/guidelines.xhtml

The Pre-BRA (Pre-pectoral Breast Reconstruction Evaluation) Feasibility Study: Protocol
KL Harvey, N Mills, P White, C Holcombe, S Potter
Appendix 1.

Right breast - Location of incision

Peri-areolar (nipple sparing) ☐ Lateral ☐
Inframammary ☐ Elliptical removing NAC ☐
Wise pattern ☐ Other ☐

If other selected, please give details: ……………………………………………………………..

………

Right breast – Was infiltration in the mastectomy plane i.e. hydro-dissection used?

No ☐ With saline only ☐
With local anaesthetic +/- saline ☐ LA and adrenaline +/- saline ☐

Right breast - Main instrument used for dissection of mastectomy skin flaps

Blade ☐ Scissors ☐
Handheld monopolar diathermy ☐ Diathermy scissors ☐
Combination of methods ☐ Other ☐

If other selected, please give details: ……………………………………………………………..

………

Right breast - Mastectomy dry weight (gms) ………………………………………

<u>Feasibility of Pre-Pectoral Reconstruction</u>

Right breast - Surgeon's assessment of skin flap quality

Good – healthy, well-perfused, no concerns noted at time of surgery ☐
Average – intermediate – no obvious concerns at time of surgery ☐
Poor – thin flaps, questionable vascularity; concerns at time of surgery ☐

Right breast - Did you complete the reconstruction as planned? (i.e. as per your answer to the first question of Section 5?)

Yes ☐ No ☐

For peer review only - http://bmjopen.bmj.com/site/about/guidelines.xhtml

The Pre-BRA (Pre-pectoral Breast Reconstruction Evaluation) Feasibility Study: Protocol
KL Harvey, N Mills, P White, C Holcombe, S Potter
Appendix 1.

Right breast - What operation did you actually perform?

i. Pre-pectoral approach considered safe and performed with fixed volume implant ☐

ii. Pre-pectoral reconstruction performed with variable volume implant (e.g. Becker) ☐

iii. Pre-pectoral reconstruction performed with a tissue expander ☐

iv. Pre-pectoral reconstruction abandoned ☐

If you selected answer iv. please also answer the next two questions ("Why?" and "What did you do instead?")

If you answered i, ii or iii please go straight to the third question down ("Right breast - type of implant coverage")

Why was pre-pectoral approach abandoned?

…………………………………………………………………………………………

…………………………………………………………………………………………..

…………………………………………………………………………………………

What procedure was performed instead

No reconstruction ☐

Sub-pectoral reconstruction with tissue expander (no mesh) ☐

Sub-pectoral reconstruction with biological mesh ☐

Sub-pectoral reconstruction with synthetic mesh ☐

Other ☐

If other selected, please give details: **…………………………………………………………………..**

……

Right breast - type of implant coverage

No mesh or ADM coverage (subcutaneous implant only) ☐

Biological mesh (e.g. ADM) ☐

Synthetic mesh (e.g. TiLOOP) ☐

Complete dermal sling ☐

Dermal sling and biological mesh in combination ☐

For peer review only - http://bmjopen.bmj.com/site/about/guidelines.xhtml

Dermal sling and synthetic mesh in combination ☐

Hybrid implant coverage (e.g. biological mesh at top/synthetic mesh at bottom) ☐

Other ☐

If 'Hybrid implant coverage' or 'other' selected, please supply details: ……………………………

………

Right breast - type of implant coverage, if you selected 'Biological mesh' please select which type:

BRAXON ☐ Artia ☐

Surgimend ☐ Fortiva ☐

Strattice ☐ Cellis ☐

Veritas ☐ Exaflex Pocket ☐

MESO ☐ Native ☐

Tutomesh ☐

Other biological ADM/ mesh (please state) ……………………………………………………☐

………

Right breast - type of implant coverage, if you selected 'synthetic mesh' please select which type:

TiLOOP ☐ Galaflex ☐

TiGR ☐

Other (please state) ……………………………………………………………………………☐

Right breast - Breast prosthesis details

Fixed volume implant ☐

Size (ccs)……………………………………………

OR

Temporary tissue expander ☐

Vol of saline inserted (mls)………………………….

Size when fully expanded (ccs)……………………..

OR

Combined implant (Beckers) ☐

For peer review only - http://bmjopen.bmj.com/site/about/guidelines.xhtml

The Pre-BRA (Pre-pectoral Breast Reconstruction Evaluation) Feasibility Study: Protocol
KL Harvey, N Mills, P White, C Holcombe, S Potter
Appendix 1.

Silicone component (g).......................... Vol of saline inserted (mls)…......................

Size when fully expanded (ccs)........................

Right breast – shape of implant

Round ☐ Anatomical ☐

Right breast – texture of implant

Smooth ☐ Textured/ Microtextured ☐

Polyurethane ☐

Right breast – Was the prosthesis bathed/washed prior to insertion?

Yes ☐ No ☐

If 'Yes' selected which wash was used?

Saline ☐ Antiseptic ☐

Antibiotics ☐ Other (please give details) ☐

....

Right breast – Was the cavity washed or irrigated prior to insert of implant?

Yes ☐ No ☐

If 'Yes' selected what irrigation fluid was used?

Saline ☐ Antiseptic ☐

Antibiotics ☐ Other (please give details) ☐

....

Surgeon glove change prior to handling the implant?

Yes ☐ No ☐

Right Breast - Were the skin edges freshened/excised prior to wound closure?

Yes ☐ No ☐

Right breast – Type of axillary surgery

None ☐

For peer review only - http://bmjopen.bmj.com/site/about/guidelines.xhtml

For peer review only

The Pre-BRA (Pre-pectoral Breast Reconstruction Evaluation) Feasibility Study: Protocol
KL Harvey, N Mills, P White, C Holcombe, S Potter
Appendix 1.

SLNB/targeted sample post NAC ☐

Axillary sample (failed SLNB) ☐

Axillary clearance ☐

SLNB + ANC (e.g. positive OSNA) ☐

Right breast – Number of (tunnelled) drains used (per side if bilateral case)

None ☐ One ☐ Two ☐

Right breast – intra-operative analgesia used

Local/regional block (e.g. pectoral or serratus block) ☐

Infiltration of LA as part of hydro dissection/tumescence ☐

Infiltration of LA into wounds prior to starting case ☐

Infiltration of LA into wounds at the end of the case ☐

LA inserted down drains ☐

Intra-pocket LA catheter (continuous infusion of LA post-op) ☐

LA not used ☐

Other ☐

Right breast dressings used (please select as many as apply)

Occlusive adhesive dressing (e.g. Opsite) ☐

Skin glue (e.g. Liquiband) ☐

Skin closure system - skin glue and tape (e.g. Dermabond Prineo dressing) ☐

Negative pressure dressing e.g. PICO ☐

Other ☐

For peer review only - http://bmjopen.bmj.com/site/about/guidelines.xhtml

The Pre-BRA (Pre-pectoral Breast Reconstruction Evaluation) Feasibility Study: Protocol
KL Harvey, N Mills, P White, C Holcombe, S Potter
Appendix 1.

Pre-BRA Section 5b. LEFT BREAST DATA

(Please only answer Section 5b. if you have performed a PRE-PECTORAL IMPLANT RECONSTRUCTION on the patient's LEFT BREAST)

Pre-operative Planning: LEFT BREAST

Left breast – What do you plan to do upfront i.e. prior to starting the operation?

One stage reconstruction	☐	Two-stage reconstruction	☐

Intra-operative Detail

Left breast - Grade of primary operating surgeon

Consultant	☐	SAS grade	☐
Senior trainee (OPF/ST8+)	☐	ST6/7	☐
ST5 or below	☐	Other	☐

If other selected, please give details: ……………………………………………………………..

Left breast - Number of pre-pectoral implant-based reconstructions using this method the primary surgeon has **previously performed:**

In total (i.e. both supervised and unsupervised):

Less than 5	☐	11-25	☐
5-10	☐	>25	☐

Supervised:

Less than 5	☐	11-25	☐
5-10	☐	>25	☐

Left breast - Type of mastectomy

Skin sparing mastectomy	☐	Skin and nipple preserving	☐
Reduction (wise) pattern	☐	Other	☐

If other selected, please give details: ……………………………………………………………..

………

For peer review only - http://bmjopen.bmj.com/site/about/guidelines.xhtml

The Pre-BRA (Pre-pectoral Breast Reconstruction Evaluation) Feasibility Study: Protocol
KL Harvey, N Mills, P White, C Holcombe, S Potter
Appendix 1.

Left breast - Location of incision

Peri-areolar (nipple sparing)	☐	Lateral	☐
Inframammary	☐	Elliptical removing NAC	☐
Wise pattern	☐	Other	☐

If other selected, please give details: ……………………………………………………………..

………

Left breast – Was infiltration in the mastectomy plane/hydro-dissection used?

No	☐	With saline only	☐
With local anaesthetic +/- saline	☐	LA and adrenaline +/- saline	☐

Left breast - Main instrument used for dissection of mastectomy skin flaps

Blade	☐	Scissors	☐
Handheld monopolar diathermy	☐	Diathermy scissors	☐
Combination of methods	☐	Other	☐

If other selected, please give details: ……………………………………………………………..

………

Left breast - Mastectomy dry weight (gms) ……………………………………….

<u>Feasibility of Pre-Pectoral Reconstruction</u>

Left breast - Surgeon's assessment of skin flap quality

Good – healthy, well-perfused, no concerns noted at time of surgery	☐
Average – intermediate – no obvious concerns at time of surgery	☐
Poor – thin flaps, questionable vascularity; concerns at time of surgery	☐

Left breast - Did you complete the reconstruction as planned? (i.e. as per your answer to the first question of Section 3b?)

Yes ☐ No ☐

Left breast – if 'no', what operation did you actually perform?

For peer review only - http://bmjopen.bmj.com/site/about/guidelines.xhtml

The Pre-BRA (Pre-pectoral Breast Reconstruction Evaluation) Feasibility Study: Protocol
KL Harvey, N Mills, P White, C Holcombe, S Potter
Appendix 1.

i. Pre-pectoral approach considered safe and performed with fixed volume implant ☐

ii. Pre-pectoral reconstruction performed with variable volume implant (e.g. Becker) ☐

iii. Pre-pectoral reconstruction performed with a tissue expander ☐

iv. Pre-pectoral reconstruction abandoned ☐

If you selected answer iv. please also answer the next two questions ("Why?" and "What did you do instead")

If you answered i, ii or iii please go straight to the third question down ("Left breast - type of implant coverage")

Why was pre-pectoral approach abandoned?

……………………………………………………………………………………

……………………………………………………………………………………..

………………………………………………………………………………………

What procedure was performed instead

No reconstruction ☐

Sub-pectoral reconstruction with tissue expander (no mesh) ☐

Sub-pectoral reconstruction with biological mesh ☐

Sub-pectoral reconstruction with synthetic mesh ☐

Other ☐

Left breast - type of implant coverage

No mesh or ADM coverage (subcutaneous implant only) ☐

Biological mesh (e.g. ADM) ☐

Synthetic mesh (e.g. TiLOOP) ☐

Complete dermal sling ☐

Dermal sling and biological mesh in combination ☐

Dermal sling and synthetic mesh in combination ☐

For peer review only - http://bmjopen.bmj.com/site/about/guidelines.xhtml

The Pre-BRA (Pre-pectoral Breast Reconstruction Evaluation) Feasibility Study: Protocol
KL Harvey, N Mills, P White, C Holcombe, S Potter
Appendix 1.

Hybrid implant coverage (e.g. biological mesh at top/synthetic mesh at bottom) ☐

Other ☐

If 'Hybrid implant coverage' or 'other' selected, please supply details: ……………………………

....

Left breast - type of implant coverage, if you selected 'Biological mesh' please select which type:

BRAXON	☐	Artia	☐
Surgimend	☐	Fortiva	☐
Strattice	☐	Cellis	☐
Veritas	☐	Exaflex Pocket	☐
MESO	☐	Native	☐
Tutomesh	☐		

Other biological ADM/ mesh (please state) ....☐

....

Left breast - type of implant coverage, if you selected 'synthetic mesh' please select which type:

TiLOOP	☐	Galaflex	☐
TiGR	☐		

Other (please state) ....☐

Left breast - Breast prosthesis details

Fixed volume implant ☐

Size (ccs)...

OR

Temporary tissue expander ☐

Vol of saline inserted (mls)…...........................

Size when fully expanded (ccs).........................

OR

Combined implant (Beckers) ☐

Silicone component (g)......................... Vol of saline inserted (mls)….......................

For peer review only - http://bmjopen.bmj.com/site/about/guidelines.xhtml

For peer review only

The Pre-BRA (Pre-pectoral Breast Reconstruction Evaluation) Feasibility Study: Protocol
KL Harvey, N Mills, P White, C Holcombe, S Potter
Appendix 1.

Size when fully expanded (ccs)........................

Left breast – shape of implant

Round ☐ Anatomical ☐

Left breast – texture of implant

Smooth ☐ Textured/ Microtextured ☐

Polyurethane ☐ I don't know ☐

Left breast – Was the prosthesis bathed/washed prior to insertion?

Yes ☐ No ☐

If 'Yes' selected which wash was used?

Saline ☐ Antiseptic ☐

Antibiotics ☐ Other (please give details) ☐

....

Left breast – Was the cavity washed or irrigated prior to insert of implant?

Yes ☐ No ☐

If 'Yes' selected what irrigation fluid was used?

Saline ☐ Antiseptic ☐

Antibiotics ☐ Other (please give details) ☐

....

Left Breast - Surgeon glove change prior to handling the implant?

Yes ☐ No ☐

Left Breast - Were the skin edges freshened/excised prior to wound closure?

Yes ☐ No ☐

Left breast – Type of axillary surgery

None ☐ (Please go to question 3.36)

SLNB/targeted sample post NAC ☐ Axillary sample (failed SLNB) ☐

Axillary clearance ☐ SLNB + ANC (e.g. positive OSNA) ☐

For peer review only - http://bmjopen.bmj.com/site/about/guidelines.xhtml

The Pre-BRA (Pre-pectoral Breast Reconstruction Evaluation) Feasibility Study: Protocol
KL Harvey, N Mills, P White, C Holcombe, S Potter
Appendix 1.

Left breast – Number of (tunnelled) drains used

None ☐ One ☐ Two ☐

Left breast – intra-operative analgesia used

Local/regional block (e.g. pectoral or serratus block) ☐

Infiltration of LA as part of hydro dissection/tumescence ☐

Infiltration of LA into wounds prior to starting case ☐

Infiltration of LA into wounds at the end of the case ☐

LA inserted down drains ☐

LA not used ☐

Intra-pocket LA catheter (continuous infusion of LA post-op)

Other ☐

Left breast dressings used (select as many as apply)

Occlusive adhesive dressing (e.g. Opsite) ☐

Skin glue (e.g. Liquiband) ☐

Skin closure system - skin glue and tape (e.g. Dermabond Prineo dressing) ☐

Negative pressure dressing e.g. PICO ☐

Other

For peer review only - http://bmjopen.bmj.com/site/about/guidelines.xhtml

The Pre-BRA (Pre-pectoral Breast Reconstruction Evaluation) Feasibility Study: Protocol
KL Harvey, N Mills, P White, C Holcombe, S Potter
Appendix 1.

Pre-BRA Section 6 – Modifications to Usual Operative Technique

Were any modifications made to the operative technique used in this case?

Yes ☐ No ☐

If you have answered “**yes**”, please provide details about:

i. What was modified and how? (e.g. mesh sutured in a different way) ………………………...

……………………………………………………………………………………………………….

……………………………………………………………………………………………………….

……………………………………………………………………………………………………….

ii. Why was the modification made? (e.g. difficulty accessing mesh) …………………………...

……………………………………………………………………………………………………….

……………………………………………………………………………………………………….

……………………………………………………………………………………………………….

Please Note: The study team are very interested in speaking to surgeons to explore any modifications made to the procedure.

Does the operating surgeon consent to be contacted for a brief telephone interview?

Yes ☐ No ☐

Please supply the email address of the operating surgeon if yes:

………………………………………………………………………………………………

Pre-BRA Section 7 – Peri Discharge

Length of Stay

Was the patient managed as a day case?
(defined as: discharged on the same day as surgery)

Yes ☐ No ☐

For peer review only - http://bmjopen.bmj.com/site/about/guidelines.xhtml

The Pre-BRA (Pre-pectoral Breast Reconstruction Evaluation) Feasibility Study: Protocol
KL Harvey, N Mills, P White, C Holcombe, S Potter
Appendix 1.

Date of discharge (DD/MM/YYYY) ………………/……………/……………………..

Length of post-operative stay…………..………………… (no. of post-operative days in hospital, calculated from date of surgery (= Day 0) to date of discharge)

(REDCap will auto-populate using the answers from date of surgery and the answer above)

For peer review only

END OF PRE-OPERATIVE AND OPERATIVE DATA SECTION

For peer review only - http://bmjopen.bmj.com/site/about/guidelines.xhtml

The Pre-BRA (Pre-pectoral Breast Reconstruction Evaluation) Feasibility Study: Protocol
KL Harvey, N Mills, P White, C Holcombe, S Potter
Appendix 1.

Pain Scores

Pre-BRA

For peer review only

<u>Please NOTE:</u>

If the participant does not wish to complete pains core data entry online via email link they must be collected in person/ via telephone call and transcribed into REDCap's "Pain scores" section by the research team

**Pain score at 24 hours' post-reconstruction**	**(0 - 10)*** ………………
**Pain score at 1-week post-reconstruction:**	**(0 - 10)** ………………
**Pain score at 2 weeks' post-reconstruction:**	**(0 - 10)** ………………
**Pain score at 3 months' post-reconstruction:**	**(0 - 10)** ………………

* 0-10 Numeric pain rating scale, where:

0= No pain, 5= Moderate pain, 10= worst possible pain

For peer review only - http://bmjopen.bmj.com/site/about/guidelines.xhtml

The Pre-BRA (Pre-pectoral Breast Reconstruction Evaluation) Feasibility Study: Protocol
KL Harvey, N Mills, P White, C Holcombe, S Potter
Appendix 1.

3 Month Data

Pre-BRA

For peer review only

For peer review only - http://bmjopen.bmj.com/site/about/guidelines.xhtml

The Pre-BRA (Pre-pectoral Breast Reconstruction Evaluation) Feasibility Study: Protocol
KL Harvey, N Mills, P White, C Holcombe, S Potter
Appendix 1.

Pre-BRA Section 8 – Oncological outcome and adjuvant treatment data

Not applicable – risk reducing surgery ☐ (No further questions in Section 8)

RIGHT BREAST

(Only answer the following questions if you operated for malignancy on the patient's **right breast)**

Right breast – if neoadjuvant chemotherapy – complete pathological response?

Yes ☐ No ☐

Right breast - Invasive status

Invasive ☐ DCIS ☐

Right breast – Focality

Unifocal ☐ Multifocal ☐

Right breast - Grade of DCIS or invasive carcinoma

(from core biopsy if complete pathological response)

1 – Low grade (DCIS) or well-differentiated (invasive) ☐

2 – Intermediate grade (DCIS) or moderately differentiated (invasive) ☐

3 – High grade (DCIS) or poorly differentiated (invasive) ☐

Right breast - Lymph node involvement

Total number of involved nodes (macromets only) ....

Total number of lymph nodes in pathology specimen..

Right breast - Invasive lesion size (mm)

(largest lesion if multifocal) ....

Right side - Planned delayed axillary clearance

Yes ☐ No ☐

LEFT BREAST

(Only answer the following questions if you operated for malignancy on the patient's **left breast)**

Left breast – if neoadjuvant chemotherapy – complete pathological response?

Yes ☐ No ☐

Left breast - Invasive status

For peer review only - http://bmjopen.bmj.com/site/about/guidelines.xhtml

For peer review only

The Pre-BRA (Pre-pectoral Breast Reconstruction Evaluation) Feasibility Study: Protocol
KL Harvey, N Mills, P White, C Holcombe, S Potter
Appendix 1.

Invasive ☐ DCIS ☐

Left breast – Focality

Unifocal ☐ Multifocal ☐

Left breast - Grade of DCIS or invasive carcinoma
(from core biopsy if complete pathological response)

1 – Low grade (DCIS) or well-differentiated (invasive) ☐

2 – Intermediate grade (DCIS) or moderately differentiated (invasive) ☐

3 – High grade (DCIS) or poorly differentiated (invasive) ☐

Left breast - Lymph node involvement

6.13.1 Total number of involved nodes (macromets only) ....

6.13.2 Total number of lymph nodes in pathology specimen.......................................

Left breast - Invasive lesion size (mm)

(largest lesion if multifocal) ....

Left side - Planned delayed axillary clearance

Yes ☐ No ☐

PLANNED ADJUVANT THERAPY
(Answer the following questions for **all cases of malignancy**)

Chemotherapy

Yes ☐ No ☐ Already received ☐

Radiotherapy

Right side

to chest wall Yes ☐ No ☐

to axilla Yes ☐ No ☐

to SCF* Yes ☐ No ☐

to IMC** Yes ☐ No ☐

Left side

to chest wall Yes ☐ No ☐

to axilla Yes ☐ No ☐

to SCF* Yes ☐ No ☐

For peer review only - http://bmjopen.bmj.com/site/about/guidelines.xhtml

For peer review only

The Pre-BRA (Pre-pectoral Breast Reconstruction Evaluation) Feasibility Study: Protocol
KL Harvey, N Mills, P White, C Holcombe, S Potter
Appendix 1.

to IMC** Yes ☐ No ☐

(*Supraclavicular fossa, ** internal mammary chain)

Endocrine therapy Yes ☐ No ☐

Anti-Her2 therapy Yes ☐ No ☐

Date of FIRST adjuvant treatment
(i.e. date of first dose of chemotherapy, if applicable or 1st fraction of radiotherapy)

(DD/MM/YYYY)……………………………………………

Pre-BRA Section 9– Complication(s) at 3 Months

Post-operative complication experienced <u>IN THE FIRST 3 MONTHS</u>?

Yes ☐ No ☐

Readmission to hospital <u>IN THE FIRST 3 MONTHS</u>
Was the patient readmitted to hospital to manage a complication in the first 3 months post-operatively?

Yes ☐ No ☐ Date (DD/MM/YYYY) ……………………

If 'Yes' selected, please give reason for readmission:

………………………………………………………………………………………

……………………………………………………………………………………….

………………………………………………………………………………………

Re-operation for complications <u>IN THE FIRST 3 MONTHS</u>
Did the patient undergo an unplanned re-operation (i.e. following a complication) in the first 3 months post-operatively?

Yes ☐ No ☐ Date (DD/MM/YYYY) ……………………

If 'Yes' selected, please give reason for re-operation:

………………………………………………………………………………………

……………………………………………………………………………………….

………………………………………………………………………………………

For peer review only - http://bmjopen.bmj.com/site/about/guidelines.xhtml

For peer review only

The Pre-BRA (Pre-pectoral Breast Reconstruction Evaluation) Feasibility Study: Protocol
KL Harvey, N Mills, P White, C Holcombe, S Potter
Appendix 1.

Breast Complication Details:

Please indicate if a complication was experienced, indicate which side was affected and the date it was first noticed

	Right	Left	Bilateral	Date (DD/MM/YYYY)
**Seroma?**				
No aspiration required	☐	☐	☐	....
Requiring aspiration 1-2 times	☐	☐	☐	....
Requiring aspiration 3 or more times	☐	☐	☐	....
**Haematoma?**				
Minor - Managed conservatively	☐	☐	☐	....
Major 1 - Requiring aspiration in clinic (+/-USS, no GA)	☐	☐	☐	....
Major 2 - Requiring evacuation in theatre	☐	☐	☐	....
**Wound infection?**				
Minor - Requiring oral antibiotics	☐	☐	☐	....
Major 1 - Requiring admission for IV antibiotics	☐	☐	☐	....
Major 2 - Requiring surgical drainage or debridement	☐	☐	☐	....
**Mastectomy skin flap necrosis?**				
Minor – Managed conservatively	☐	☐	☐	....
Major 1 – Requiring surgical debridement in clinic (no GA)	☐	☐	☐	....
Major 2 – Requiring surgical debridement in theatre (GA)	☐	☐	☐	....
**Nipple necrosis?**				
Minor – Managed conservatively	☐	☐	☐	....
Major 1 – Requiring surgical debridement	☐	☐	☐	....
Major 2 – Total NAC loss	☐	☐	☐	....

For peer review only - http://bmjopen.bmj.com/site/about/guidelines.xhtml

The Pre-BRA (Pre-pectoral Breast Reconstruction Evaluation) Feasibility Study: Protocol
KL Harvey, N Mills, P White, C Holcombe, S Potter
Appendix 1.

	Right	Left	Bilateral	Date (DD/MM/YYYY)
**Wound dehiscence?**				
Minor – Managed conservatively	☐	☐	☐	....
Major – Requiring return to theatre for re-suturing	☐	☐	☐	....

Implant loss?

Total implant loss
i.e. any unplanned removal of the implant or expander for infection, **without replacement** of the prosthesis ☐ ☐ ☐ ....

OR ***Implant salvage*** i.e.

i. Implant removed, washed and replaced with fixed volume/ final implant
☐ ☐ ☐ ....

OR ii. Implant removed, washed and replaced with a tissue expander
☐ ☐ ☐ ....

(If "***Implant salvage***" selected, additional question:
Was implant salvage a success? (i.e. implant still in place at 3 months)

Yes ☐ No ☐

**Mechanical implant problem? (e.g. deflation)**	☐	☐	☐	....
**Other complication?**	☐	☐	☐	....

Details...

Pre-BRA Section 10 – Learning from Post-Operative Problems

Would the operating surgeon consider changing or modifying their **surgical technique** as a result of this complication? Yes ☐ No ☐

Please supply as much detail as possible: ....
....
....

For peer review only - http://bmjopen.bmj.com/site/about/guidelines.xhtml

The Pre-BRA (Pre-pectoral Breast Reconstruction Evaluation) Feasibility Study: Protocol
KL Harvey, N Mills, P White, C Holcombe, S Potter
Appendix 1.

………

Would the operating surgeon consider changing or modifying their **patient selection criteria** as a result of this complication? Yes ☐ No ☐

Please supply as much detail as possible: ………………………………………………………………

………

………

………

Please note: The study team are **very interested** in speaking to surgeons who report learning from post-operative problems.
Does the operating surgeon agree to be contacted for a brief telephone interview?

Yes ☐ No ☐

Please supply the email address of the operating surgeon if yes:

……………………………………………………………………………………………

Pre-BRA Section 11 – Post-Operative Patient Reported Outcome Measures (PROMS)

**7.1**	**3-month Breast-Q completed?**	Yes	☐	No	☐
**7.2**	**18-month Breast-Q completed?**	Yes	☐	No	☐

END OF QUESTIONS

THANK YOU FROM THE PRE-BRA TEAM!

Pre-BRA

For peer review only

The Pre-BRA (Pre-pectoral Breast Reconstruction Evaluation) Feasibility Study: Protocol
KL Harvey, N Mills, P White, C Holcombe, S Potter
Appendix 2.

Appendix 2.

Definitions of Complications

Implant loss - any unplanned removal of the implant without replacement of the prosthesis (implant or expander) for infection, wound problems or other indication within the first 3 months following surgery.

Implant salvage – return to theatre for debridement of wound/ re-suturing /drainage of infection/ washout/ or other indication in which the implant is removed and immediately replaced either with the same device or a tissue expander with primary closure of the wound.

Unsuccessful implant salvage (considered equivalent to implant loss) - the implant is no longer in situ 3 months after the salvage procedure was performed.

Infection - A hot, red swollen breast associated with one of the following; a temperature, pus at the wound site, a raised white cell count and/or; a positive wound swab within the first 3 months following surgery. This will be further classified as:

- Minor – requiring oral antibiotics only;
- Major 1 – requiring admission for IV antibiotics and/or debridement;
- Major 2 – requiring surgical drainage/debridement

Readmission to hospital – any re-admission to hospital in the 3 months following surgery directly related to the procedure (e.g. with infection requiring antibiotics).

For peer review only

The Pre-BRA (Pre-pectoral Breast Reconstruction Evaluation) Feasibility Study: Protocol
KL Harvey, N Mills, P White, C Holcombe, S Potter
Appendix 2.

Return to theatre – Return to the operating theatre at any time during the first 3 months following surgery to deal with any complication of the reconstruction. This will not include any secondary oncological procedures such as axillary clearance or planned procedures including exchange of expander for a fixed volume implant or lipo-modelling.

Seroma - A symptomatic collection of fluid around the reconstructed breast following surgery requiring aspiration. The total number of aspirations will be collected at 3 months.

Haematoma - A collection of blood in the reconstructed breast

- Minor – managed conservatively or by aspiration in clinic;
- Major – requiring surgical evacuation

Mastectomy skin flap necrosis - Any area of skin loss on the reconstructed breast

- Minor – managed conservatively with dressings;
- Major 1 – requiring debridement (in clinic or theatre) under local anaesthetic (LA);
- Major 2 – requiring surgical debridement under general anaesthesia (GA)

Nipple necrosis – Any area of necrosis of the nipple areolar complex (NAC) (if nipple preserving mastectomy)

- Minor – managed conservatively with dressings;
- Major 1 – requiring surgical debridement under LA in clinic or theatre;
- Major 2 – requiring surgical debridement under GA in theatre

Wound dehiscence – separation of the skin edges at the wound site.

- Minor – treated conservatively;
- Major – requiring return to theatre for re-suturing under GA

For peer review only

The Pre-BRA (Pre-pectoral Breast Reconstruction Evaluation) Feasibility Study: Protocol
KL Harvey, N Mills, P White, C Holcombe, S Potter
Appendix 2.

Displaced implant requiring repositioning under GA – any implant displacement that requires surgical correction to restore its position

In hospital complication – any complication that occurs during the patient's initial hospital stay at the time of their reconstructive surgery. This includes systematic complications such as DVT/PE and procedure specific complications such as haematoma.

Major complication **-** Any complication requiring readmission to hospital or return to theatre

Minor complication **-** Any other complication

Surgeons will be asked to report any learning arising from complications and whether they would be willing to be contacted to discuss this in more detail.

For peer review only

Appendix 3.

The Pre-Bra Study: Surgeon Survey

This survey is for surgeons performing pre-pectoral reconstruction who plan to recruit patients to the Pre-Bra study.

Part 1: Your Details

1. **Your name**……………………………………………………………………………………
2. **Name of your hospital**……………………………………………………………………………
3. **City where your hospital is based** ………………………………………………………………
4. **What year did you become a consultant surgeon/ associate specialist?**……………………
5. **Do you perform pre-pectoral breast reconstruction (PPBR)?** Yes ☐ No ☐

   (If you answered 'no" to question 5, no further answers are required. Thank you, you are not eligible to complete the questionnaire)

6. **If yes, how long have you been practising the technique of PPBR?**

<6 months	☐	2-5 years	☐
6 -12 months	☐	5-10 years	☐
1-2 years	☐	> 10 years	☐

7. **a. IF <6 months or 6-12 months selected:** approximately how many PPBR have you performed to date?

0-5	☐	11-20	☐	51-100	☐
6-10	☐	21-50	☐	>100	☐

   b. OR, IF >1 year selected: How many pre-pectoral reconstructions do you perform annually?

The Pre-BRA (Pre-pectoral Breast Reconstruction Evaluation) Feasibility Study: Protocol
KL Harvey, N Mills, P White, C Holcombe, S Potter
Appendix 3.

0-5	☐	11-20	☐	51-100	☐
6-10	☐	21-50	☐	>100	☐

8. Have you had training or mentorship in the technique of pre-pectoral reconstruction?

Yes ☐ No ☐

9. If you have answered yes to Q8. RedCAP™ offers the following options:

As a trainee? ☐ **OR** As a consultant? ☐

10. If you have answered yes to Q8. RedCAP™ offers the following options, please tick all that apply:

A National TIG Fellowship ☐

A training course ☐

An industry workshop/study day ☐

Visit to an established PPBR centre before performing your first case(s) ☐

Experienced PPBR surgeon-mentor in theatre during your first case(s) ☐

Another Oncoplastic fellowship (details below please) ☐

……………………………………………………………………………………………………

……………………………………………………………………………………………………

Other (please state) ☐ ………………………………………………………………………….............

……………………………………………………………………………………………………

……………………………………………………………………………………………………

For peer review only

The Pre-BRA (Pre-pectoral Breast Reconstruction Evaluation) Feasibility Study: Protocol
KL Harvey, N Mills, P White, C Holcombe, S Potter
Appendix 3.

Part 2: Your Patient Selection Criteria

11. Which factors affect your decision-making when offering a patient PPBR?

(please tick all that apply)

Breast size ☐ Patient age ☐

Patient BMI ☐ Degree of ptosis ☐

Patient hobbies or levels of activity ☐

Patient preference (e.g. to avoid implant animation) ☐

Likely need for post-mastectomy radiotherapy ☐

12. Which (if any) of the following would you consider a contraindication to pre-pectoral breast reconstruction? (please tick all that apply, RedCAP matrix)

	Not a contraindication	Relative contraindication	Absolute contraindication
Current smoker			
BMI >30			
Grade 3 or 4 ptosis			
Predicted implant volume >600 cc			
Breast size D cup or more			
Likely requirement for post-mastectomy radiotherapy			
Previous radiotherapy			
Thin skin flaps (e.g. pinch test <1cm)			

For peer review only

The Pre-BRA (Pre-pectoral Breast Reconstruction Evaluation) Feasibility Study: Protocol
KL Harvey, N Mills, P White, C Holcombe, S Potter
Appendix 3.

Diabetes			
Steroid use			
Connective tissue disease			

Part 3: Your Peri-operative Practice

13. What mesh(es) or ADMs do you use? (Please tick all that apply)

BRAXON ☐ TiLOOP ☐ MESO ☐

Strattice ☐ Fortiva ☐ TIGR ☐

Surgimend ☐ Artia ☐ Other ☐

(If other please give details –name and manufacturer)..

....

14. What is your mesh of choice/ preference? ....

....

15. Would you try a new mesh? Yes ☐ No ☐ Maybe ☐

16. If yes, what would make you consider trying something new?

Evaluation within a protocoled study ☐

Industry representative (rep) recommendation ☐

Evidence of Safety ☐

Any other reason ☐ ....

....

For peer review only

The Pre-BRA (Pre-pectoral Breast Reconstruction Evaluation) Feasibility Study: Protocol
KL Harvey, N Mills, P White, C Holcombe, S Potter
Appendix 3.

17. What implant(s) do you use? (Please tick all that apply)

Mentor ☐ Nagor ☐ B-Lite ☐

Allergan ☐ Eurosurgical ☐ Sebbin ☐

Other ☐ (please give details –name and manufacturer)......................................

....

18. Do you use tissue expanders in the PPBR population? Yes ☐ No ☐

19. If yes, how? (please select all that apply)

☐ I routinely perform two-stage PPBR with tissue expanders in all/ most cases

☐ I use two-stage surgery with tissue expanders in some patients I consider to be high risk (i.e. this is your planned pre-operative strategy)

☐ I convert to two-stage surgery with tissue expanders in some patients I consider to be high risk (i.e. this is an intra-operative decision based upon patient factors such as skin flaps)

☐ I use tissue expanders for PPBR in another way (please give details)

....

....

20. Do you have a protocol for the management of patients undergoing pre-pectoral reconstruction? (e.g. re: drains and antibiotics) Yes ☐ No ☐

21. How many post-operative drains to you routinely use?

None ☐ One ☐ Two ☐

other (please state) ☐...

For peer review only

The Pre-BRA (Pre-pectoral Breast Reconstruction Evaluation) Feasibility Study: Protocol
KL Harvey, N Mills, P White, C Holcombe, S Potter
Appendix 3.

……………………………………………………………………………………………………

22. If one/ two/ other: How long do the drains stay in? (free text)………………………………

……………………………………………………………………………………………………

23. What antibiotics do you use (name and dose) and for how long?.................................

……………………………………………………………………………………………………

24. Do you routinely use any of the following strategies to reduce infection? (Please tick all that apply):

Laminar flow theatre ☐ 'Clean' drapes ☐

Pocket wash ☐ 'Clean' instruments ☐

Tunnelling of drains ☐

Refreshing/trimming of skin edges prior to closure ☐

Glove change prior to implant insertion ☐

Part 4: Your Post-operative Practice

25. How many routine post-operative attendances are your implant-reconstruction patients invited to attend?

One ☐ Three ☐

Two ☐ >3 times ☐

Varies between cases ☐

Other…………………………………………………………………………………………

26. When do you see patients in the early post-operative period? (please select all that apply)

Before 1 week ☐ At 1 week ☐

For peer review only

The Pre-BRA (Pre-pectoral Breast Reconstruction Evaluation) Feasibility Study: Protocol
KL Harvey, N Mills, P White, C Holcombe, S Potter
Appendix 3.

At 2 weeks ☐ At 6 weeks ☐

At 3 weeks ☐ Beyond 6 weeks ☐

At 4 weeks ☐ It varies between cases ☐

At 5 weeks ☐ No routine follow-up ☐

Other ☐……………………………………………………………………………………

Part 5: Interviews with Surgeons

The study team are interested in identifying best practice for pre-pectoral breast reconstruction and would like to speak to surgeons performing the technique to understand how they do the operation, what works well and why.

27. Would you be prepared to be contacted by the study team to take part in a brief interview to discuss your approach to pre-pectoral breast reconstruction? All interviews will be conducted by telephone, should take no more than 20 minutes and information collected will be anonymised. Yes ☐ No ☐

28. If yes, please supply an Email address………………………………………………………………

END

Thank you for your answers

For peer review only

The Pre-BRA (Pre-pectoral Breast Reconstruction Evaluation) Feasibility Study: Protocol
KL Harvey, N Mills, P White, C Holcombe, S Potter
Appendix 4.

Local Trust Logo here

The Pre-BRA Feasibility Study
PATIENT CONSENT FORM

Please initial box

1 I confirm that I have read and understand the information sheet dated 28/03/2019 (version 2.0) for the above study. I have had the opportunity to consider the information, ask questions and have had these answered satisfactorily. ☐

2 I understand that my participation is voluntary and that I am free to withdraw at any time without giving any reason, without my medical care or legal rights being affected. ☐

3 I understand that relevant sections of my medical notes and data collected during the study may be looked at by individuals from regulatory authorities, the study research team or from the NHS Trust, where it is relevant to my taking part in this research. I give permission for these individuals to have access to my records. ☐

4 I agree that the study team may retain personally identifiable data collected during the study which could be used to directly support future research studies such as an extension to the Pre-Bra Study. For example, the study team may wish to contact me with further questionnaires, or to use identifiers such as my individual NHS number to interrogate national databases. ☐

5 I agree that data collected during the study may be retained by the study team for up to 10 years after the study has closed, in order to support future research studies. ☐

6 I agree to complete patient questionnaires for use in the study ☐

7 I agree to take part in the above study ☐

8 I would like to be sent a summary of the study's findings when the research is complete: Yes ☐ No ☐ ☐

I have supplied my email address for that purpose.

Email address: ____

Name of Patient	Date	Signature
Researcher Taking Consent	Date	Signature

One copy of the signed consent form should be given to the participant and one copy retained by the researcher.

IRAS Project ID: 255421 Patient Informed Consent Form v2.0 21/03/2019

For peer review only

Reporting checklist for protocol of a clinical trial.

Based on the SPIRIT guidelines.

Instructions to authors

Complete this checklist by entering the page numbers from your manuscript where readers will find each of the items listed below.

Your article may not currently address all the items on the checklist. Please modify your text to include the missing information. If you are certain that an item does not apply, please write "n/a" and provide a short explanation.

Upload your completed checklist as an extra file when you submit to a journal.

In your methods section, say that you used the SPIRITreporting guidelines, and cite them as:

Chan A-W, Tetzlaff JM, Altman DG, Laupacis A, Gøtzsche PC, Krleža-Jerić K, Hróbjartsson A, Mann H, Dickersin K, Berlin J, Doré C, Parulekar W, Summerskill W, Groves T, Schulz K, Sox H, Rockhold FW, Rennie D, Moher D. SPIRIT 2013 Statement: Defining standard protocol items for clinical trials. Ann Intern Med. 2013;158(3):200-207

		Reporting Item	Page Number
**Administrative information**			
Title	#1	Descriptive title identifying the study design, population, interventions, and, if applicable, trial acronym	1
Trial registration	#2a	Trial identifier and registry name. If not yet registered, name of intended registry	3
Trial registration: data set	#2b	All items from the World Health Organization Trial Registration Data Set	3
Protocol version	#3	Date and version identifier	*Not applicable*
Funding	#4	Sources and types of financial, material, and other support	19,20

For peer review only

Roles and responsibilities: contributorship	#5a	Names, affiliations, and roles of protocol contributors	19
Roles and responsibilities: sponsor contact information	#5b	Name and contact information for the trial sponsor	19
Roles and responsibilities: sponsor and funder	#5c	Role of study sponsor and funders, if any, in study design; collection, management, analysis, and interpretation of data; writing of the report; and the decision to submit the report for publication, including whether they will have ultimate authority over any of these activities	*Not applicable*
Roles and responsibilities: committees	#5d	Composition, roles, and responsibilities of the coordinating centre, steering committee, endpoint adjudication committee, data management team, and other individuals or groups overseeing the trial, if applicable (see Item 21a for data monitoring committee)	19
**Introduction**			
Background and rationale	#6a	Description of research question and justification for undertaking the trial, including summary of relevant studies (published and unpublished) examining benefits and harms for each intervention	5
Background and rationale: choice of comparators	#6b	Explanation for choice of comparators	5
Objectives	#7	Specific objectives or hypotheses	6
Trial design	#8	Description of trial design including type of trial (eg, parallel group, crossover, factorial, single group), allocation ratio, and framework (eg, superiority, equivalence, non-inferiority, exploratory)	7
**Methods: Participants, interventions, and outcomes**			
Study setting	#9	Description of study settings (eg, community clinic, academic hospital) and list of countries where data will be	7

For peer review only

		collected. Reference to where list of study sites can be obtained	
Eligibility criteria	#10	Inclusion and exclusion criteria for participants. If applicable, eligibility criteria for study centres and individuals who will perform the interventions (eg, surgeons, psychotherapists)	8
Interventions: description	#11a	Interventions for each group with sufficient detail to allow replication, including how and when they will be administered	10
Interventions: modifications	#11b	Criteria for discontinuing or modifying allocated interventions for a given trial participant (eg, drug dose change in response to harms, participant request, or improving / worsening disease)	10
Interventions: adherance	#11c	Strategies to improve adherence to intervention protocols, and any procedures for monitoring adherence (eg, drug tablet return; laboratory tests)	10
Interventions: concomitant care	#11d	Relevant concomitant care and interventions that are permitted or prohibited during the trial	11
Outcomes	#12	Primary, secondary, and other outcomes, including the specific measurement variable (eg, systolic blood pressure), analysis metric (eg, change from baseline, final value, time to event), method of aggregation (eg, median, proportion), and time point for each outcome. Explanation of the clinical relevance of chosen efficacy and harm outcomes is strongly recommended	12
Participant timeline	#13	Time schedule of enrolment, interventions (including any run-ins and washouts), assessments, and visits for participants. A schematic diagram is highly recommended (see Figure)	13
Sample size	#14	Estimated number of participants needed to achieve study objectives and how it was determined, including clinical and statistical assumptions supporting any sample size calculations	16
Recruitment	#15	Strategies for achieving adequate participant enrolment to reach target sample size	16

For peer review only

**Methods: Assignment of interventions (for controlled trials)**			
Allocation: sequence generation	#16a	Method of generating the allocation sequence (eg, computer-generated random numbers), and list of any factors for stratification. To reduce predictability of a random sequence, details of any planned restriction (eg, blocking) should be provided in a separate document that is unavailable to those who enrol participants or assign interventions	*Not applicable*
Allocation concealment mechanism	#16b	Mechanism of implementing the allocation sequence (eg, central telephone; sequentially numbered, opaque, sealed envelopes), describing any steps to conceal the sequence until interventions are assigned	*Not applicable*
Allocation: implementation	#16c	Who will generate the allocation sequence, who will enrol participants, and who will assign participants to interventions	*Not applicable*
Blinding (masking)	#17a	Who will be blinded after assignment to interventions (eg, trial participants, care providers, outcome assessors, data analysts), and how	*Not applicable*
Blinding (masking): emergency unblinding	#17b	If blinded, circumstances under which unblinding is permissible, and procedure for revealing a participant's allocated intervention during the trial	*Not applicable*
**Methods: Data collection, management, and analysis**			
Data collection plan	#18a	Plans for assessment and collection of outcome, baseline, and other trial data, including any related processes to promote data quality (eg, duplicate measurements, training of assessors) and a description of study instruments (eg, questionnaires, laboratory tests) along with their reliability and validity, if known. Reference to where data collection forms can be found, if not in the protocol	13
Data collection plan: retention	#18b	Plans to promote participant retention and complete follow-up, including list of any outcome data to be collected for	13

For peer review only

		participants who discontinue or deviate from intervention protocols	See appendix 4
Data management	#19	Plans for data entry, coding, security, and storage, including any related processes to promote data quality (eg, double data entry; range checks for data values). Reference to where details of data management procedures can be found, if not in the protocol	13, 19
Statistics: outcomes	#20a	Statistical methods for analysing primary and secondary outcomes. Reference to where other details of the statistical analysis plan can be found, if not in the protocol	17
Statistics: additional analyses	#20b	Methods for any additional analyses (eg, subgroup and adjusted analyses)	17
Statistics: analysis population and missing data	#20c	Definition of analysis population relating to protocol non-adherence (eg, as randomised analysis), and any statistical methods to handle missing data (eg, multiple imputation)	17
**Methods: Monitoring**			
Data monitoring: formal committee	#21a	Composition of data monitoring committee (DMC); summary of its role and reporting structure; statement of whether it is independent from the sponsor and competing interests; and reference to where further details about its charter can be found, if not in the protocol. Alternatively, an explanation of why a DMC is not needed	*Not applicable*
Data monitoring: interim analysis	#21b	Description of any interim analyses and stopping guidelines, including who will have access to these interim results and make the final decision to terminate the trial	17
Harms	#22	Plans for collecting, assessing, reporting, and managing solicited and spontaneously reported adverse events and other unintended effects of trial interventions or trial conduct	13
Auditing	#23	Frequency and procedures for auditing trial conduct, if any, and whether the process will be independent from investigators and the sponsor	*Not applicable*
**Ethics and dissemination**			

For peer review only

Research ethics approval	#24	Plans for seeking research ethics committee / institutional review board (REC / IRB) approval	3
Protocol amendments	#25	Plans for communicating important protocol modifications (eg, changes to eligibility criteria, outcomes, analyses) to relevant parties (eg, investigators, REC / IRBs, trial participants, trial registries, journals, regulators)	*Not applicable*
Consent or assent	#26a	Who will obtain informed consent or assent from potential trial participants or authorised surrogates, and how (see Item 32)	9
Consent or assent: ancillary studies	#26b	Additional consent provisions for collection and use of participant data and biological specimens in ancillary studies, if applicable	See Appendix 4
Confidentiality	#27	How personal information about potential and enrolled participants will be collected, shared, and maintained in order to protect confidentiality before, during, and after the trial	13
Declaration of interests	#28	Financial and other competing interests for principal investigators for the overall trial and each study site	20
Data access	#29	Statement of who will have access to the final trial dataset, and disclosure of contractual agreements that limit such access for investigators	19
Ancillary and post trial care	#30	Provisions, if any, for ancillary and post-trial care, and for compensation to those who suffer harm from trial participation	*Not applicable*
Dissemination policy: trial results	#31a	Plans for investigators and sponsor to communicate trial results to participants, healthcare professionals, the public, and other relevant groups (eg, via publication, reporting in results databases, or other data sharing arrangements), including any publication restrictions	19
Dissemination policy: authorship	#31b	Authorship eligibility guidelines and any intended use of professional writers	1
Dissemination policy: reproducible research	#31c	Plans, if any, for granting public access to the full protocol, participant-level dataset, and statistical code	*Not applicable*

For peer review only

Appendices

Informed consent materials	#32	Model consent form and other related documentation given to participants and authorised surrogates	Appendix 4
Biological specimens	#33	Plans for collection, laboratory evaluation, and storage of biological specimens for genetic or molecular analysis in the current trial and for future use in ancillary studies, if applicable	*Not applicable*

None The SPIRIT checklist is distributed under the terms of the Creative Commons Attribution License CC-BY-ND 3.0. This checklist can be completed online using https://www.goodreports.org/, a tool made by the EQUATOR Network in collaboration with Penelope.ai

For peer review only

BMJ Open

The Pre-BRA (Pre-pectoral Breast Reconstruction Evaluation) Feasibility Study: Protocol for a mixed-methods IDEAL 2a/2b prospective cohort study to determine the safety and effectiveness of pre-pectoral implant-based breast reconstruction

Journal:	*BMJ Open*
Manuscript ID	bmjopen-2019-033641.R1
Article Type:	Protocol
Date Submitted by the Author:	12-Nov-2019
Complete List of Authors:	Harvey, Kate; University of Bristol, Centre for Surgical Research, Bristol Medical School<br>Mills, Nicola; Bristol Medical School, Population Health Sciences<br>White, Paul; University of the West of England, Faculty of Environment and Technology<br>Holcombe, Christopher; Royal Liverpool and Broadgreen University Hospitals NHS Trust, Linda McCartney Breast Unit<br>Potter, Shelley; Bristol Medical School, Population Health Sciences; North Bristol NHS Trust, Bristol Breast Care Centre<br>Pre-BRA Study Steering Group, The
**Primary Subject Heading**:	Surgery
Secondary Subject Heading:	Health services research, Oncology
Keywords:	Breast surgery < SURGERY, Breast tumours < ONCOLOGY, PLASTIC & RECONSTRUCTIVE SURGERY

SCHOLARONE™
Manuscripts

The Pre-BRA (*Pre*-pectoral *B*reast *R*econstruction Ev*a*luation) Feasibility Study: Protocol for a mixed-methods IDEAL 2a/2b prospective cohort study to determine the safety and effectiveness of pre-pectoral implant-based breast reconstruction

Kate L Harvey[1], Nicola Mills[1], Paul White[2], Christopher Holcombe[3], Shelley Potter[1,4] on behalf of The Pre-BRA Feasibility Study Steering Group*

[1]National Institute for Health Research Bristol Biomedical Research Centre, University Hospitals Bristol NHS Foundation Trust and University of Bristol.

[2]Applied Statistics Group, University of the West of England, Bristol, UK

[3]Breast Unit, Royal Liverpool University Hospital, Prescot Street, Liverpool, L7 8XP, UK

[4]Bristol Breast Care Centre, North Bristol NHS Trust, Southmead Road, Bristol, BS10 5NB, UK

*Members of the Pre-BRA steering group are listed at the end of the manuscript and are PUBMED citable

For peer review only

Correspondence to: Kate Harvey, National Institute for Health Research Bristol Biomedical Research Centre, University Hospitals Bristol NHS Foundation Trust and University of Bristol., Whatley Road, Bristol, BS8 2PS

kate.harvey@bristol.ac.uk

Keywords: Pre-pectoral breast reconstruction, IDEAL, cohort study, mixed methods, safety

Word count: 3654

ABSTRACT

Introduction: Implant-based breast reconstruction (IBBR) is the most commonly-performed reconstructive technique worldwide. Sub-pectoral reconstruction with mesh is the current standard of care but new pre-pectoral techniques have recently been introduced. Pre-pectoral breast reconstruction (PPBR) may improve outcomes for patients but robust evaluation is required. Randomised clinical trials (RCTs) are ideally-needed but the short-term safety of PPBR is yet to be established; the technique and its indications are evolving and it has yet to be adopted by a sufficient number of surgeons for an RCT to be feasible.

Pre-BRA aims to determine the feasibility of using mixed-methods within an IDEAL 2a/2b study to explore the short-term safety of PPBR and determine when the technique is sufficiently stable for evaluation in a pragmatic RCT.

Methods and analysis: Pre-BRA is an IDEAL stage 2a/2b prospective multicentre cohort study with embedded qualitative research.

Consecutive patients electing to undergo immediate PPBR at participating centres will be invited to participate. Demographic, operative, oncology and complication data will be collected and patient-reported outcomes will be assessed at baseline, 3 and 18 months post-operatively. The primary safety endpoint will be implant loss at 3 months.

Surgeons performing PPBR will be asked to complete questionnaires regarding their practice and report any modifications made to the procedure or learning arising from complications via free-text response fields on electronic case-report forms. Semi-structured will explore surgeons' experiences in detail to identify emerging best practice. This will be fed back to participating surgeons to promote shared learning.

The Pre-BRA study will aim to recruit 341 patients from 30-40 UK centres over a 12-month period. Recruitment will commence Spring 2019.

For peer review only - http://bmjopen.bmj.com/site/about/guidelines.xhtml

Ethics and dissemination

The study has full ethical approval from OXFORD-B South Central Committee Ref:19/SC/0129. Results will be presented at national and international meetings and published in peer-reviewed journals.

Study registration: ISRCTN11898000

For peer review only

For peer review only - http://bmjopen.bmj.com/site/about/guidelines.xhtml

ARTICLE SUMMARY:

Strengths and Limitations of This Study

- Multicentre prospective cohort study which will generate high-quality data to support the safety of a novel approach to implant-based breast reconstruction prior to undertaking a definitive RCT.
- The study will assess the feasibility of using an innovative mixed-methods approach to promote shared surgical learning and establish when a novel surgical intervention is sufficiently stable for evaluation in the context of a RCT.
- The Pre-BRA Study is a single arm study without an in-built comparison group but the results will be compared safety data from recently published iBRA study and national quality standards.
- The feasibility of using mixed-methods to share learning and determine when the procedure has stabilised will depend on surgeon engagement and the feasibility of this approach is yet to be explored.

For peer review only

For peer review only - http://bmjopen.bmj.com/site/about/guidelines.xhtml

INTRODUCTION

Up to 40%[1] of the 55,000[2] women diagnosed with breast cancer each year in the UK will require a mastectomy and of these, approximately one in four will elect to have an immediate breast reconstruction[3] to improve their quality of life[4]. Implant-based breast reconstruction is the most commonly performed technique world-wide[5,6] and sub-pectoral implant-based breast reconstruction (IBBR) with biological or synthetic mesh has become the standard of care despite the lack of high-quality data to support the safety or effectiveness of the technique[7,8].

Recently, however, mesh-based techniques have evolved further with early studies[9] suggesting good results when fixed-volume implants are completely covered in a biological[10-12] or synthetic mesh[13-16] and placed on top of the muscle in a pre-pectoral position. This 'muscle-sparing' technique may result in less post-operative pain, more natural results and may prevent 'implant animation', the upwards movement of the implant seen when the pectoral muscle contracts[17].

While early results[18] of this pre-pectoral technique are promising in the hands of a few expert surgeons[10,11,13-15,19,20], historically subcutaneous implant placement without mesh, was abandoned by the reconstructive community due to high complication rates[21-24]. To date, few well-designed prospective studies have directly compared pre and sub-pectoral IBBR techniques[25] and evidence to support the proposed benefits of pre-pectoral implant placement is inconsistent[17,26,27]. There is therefore a need to robustly evaluate pre-pectoral implant reconstruction (PPBR) before it becomes standard practice[18]. Randomised clinical trials (RCTs) provide the best evidence, but the safety of PPBR has yet to be established; the technique and its indications are still evolving, and it has not yet been adopted by a sufficient number of surgeons in the UK for a randomised trial to be feasible. Preliminary work is therefore needed before definitive evaluation can be considered.

The IDEAL (Idea-Development-Exploration-Assessment-Long-term Study) Framework provides recommendations for the evaluation of a surgical innovation from first-in-man to long-

For peer review only - http://bmjopen.bmj.com/site/about/guidelines.xhtml

term study[28,29]. Phases 2a (Development)/2b (Exploration) focus on feasibility work for a definitive future trial including establishing the risks and benefits of the technique; the stability of the intervention, its indications and the adoption of the procedure by a wider group of surgeons from the initial innovators[30]. Whilst the scope and intention of IDEAL is clearly defined, the system is not well operationalised, and methods for the optimal conduct of early-phase surgical studies are unclear. Embedding qualitative methods within an IDEAL study may provide a novel way of enhancing shared learning within the surgical community to reduce learning curve effects and allow a new procedure to be introduced more safely and efficiently. Monitoring and sharing modifications may also provide an effective way of determining when the procedure is sufficiently stable for formal evaluation in the context of a definitive RCT.

METHODS AND ANALYSIS

Primary aim

The aim of Pre-BRA is to determine the feasibility of using mixed-methods within an IDEAL 2a/2b study to explore the short-term safety and effectiveness of pre-pectoral breast reconstruction and determine when it is sufficiently stable for formal evaluation in a definitive RCT.

Specific objectives will be

1. To establish the short-term safety (implant loss; infection; re-operation and readmission at 3 months) and effectiveness (patient-reported outcomes using BREAST-Q at 3 and 18 months) of pre-pectoral breast reconstruction compared with published national standards from the National Mastectomy and Breast Reconstruction Audit (NMBRA)[31] and iBRA[7] studies
2. To explore the feasibility of using a novel mixed-methods approach in an IDEAL 2a/2b study to promote shared learning and identify intervention stability including the acceptability of the methodology to surgeons and the most effective methods for providing feedback

For peer review only - http://bmjopen.bmj.com/site/about/guidelines.xhtml

3. To inform the feasibility, design and conduct of a future RCT including the numbers of patients at participating centres undergoing IBBR who are eligible for PPBR; types of meshes used and approach to concomitant interventions
4. To build capacity to deliver a future RCT by establishing a network of surgeons engaged in the need for evaluation who are able to perform pre-pectoral breast reconstruction to a high standard and who will participate in a future study

Study design

The Pre-BRA Study is a multicentre, IDEAL Phase 2a/2b prospective observational cohort study with embedded qualitative methods.

Patient and public involvement

This project is an extension of the iBRA study[7] which was designed with patient co-applicants. A patient group will be established to review and comment on the results. This patient group will input on the design of the future RCT, in particular, selection of the most appropriate primary outcome measure and timing of assessment.

Setting

All UK breast and plastic surgical centres currently performing pre-pectoral breast reconstruction using any technique will be invited to participate through the UK professional associations (Association of Breast Surgery [ABS], British Association of Plastic Reconstructive and Aesthetic Surgeons [BAPRAS]).

All surgeons currently performing PPBR will be eligible to participate in the study but those wishing to start performing the technique will be encouraged to follow the ABS/BAPARS guidelines regarding mentorship and training[32] prior to entering patients.

Participants

For peer review only - http://bmjopen.bmj.com/site/about/guidelines.xhtml

Inclusion criteria: Consecutive women aged 16 or over who require a mastectomy for breast cancer or risk-reduction who elect to undergo IBBR; are considered technically suitable for PPBR by their surgeon and consent to undergo the procedure are eligible to participate in the study. Patients undergoing PPBR will be informed that the procedure is innovative and that outcome data are limited as part of their discussion with the surgical team.

Exclusion criteria: Absolute and relative exclusion criteria will be used to reflect current practice and account for the variable experience of surgeons performing PPBR across the UK. Absolute exclusion criteria will be applied to all patients. Relative exclusion criteria can be applied flexibly according to the surgeon's experience with the procedure in conjunction with the patient's consent following discussion that the risk of complications may be increased in this setting.

Absolute exclusion criteria will be:

i. Patients assessed as having insufficient soft tissue coverage for PPBR by the operating surgeon
ii. Patients having revisional or delayed breast reconstruction
iii. Patients unable or unwilling to provide informed consent.

Relative exclusion criteria reflect ABS/BAPRAS guidelines for mesh-assisted breast reconstruction[32] and include

i. Current smokers or recent ex-smokers (<6 weeks)
ii. Previous breast/chest wall radiotherapy
iii. Body mass index (BMI) of greater than 30
iv. Reconstruction with an implant of more than 600cc
v. Patients anticipated to require post mastectomy radiotherapy
vi. Poorly-controlled diabetes

For peer review only - http://bmjopen.bmj.com/site/about/guidelines.xhtml

Participant identification, screening and recruitment

All female patients aged 16 or over undergoing mastectomy for breast cancer or risk-reduction who elect to undergo immediate IBBR will be screened to inform the feasibility of a future trial comparing different approaches to IBBR.

Participant screening

Potential participants will be identified from clinics and multidisciplinary meetings by the clinical and research teams.

A comprehensive screening log will be maintained at participating sites of all women who elect to undergo immediate IBBR under the care of participating surgeons to determine the proportion of patients who:

i. Are considered technically suitable for pre-pectoral technique by the operating surgeon.
ii. For those patients who are considered not suitable for PPBR – with reasons for this (e.g. relative exclusion criteria: smoker; previous RT etc. or other reason).
iii. For patients considered technically suitable for PPBR, the proportion who accept the technique.
iv. For those patients accepting PPBR, the proportion who consent to study participation

Patient recruitment

Patients who elect to undergo PPBR will be given a patient information sheet (PIS) explaining the study. They will be seen at a follow up appointment by a member of the clinical or research team and asked to sign a consent form prior to participating in the study. (Appendix 1) Patients will be allocated a study ID number.

For peer review only - http://bmjopen.bmj.com/site/about/guidelines.xhtml

Patients will be given a date for surgery as per unit protocol and undergo pre-operative assessment. They will be required to complete baseline patient-reported outcome questionnaires (BREAST-Q) and have pre-operative photographs as per unit policy prior to surgery.

Study procedures

All patients in whom pre-pectoral reconstruction with or without mesh is planned will be eligible for inclusion in the study. All meshes must be licensed for use in the UK and have a CE mark. Composite pre-pectoral reconstructions using dermal sling and mesh or two different types of mesh will be permitted and the details of products used will be recorded. Reconstructions using full dermal sling coverage will also be included.

Standardisation of surgical technique and procedure fidelity

All patients will undergo a skin or nipple-preserving mastectomy followed by an immediate implant-based breast reconstruction.

Participating surgeons will undertake the procedure as per their standard practice. Mesh choice (biological or synthetic and the product used) and implant selection (fixed-volume; adjustable implants or tissue expanders) will be as per surgeon preference. All patients will be planned for a pre-pectoral reconstruction prior to surgery.

The following steps of the PPBR procedure will be considered mandatory, prohibited and discretionary according to the typology described by Blencowe et al.[33]

- **Mandatory** – Insertion of a tissue expander/adjustable implant or fixed-volume implant
- **Prohibited** –Raising the pectoralis muscle
- **Discretionary** – All other steps of the procedure

If the surgeon considers that pre-pectoral reconstruction is not possible at the time of surgery or modifies the planned procedure (e.g. inserts tissue expander rather than fixed-volume

For peer review only - http://bmjopen.bmj.com/site/about/guidelines.xhtml

implant), this will be recorded on the case report form (CRF) with reasons for the modification (e.g. thin skin-flaps). Details of the alternative procedure performed (e.g. sub-pectoral reconstruction) will be collected. Surgeons will also be encouraged to record any learning arising from the case.

Strategies to minimise infection (e.g. use of laminar flow, cavity irrigation, glove change) will be as per local practice but participating centres will be encouraged to adhere to published best practice guidelines[32,34] and use the evidence-based Manchester checklist[35].

Use of drains and other concomitant interventions (e.g. antibiotics, dressings) will be permitted as per local practice.

Deviations from planned pre-pectoral reconstruction

As detailed above, surgeons will be asked to report the following via the CRF:

a. Patients in whom PPBR was planned but had to be abandoned completely (i.e. another form of reconstruction or no reconstruction performed)

    i. Reasons why pre-pectoral approach was abandoned
    ii. Procedure performed instead
    iii. Any reflections or learning regarding the case

b. Patients in whom the planned procedure was modified (but pre-pectoral reconstruction still performed)

    i. Details of the modification made (e.g. insertion of tissue expander rather than fixed volume implant)
    ii. Reasons for the modification (e.g. thin skin flaps)
    iii. Any reflections or learning regarding the case

For peer review only - http://bmjopen.bmj.com/site/about/guidelines.xhtml
For peer review only

Outcome measures

Safety and effectiveness outcomes

The primary outcome for the pre-pectoral safety study will be implant loss at 3 months defined as the removal of the implant or tissue-expander without replacement as a result of a complication. This is consistent with the definition used in the iBRA study[7]. Secondary safety and effectiveness outcomes will be:

i. Post-operative pain assessed by patient self-report using a visual analogue scale at 24 hours, 1 week, 2 weeks and 3 months
ii. Complications requiring readmission and/or re-operation at 3 months
iii. Infection defined as i) minor – requiring treatment with oral antibiotics ii) major requiring admission for intravenous antibiotics and/or surgical debridement
iv. Quality of life including satisfaction with breasts; physical well-being; psychosocial well-being and animation assessed using the validated BREAST-Q[36] questionnaire at 3 and 18 months following surgery.
v. Further surgery for complications or cosmesis at 18 months

Feasibility outcomes

Surgeon engagement will be used to assess the feasibility of using mixed-methods to explore learning and intervention stability in the study. At study entry, the number of surgeons completing the surgeon questionnaire; the number of surgeons consenting to be contacted for interview and the number of surgeons interviewed will be compared. For modifications/learning arising from complications, the numbers of events (modifications and complications) reported will be compared with numbers of free-text responses entered; the number of surgeons consenting to interview and the actual number interviewed. The content of the free-text responses and interviews will also be assessed.

For peer review only - http://bmjopen.bmj.com/site/about/guidelines.xhtml

Screening logs will be reviewed to determine the relative proportions of patients electing to undergo IBBR who are i.) considered technically suitable for pre-pectoral reconstruction and ii) elect to undergo surgery to determine if a full-scale pragmatic RCT is feasible.

Data collection

Clinical data

Demographic, comorbidity and operative data will be collected for each participant via a standardised electronic case report form (CRF) hosted on REDCap[37] (Appendix 2).

Complications and oncological data will be collected at 30 days and 3 months by clinical or case-note review according to local follow up policies. Details of any additional surgery required for complications or cosmesis will be collected at 18 months by case note review. No additional clinic visits will be required for the study. All complications will be defined a priori using standardised definitions as per previous studies[7,38] (Appendix 3***)***

Patient-reported outcomes

Participants will be asked to complete the pre-operative breast reconstruction BREAST-Q questionnaire prior to surgery.

Post-operative pain scores will be collected by patient self-report using an electronic visual analogue scale at 24 hours, 1 week, 2 weeks and 3 months following surgery.

Patient satisfaction with breasts, physical well-being, psychosocial well-being, satisfaction with implants and patient-reported assessment of animation will be assessed at 3 and 18 months using the validated BREAST-Q questionnaire[36]. A reminder will be sent after one month if no response is received. Patients will be encouraged to complete on-line PROMs but paper versions will be made available to patients unable or unwilling to use electronic versions.

For peer review only - http://bmjopen.bmj.com/site/about/guidelines.xhtml

Shared learning and intervention stability

Best practice and opportunities for shared learning among participating surgeons will occur at the following points in the study.

i. Study entry

ii. As a result of procedure modifications or complications

Surgeons electing to participate in the study will be sent a surgeon information sheet outlining the proposed shared learning study and what participation may involve. Surgeons will be able to recruit patients to Pre-BRA without contributing to shared learning but full participation will be encouraged by the steering group.

At each time point a combination of free-text responses on standardised CRFs and focussed semi-structured qualitative interviews will be to explore group learning and intervention stability.

i. Study entry

Phase 1 - Surgeon questionnaire

Prior to commencing patient recruitment, participating surgeons will be asked to complete a surgeon questionnaire. The questionnaire (appendix 4) will explore techniques and products used; experience; approaches to concomitant interventions and patient selection criteria. The questionnaire will include consent to be contacted regarding a brief (20 min) semi-structured qualitative interview exploring their practice in more detail.

Phase 2 – Semi-structured study entry interviews

Sampling and data collection

For peer review only - http://bmjopen.bmj.com/site/about/guidelines.xhtml

A purposive sample of surgeons consenting to interview will be selected based on techniques and products used; experience; geographical location and differing approaches to concomitant interventions and patient selection.

Interviews will be organised at a time convenient to the participating surgeons. All interviews will be conducted by telephone using a semi-structured topic guide developed based on the literature and clinical expertise to explore issues surrounding current practice. All interviews will be audio-recorded and transcribed verbatim. Interview participants will be asked to give verbal consent to study participation and audio-recording prior to commencing the interview. The topic guide will be modified iteratively as the study progresses to allow new themes to emerge. Sampling, data collection and analysis will be conducted concurrently and iteratively until data saturation is achieved and no new themes emerge from the data.

ii. As a result of procedure modifications and learning from complications

Phase 1 – Case report forms

Surgeons will be asked to report (yes/no) whether i) a modification to the planned procedure was made at the time of the operation and ii) any learning (e.g. changes to future surgical technique) occurred as a result of a complication at 3 months. If modifications or learning as a result of complications are reported, surgeons will be asked to provide details in a free text response box on the electronic CRF and to indicate whether they would be willing to be contacted to participate in a brief telephone interview to explore this in more detail.

Phase 2 - Semi-structured surgeon modification/complication interviews

Sampling and data collection

Surgeons providing consent will be contacted to arrange a brief semi-structured qualitative interview. Interviews will be conducted as per study entry interviews outlined above but will

For peer review only - http://bmjopen.bmj.com/site/about/guidelines.xhtml

focus specifically on the modification/learning arising from the complication including details, rationale and outcome.

The feasibility of this approach is unknown so initially, it is planned that all surgeons reporting procedure modifications/learning from complications and providing consent will be interviewed. As the study progresses, depending on the number and content of the CRF reports, all consenting surgeons may continue to be interviewed or, it may be necessary to purposively sample consenting surgeons based on the details of the modification/learning reported in the CRF to explore new and emerging themes and allow the details of the modifications to be explored. Data collection will continue until no new learning or modifications emerge from the data.

Data analysis and sample size

Pre-pectoral safety and effectiveness

Sample Size

A single-arm 2-stage design will be used to assess the safety of PPBR based on the primary outcome of implant loss rate at 3 months. It is expected that the implant loss rate will be 9% or lower based on findings from the recent iBRA study[7]. 15% or greater will be deemed unacceptably high.

A sample size of 310 patients would result in a two-sided 95.0 % confidence interval for a single proportion, assumed to be 0.10, with a width equal to 0.070. Allowing for a 10% loss to follow-up at 3 months at least 341 patients will be recruited to inform a future trial with implant loss as the primary outcome.

Based on data from the iBRA study, the number of pre-pectoral reconstructions performed at each site is likely to be small (4-40 per year)[7]. We will therefore aim to recruit from between 20 and 30 sites over a period of 12 months.

For peer review only - http://bmjopen.bmj.com/site/about/guidelines.xhtml

Statistical analysis

Simple summary statistics will be calculated to describe demographic, procedure, process and outcome data. Categorical data will be summarised by counts and percentages and continuous data by median, interquartile range and range.

We will establish the proportion and 95% confidence intervals of patients experiencing implant loss and other key safety outcomes (re-admission for complications, re-operation and infection) at three months and compare these with findings with those reported in the iBRA study[7] .

An exploratory risk factor analysis will be conducted to explore the impact of type of mesh and known risk factors for complications including smoking; BMI and radiotherapy if sufficient numbers of patients are recruited to the study.

Planned interim analysis

A planned interim analysis will be performed when 155 patients have been recruited to the study and followed-up for a minimum of 3 months. The report will be reviewed by the study steering group and the results will be made available to the study sponsor.

If the overall implant loss rate at 3 months following surgery is 15% or more, the study will be stopped.

Shared learning and intervention stability feasibility study

Analysis of the free-text responses will be an ongoing and iterative process commencing soon after the study begins and will inform sampling for the semi-structured interview phase of the study. Text will be assigned codes and analysed using the constant comparative technique of grounded theory[39]. A combination of thematic and content analysis will be used to identify common themes[40,41]. These will inform the topic guide for the interviews and the feedback provided to study participants.

For peer review only - http://bmjopen.bmj.com/site/about/guidelines.xhtml

Sampling, data collection and analysis will be undertaken concurrently and iteratively until no new themes emerge from the data.

Dissemination of best practice and shared learning

The learning and modifications emerging from the CRFs and interviews will be consolidated and reviewed at regular intervals by the study team. Any common themes will be shared with participating surgeons using a combination of methods. These will include a 'tips and tricks' section on the study website; e-mail updates; study newsletters and potentially social media channels (e.g. 'You Tube'). The acceptability and value of the different approaches to sharing learning will be discussed with individual surgeons at interview and the wider steering group. Metrics such as number of views/hits for online/social media resources will used to provide additional information regarding engagement if appropriate.

Design of the main study

If PPBR is considered safe and data from the screening logs suggests sufficient numbers of PPBR are being performed at participating centres, a consensus meeting with key participating surgeons, steering group members, patients and methodologists will be held to agree the final design for a large-scale pragmatic RCT comparing pre-pectoral and sub-pectoral implant-based breast reconstruction.

ETHICS AND DISSEMINATION

Full ethical approval has been obtained for the study (NRES OXFORD-B South Central Committee Ref:19/SC/0129. IRAS ID: 255421) and written informed consent will be obtained for study participation. All potential study participants will be informed that pre-pectoral reconstruction is a new technique and that outcome data is limited. An interim analysis is planned and if implant loss rates exceed 15%, the study will be stopped.

For peer review only

Results will be analysed and presented at national and international meetings and published in peer-reviewed journals. Findings will be fed back to the surgical community to promote engagement and recruitment to a future RCT.

AUTHORS' CONTRIBUTIONS

SP and CH conceived the study. KH, SP, NM, CH contributed to the study design. PW provided statistical support. SP, CH and KH secured funding for the project. KH wrote the paper, SP critically revised the manuscript. All authors read, critically revised and approved the manuscript before submission.

Members of the Pre-BRA Feasibility Study Steering group in alphabetical order are:

Peter Barry, Simon Cawthorn, Matthew Gardiner, Gareth Irwin, Cliona Kirwan, Mairead McKenzie, Shireen McKenzie, Rachel O'Connell, Georgette Oni, Tim Rattay, Pankaj Roy, Joanna Skillman, Soni Soumian, Raghavan Vidya, Lisa Whisker and Samantha Williams.

STUDY SPONSOR

The University of Bristol.

Research Governance Officer: Ms Anna Brooke: research-governance@bristol.ac.uk

DATA ACCESS STATEMENT

Data are available upon reasonable request. De-identified participant data from the safety study will be available from the senior author after completion of the study and planned analyses following review by the study steering group. Reuse will be permitted with consent of the study steering group. No additional information will be available. Data from the surgeon interviews will not be made available due to the sensitive nature of the information collected and the ability to identify study participants.

FUNDING STATEMENT

For peer review only - http://bmjopen.bmj.com/site/about/guidelines.xhtml

This work was supported by a One Year Royal College of Surgeons Blond McIndoe Foundation Research Fellowship, the Association of Breast Surgery and the NIHR Biomedical Research Centre at University Hospitals Bristol NHS Foundation Trust and the University of Bristol. SP is an NIHR Clinician Scientist (CS-2016-16-019). The views expressed are those of the authors and not necessarily those of the NIHR or the Department of Health and Social Care.

COMPETING INTERESTS STATEMENT

The authors have no conflict of interest to declare.

REFERENCES

1. Matala CM, McIntosh SA, Purushotham AD. Immediate breast reconstruction after mastectomy for cancer. *Br J Surg* 2000; **87**: 1455-72.

2. Cancer Research UK. https://www.cancerresearchuk.org/health-professional/cancer-statistics/statistics-by-cancer-type/breast-cancer#heading-Zero. 2018 (accessed 18th November 2018.

3. Jeevan R, Mennie JC, Mohanna PN, O'Donoghue JM, Rainsbury RM, Cromwell DA. National trends and regional variation in immediate breast reconstruction rates. *Br J Surg* 2016; **103**(9): 1147-56.

4. Harcourt D, Rumsey N, Ambler N, et al. The psychological effect of mastectomy with or without breast reconstruction: A prospective, multicentre study. *Plastic & Reconstructive Surgery* 2003; **111**(3): 1060-8.

5. Mennie JC, Mohanna PN, O'Donoghue JM, Rainsbury R, Cromwell DA. National trends in immediate and delayed post-mastectomy reconstruction procedures in England: A seven-year population-based cohort study. *Eur J Surg Oncol* 2017; **43**(1): 52-61.

6. Albornoz CR, Bach PB, Mehrara BJ, et al. A paradigm shift in U.S. Breast reconstruction: increasing implant rates. *Plast Reconstr Surg* 2013; **131**(1): 15-23.

For peer review only - http://bmjopen.bmj.com/site/about/guidelines.xhtml

7. Potter S, Conroy EJ, Cutress RI, et al. Short-term safety outcomes of mastectomy and immediate implant-based breast reconstruction with and without mesh (iBRA): a multicentre, prospective cohort study. *The Lancet Oncology* 2019; **20**(2): 254-66.

8. Potter S, Browning D, Savovic J, Holcombe C, Blazeby JM. Systematic review and critical appraisal of the impact of acellular dermal matrix use on the outcomes of implant-based breast reconstruction. *British Journal of Surgery* 2015; **102**(9): 1010-25.

9. Salibian AA, Frey JD, Choi M, Karp NS. Subcutaneous Implant-based Breast Reconstruction with Acellular Dermal Matrix/Mesh: A Systematic Review. *Plastic and Reconstructive Surgery – Global Open* 2016; **4**(11): e1139.

10. Berna G, Cawthorn SJ, Papaccio G, Balestrieri N. Evaluation of a novel breast reconstruction technique using the Braxon® acellular dermal matrix: a new muscle-sparing breast reconstruction. *ANZ Journal of Surgery* 2014: n/a-n/a.

11. Reitsamer R, Peintinger F. Prepectoral implant placement and complete coverage with porcine acellular dermal matrix: A new technique for direct-to-implant breast reconstruction after nipple-sparing mastectomy. *Journal of Plastic, Reconstructive & Aesthetic Surgery* 2015; **68**(2): 162-7.

12. Zhu L, Mohan AT, Abdelsattar JM, et al. Comparison of subcutaneous versus submuscular expander placement in the first stage of immediate breast reconstruction. *Journal of Plastic, Reconstructive & Aesthetic Surgery* 2016; **69**(4): e77-e86.

13. Casella D, Bernini M, Bencini L, et al. TiLoop® Bra mesh used for immediate breast reconstruction: comparison of retropectoral and subcutaneous implant placement in a prospective single-institution series. *European Journal of Plastic Surgery* 2014; **37**(11): 599-604.

14. Casella D, Calabrese C, Bianchi S, Meattini I, Bernini M. Subcutaneous Tissue Expander Placement with Synthetic Titanium-Coated Mesh in Breast Reconstruction: Long-term Results. *Plastic and Reconstructive Surgery – Global Open* 2015; **3**(12): e577.

For peer review only

For peer review only - http://bmjopen.bmj.com/site/about/guidelines.xhtml

15. Bernini M, Calabrese C, Cecconi L, et al. Subcutaneous Direct-to-Implant Breast Reconstruction: Surgical, Functional, and Aesthetic Results after Long-Term Follow-Up. *Plastic and Reconstructive Surgery – Global Open* 2015; **3**(12): e574.

16. Kobraei EM, Cauley R, Gadd M, Austen WGJ, Liao EC. Avoiding Breast Animation Deformity with Pectoralis-Sparing Subcutaneous Direct-to-Implant Breast Reconstruction. *Plastic and Reconstructive Surgery – Global Open* 2016; **4**(5): e708.

17. Cattelani L, Polotto S, Arcuri MF, Pedrazzi G, Linguadoca C, Bonati E. One-Step Prepectoral Breast Reconstruction With Dermal Matrix-Covered Implant Compared to Submuscular Implantation: Functional and Cost Evaluation. *Clinical breast cancer* 2018; **18**(4): e703-e11.

18. Tasoulis MK, Iqbal FM, Cawthorn S, MacNeill F, Vidya R. Subcutaneous implant breast reconstruction: Time to reconsider? *European Journal of Surgical Oncology (EJSO)* 2017.

19. Becker H, Lind JGI, Hopkins EG. Immediate Implant-based Prepectoral Breast Reconstruction Using a Vertical Incision. *Plastic and Reconstructive Surgery – Global Open* 2015; **3**(6): e412.

20. Vidya R, Masià J, Cawthorn S, et al. Evaluation of the effectiveness of the prepectoral breast reconstruction with Braxon dermal matrix: First multicenter European report on 100 cases. *The Breast Journal*: n/a-n/a.

21. SNYDERMAN RK, GUTHRIE RH. RECONSTRUCTION OF THE FEMALE BREAST FOLLOWING RADICAL MASTECTOMY. *Plastic and Reconstructive Surgery* 1971; **47**(6): 565-7.

22. Gruber RP, Kahn RA, Lash H, Maser MR, Apfelberg DB, Laub DR. Breast Reconstruction Following Mastectomy: A Comparison of Submuscular and Subcutaneous Techniques. *Plastic & Reconstructive Surgery* 1981; **67**(3): 312-7.

23. Rebowe RE, Allred LJ, Nahabedian MY. The Evolution from Subcutaneous to Prepectoral Prosthetic Breast Reconstruction. *Plastic and reconstructive surgery Global open* 2018; **6**(6): e1797.

For peer review only

For peer review only - http://bmjopen.bmj.com/site/about/guidelines.xhtml

24. Wagner RD, Braun TL, Zhu H, Winocour S. A systematic review of complications in prepectoral breast reconstruction. *Journal of Plastic, Reconstructive & Aesthetic Surgery* 2019; **72**(7): 1051-9.

25. Li L, Su Y, Xiu B, et al. Comparison of prepectoral and subpectoral breast reconstruction after mastectomies: A systematic review and meta analysis. *European Journal of Surgical Oncology* 2019.

26. Baker BG, Irri R, MacCallum V, Chattopadhyay R, Murphy J, Harvey JR. A Prospective Comparison of Short-Term Outcomes of Subpectoral and Prepectoral Strattice-Based Immediate Breast Reconstruction. *Plast Reconstr Surg* 2018; **141**(5): 1077-84.

27. Bettinger LN, Waters LM, Reese SW, Kutner SE, Jacobs DI. Comparative Study of Prepectoral and Subpectoral Expander-Based Breast Reconstruction and Clavien IIIb Score Outcomes. *Plastic and reconstructive surgery Global open* 2017; **5**(7): e1433.

28. Ergina PL, Cook JA, Blazeby JM, et al. Challenges in evaluating surgical innovation. *Lancet* 2009; **374**: 1097-104.

29. Pennell CP, Hirst AD, Campbell WB, et al. Practical guide to the Idea, Development and Exploration stages of the IDEAL Framework and Recommendations. *British Journal of Surgery* 2016; **103**(5): 607-15.

30. Hirst A, Phillippou Y, Blazeby J, et al. No Surgical Innovation without Evaluation: Evolution and Further Development of the IDEAL Framework and Recommendations. *Annals of Surgery* 2019; **269**(2): 211-20.

31. Jeevan R, Cromwell DA, Browne JP, et al. Findings of a national comparative audit of mastectomy and breast reconstruction surgery in England. *J Plast Reconstr Aesthet Surg* 2014; **67**(10): 1333-44.

32. Martin L, O'Donoghue JM, Horgan K, et al. Acellular dermal matrix (ADM) assisted breast reconstruction procedures: joint guidelines from the Association of Breast Surgery and the British Association of Plastic, Reconstructive and Aesthetic Surgeons. *European Journal of Surgical Oncology* 2013; **39**(5): 425-9.

For peer review only

For peer review only - http://bmjopen.bmj.com/site/about/guidelines.xhtml

33. Blencowe NS, Mills N, Cook JA, et al. Standardizing and monitoring the delivery of surgical interventions in randomized clinical trials. *British Journal of Surgery* 2016; **103**(10): 1377-84.

34. Rainsbury D, Willett A. Oncoplastic breast reconstruction: guidelines for best practice. UK, 2012.

35. Barr SP, Topps AR, Barnes NL, et al. Infection prevention in breast implant surgery - A review of the surgical evidence, guidelines and a checklist. *Eur J Surg Oncol* 2016; **42**(5): 591-603.

36. Pusic AL, Klassen A, Scott AF, Klok JA, ordeiro P G, Cano S. Development of a new patient-reported outcome measure for breast surgery: the BREAST-Q. *Plastic and Reconstructive Surgery* 2009; **124**(2): 345-53.

37. Harris PA, Taylor R, Thielke R, Payne J, Gonzalez N, Conde JG. Research electronic data capture (REDCap)--a metadata-driven methodology and workflow process for providing translational research informatics support. *J Biomed Inform* 2009; **42**(2): 377-81.

38. O'Connell RL, Rattay T, Dave RV, et al. The impact of immediate breast reconstruction on the time to delivery of adjuvant therapy: the iBRA-2 study. *Br J Cancer* 2019.

39. Glaser B, Strauss AL. The Discovery of Grounded Theory. Chicago: Adeline; 1967.

40. Braun V, Clarke V. Using thematic analysis in psychology. *Qualitative Research in Psychology* 2006; **3**(2): 77-101.

41. Krippendorff K. Values, modes and domains of inquiry into communication. *The Journal of communication* 1969; **19**(2): 105-33.

For peer review only

For peer review only - http://bmjopen.bmj.com/site/about/guidelines.xhtml

The Pre-BRA (Pre-pectoral Breast Reconstruction Evaluation) Feasibility Study: Protocol
KL Harvey, N Mills, P White, C Holcombe, S Potter
Appendix 1.

Local Trust Logo here

The Pre-BRA Feasibility Study
PATIENT CONSENT FORM

Please initial box

1	I confirm that I have read and understand the information sheet dated 28/03/2019 (version 2.0) for the above study. I have had the opportunity to consider the information, ask questions and have had these answered satisfactorily.	☐
2	I understand that my participation is voluntary and that I am free to withdraw at any time without giving any reason, without my medical care or legal rights being affected.	☐
3	I understand that relevant sections of my medical notes and data collected during the study may be looked at by individuals from regulatory authorities, the study research team or from the NHS Trust, where it is relevant to my taking part in this research. I give permission for these individuals to have access to my records.	☐
4	I agree that the study team may retain personally identifiable data collected during the study which could be used to directly support future research studies such as an extension to the Pre-Bra Study. For example, the study team may wish to contact me with further questionnaires, or to use identifiers such as my individual NHS number to interrogate national databases.	☐
5	I agree that data collected during the study may be retained by the study team for up to 10 years after the study has closed, in order to support future research studies.	☐
6	I agree to complete patient questionnaires for use in the study	☐
7	I agree to take part in the above study	☐
8	I would like to be sent a summary of the study's findings when the research is complete: Yes ☐ No ☐<br>I have supplied my email address for that purpose.<br>Email address: ____________________	☐

Name of Patient	Date	Signature
Researcher Taking Consent	Date	Signature

One copy of the signed consent form should be given to the participant and one copy retained by the researcher.

For peer review only - http://bmjopen.bmj.com/site/about/guidelines.xhtml

The Pre-BRA (Pre-pectoral Breast Reconstruction Evaluation) Feasibility Study: Protocol
KL Harvey, N Mills, P White, C Holcombe, S Potter
Appendix 2.

CASE REPORT FORM

**Unit Name**	

**REDCap Record ID***										

Record ID = * Unit code+### e.g. NBT001, NBT002 etc.

**Participant email address**	

For peer review only

The Pre-BRA (Pre-pectoral Breast Reconstruction Evaluation) Feasibility Study: Protocol
KL Harvey, N Mills, P White, C Holcombe, S Potter
Appendix 2.

Pre-Op and Operative Data

Pre-BRA

For peer review only

For peer review only - http://bmjopen.bmj.com/site/about/guidelines.xhtml

The Pre-BRA (Pre-pectoral Breast Reconstruction Evaluation) Feasibility Study: Protocol
KL Harvey, N Mills, P White, C Holcombe, S Potter
Appendix 2.

Participant Data

Participant withdrawn? Yes ☐ No ☐

(*Only tick 'yes' if the patient no longer wishes to participate in the study)*

Participant questionnaire completion: *(includes BREAST-Q PROMS and Pain Scores)*

Baseline* on paper, follow-ups** online ☐

Everything online ☐

Everything on paper - *PLEASE INFORM CENTRAL STUDY TEAM* ☐

*Baseline defined as: baseline (pre-operative) BREAST-Q PROMS and 24-hours post-op pain score.
**Follow-ups defined as: 3-month and 18-month BREAST-Q PROMS and 1 week, 2 weeks and 3-months pain scores

IF the patient has chosen to complete baseline PROMS on paper and follow-ups online, please offer the participant:

i. a PAPER copy of the Pre-Operative BREAST-Q AND
ii. ask them to record 24-hour post-op pain score in person (/over the telephone if discharged)
(These results need to be transcribed into REDCap by a member of the research team)
iii. All other PROMS will be requested via email direct to the patient

IF the patient has chosen to complete all online PLEASE NOTE:

The patient's record including email address and date of surgery **must** be saved on REDCap on or before the day of surgery in order to generate electronic baseline (pre-operative) BREAST-Q and 24hours post-op pain score requests.

IF the patient has chosen to complete everything on paper, please offer the participant:

i. a PAPER copy of the Pre-Operative BREAST-Q for completion prior to surgery
ii. PAPER copies of the Post-operative BREAST-Q for completion at 3 and 18 months post-operatively (could be sent by post) AND
iii. Ask them to record 24 hour post-op pain score in person (/over the telephone if discharged)
iv. 1 week, 2 weeks and 3 months post-op pain scores can be collected by telephone call from a member of the research team.
(These results need to be transcribed into REDCap by a member of the research team)

For peer review only - http://bmjopen.bmj.com/site/about/guidelines.xhtml

Pre-BRA Section 1 - Pre-operative data

Co-morbidity data

Patient age (in years) on entering the study ………………….. **Years**

Planned* **date of primary mastectomy and reconstruction**
*(*Required to generate automatic electronic BREAST-Q PROMS and post-operative pain score requests via REDCap)*

………/………../………(DD/MM/YYYY)

Laterality of planned surgery?

Right breast ☐ Left Breast ☐ Bilateral ☐

Indication for RIGHT mastectomy and planned reconstruction? (leave blank if n/a)

Risk reducing ☐ Malignancy ☐

Indication for LEFT mastectomy and planned reconstruction? (leave blank if n/a)

Risk reducing ☐ Malignancy ☐

Answer ONLY IF indication for surgery is malignancy: (leave blank if n/a)

RIGHT BREAST:

First operation for cancer ☐
Completion mastectomy* ☐
Local recurrence ☐

LEFT BREAST:

First operation for cancer ☐
Completion mastectomy* ☐
Local recurrence ☐

** i.e. Mastectomy performed following unsuccessful breast conservation surgery for example wide local excision (WLE) or therapeutic mammoplasty (TM)*

Medical co-morbidities?

Yes ☐ No ☐

Answer ONLY IF medical comorbidities present: (please tick all relevant)

Diabetes ☐
Asthma/ COPD ☐
Ischaemic heart disease ☐
Connective tissue disease ☐
Current steroid therapy ☐
Anticoagulant therapy (prior to surgery) ☐
Other co-morbidity ☐

If other, please supply details: ……………………………………………………………..

For peer review only

The Pre-BRA (Pre-pectoral Breast Reconstruction Evaluation) Feasibility Study: Protocol
KL Harvey, N Mills, P White, C Holcombe, S Potter
Appendix 2.

Prior and neo-adjuvant treatments

Previous radiotherapy to ipsilateral breast or chest wall
(MEETS RELATIVE EXCLUSION CRITERIA)

Yes ☐ No ☐

Neo-adjuvant chemotherapy prior to mastectomy and reconstruction?

Yes ☐ No ☐

Previous surgery to ipsilateral breast?

No ☐ (Please go to Section 2))

Yes ☐

(REDCap™ will open the following options, please tick all relevant and indicate date of surgery:)

Previous axillary surgery ☐ Year................................

Wide local excision ☐ Year...............................

Augmentation ☐ Year................................

Reduction ☐ Year................................

Other ☐ Year................................

Pre-BRA Section 2 - Assessment of suitability for pre-pectoral breast reconstruction

Smoking status **(RELATIVE EXCLUSION if current smoker)**

Non-smoker ☐

Current smoker ☐

Nicotine replacement/ vaping with nicotine ☐

Recent ex-smoker (i.e. stopped at point of diagnosis) ☐

Grade of ptosis

0 No ptosis ☐

1 Mild - Nipple at level of IMF and above most of lower breast tissue ☐

2 Moderate - Nipple below IMF, but higher than most of the breast tissue ☐

3 Advanced - Nipple below IMF and at level of maximal breast projection ☐

4 Severe - Nipple far below IMF and points towards the floor ☐

Predicted implant vol (cc) ....

For peer review only - http://bmjopen.bmj.com/site/about/guidelines.xhtml

The Pre-BRA (Pre-pectoral Breast Reconstruction Evaluation) Feasibility Study: Protocol
KL Harvey, N Mills, P White, C Holcombe, S Potter
Appendix 2.

(>600cc fixed volume **MEETS RELATIVE EXCLUSION CRITERIA**)

Anticipated adjuvant therapy **(RELATIVE EXCLUSION if PMRT is anticipated)**

Post-mastectomy radiotherapy?

Not required (risk reduction) ☐

No, previously received ☐

Unlikely (e.g. DCIS only) ☐

Possible ☐

Probable (positive nodes, large tumour) ☐

Body Mass Index

Patient height (cm) …………………………. Patient weight (kg) …………………………….

BMI (REDCap™ calculates) ……………………. **(RELATIVE EXCLUSION if >30)**

Manual BMI entry *(only answer if height/ weight values unknown)* ……………………….

Suitability for pre-pectoral reconstruction

RELATIVE CONTRA-INDICATIONS:

- Previous breast/ chest wall radiotherapy or mantle radiotherapy
- Anticipated post-mastectomy radiotherapy
- BMI >30
- Current smoker (or nicotine dependent)
- Implant volume >600cc

What is your clinical impression of this patient's risk of experiencing a post-operative complication?

They are at low risk ☐ (Defined as no relative contraindications)

They are at moderate risk ☐ (Defined as 1 relative contra-indication)

They are at high risk ☐ (Defined as >1 relative contraindication)

They are high risk for another reason ☐

Please supply details if you have specified another reason

……………………………………………………………………………………………………

……………………………………………………………………………………………………

……………………………………………………………………………………………………

Are any additional surgical procedures planned at a later date? *(please select all that apply)*

For peer review only - http://bmjopen.bmj.com/site/about/guidelines.xhtml

The Pre-BRA (Pre-pectoral Breast Reconstruction Evaluation) Feasibility Study: Protocol
KL Harvey, N Mills, P White, C Holcombe, S Potter
Appendix 2.

Lipo-modelling ☐

Nipple reconstruction ☐

Contralateral symmetrising Reduction/ Mastopexy/ Augmentation ☐

No further procedures planned ☐

Pre-BRA Section 3 - Modifications to Usual Patient Selection Criteria

Have you modified your usual patient selection criteria in order to offer this patient a pre-pectoral implant reconstruction?

Yes ☐ No ☐

If you have answered "yes", please explain in what way: ....

....

....

....

....

Note: The study team are very interested in speaking to surgeons to explore any modifications made to their usual patient selection criteria.

Does the operating surgeon consent to be contacted for a brief telephone interview?

Yes ☐ No ☐

Please supply the email address of the operating surgeon if yes:

....

2.12 FINAL STUDY ENTRY CHECKLIST

**2.8.1 Meets inclusion/exclusion criteria for the study**	**Yes/No**
**2.8.2 Written consent to participate in the study**	**Yes/No**
**2.8.3 Pre-op PROMs (Breast-Q) completed**	**Yes/No**

For peer review only - http://bmjopen.bmj.com/site/about/guidelines.xhtml

The Pre-BRA (Pre-pectoral Breast Reconstruction Evaluation) Feasibility Study: Protocol
KL Harvey, N Mills, P White, C Holcombe, S Potter
Appendix 2.

Pre-BRA Section 4 - Operative data

Actual* date of primary mastectomy and reconstruction

........./………../……...(DD/MM/YYYY)

**If the planned (see section 1.) and actual dates of surgery differ, please contact central study team to confirm which date is correct. This is important for generating electronic BREAST-Q PROMS and post-operative pain score requests.*

ASA grade

1	Normal healthy individual	☐
2	Mild systemic disease that does not limit activities	☐
3	Severe systemic disease that limits activities but is not incapacitating	☐
4	Incapacitating systematic disease which is constantly life-threatening	☐

Name of operating Consultant surgeon

..

Procedure performed in theatre with laminar flow system in situ?

Yes ☐ No ☐

Type of skin prep used at time of surgery

Iodine (Betadine) (brown)	☐	Chlorhexidine (pink)	☐
2% Chlorprep	☐	Other (please specify)	☐

..

Planned antibiotic use

Prophylactic only (<24 hours)	☐	1-5 days	☐
Extended course (5+ days)	☐	Until drains removed	☐
Other (please specify)	☐		

……………………………………………………………………………………………………..

Antibiotics used, please include dose/ frequency (free text)

……………………………………………………………………………………………………..

Duration of procedure (knife to skin to dressings on) ....(Minutes)

For peer review only - http://bmjopen.bmj.com/site/about/guidelines.xhtml

The Pre-BRA (Pre-pectoral Breast Reconstruction Evaluation) Feasibility Study: Protocol
KL Harvey, N Mills, P White, C Holcombe, S Potter
Appendix 2.

Did you adhere to the steps advised in The Theatre Implant Checklist or 'TIC List'? (REDCap™ displays "TIC" List) Please tick all steps which were completed in this case:

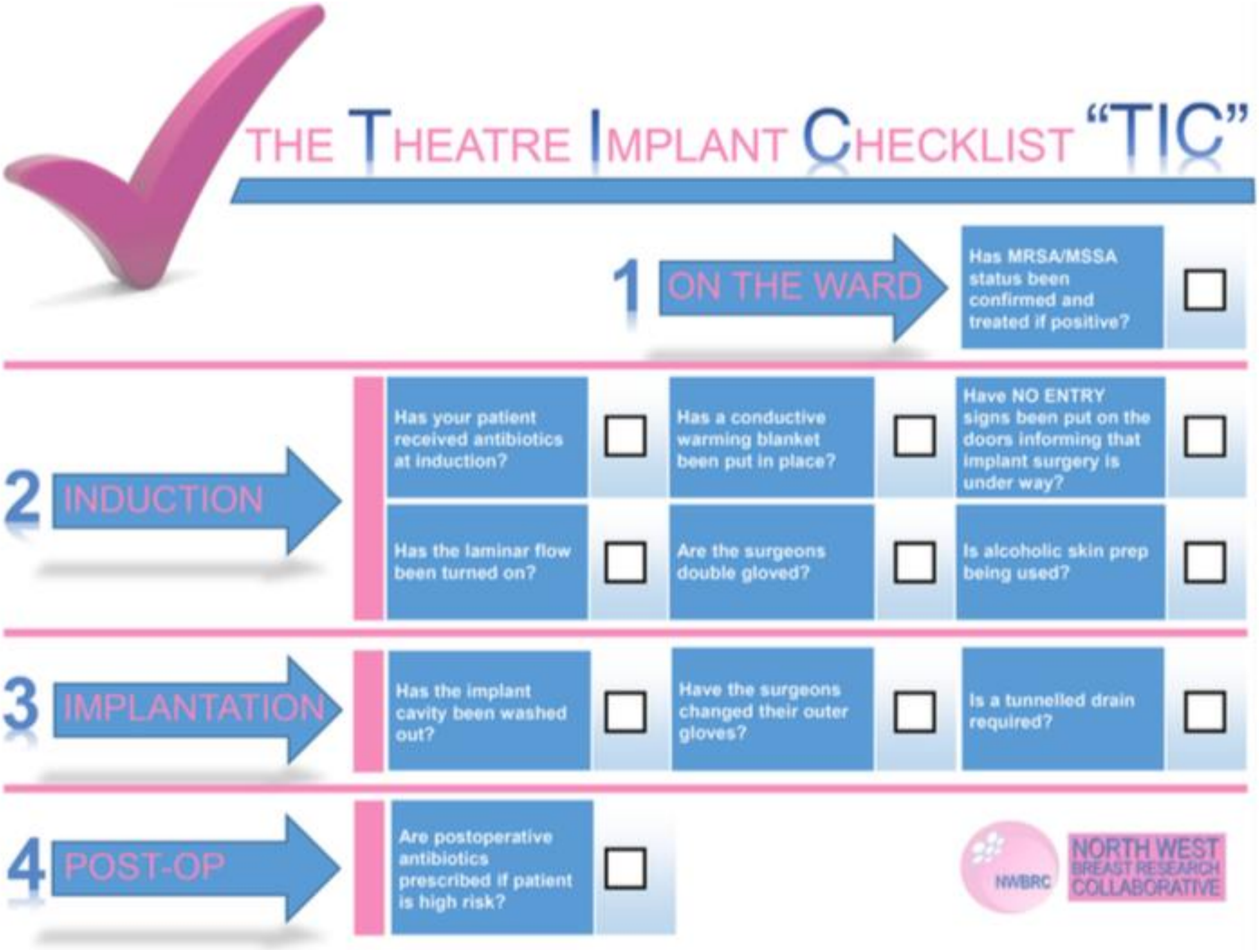

(Image reproduced from Barr, S.P., et al., *Infection prevention in breast implant surgery - A review of the surgical evidence, guidelines and a checklist.* European Journal of Surgical Oncology, 2016. **42**: p. 591-603.)

	Yes /	No
Patient received antibiotics at induction?	☐	☐
Conductive warming blanket been put in place?	☐	☐
NO ENTRY signs been put on the doors informing that implant surgery is under way?	☐	☐
Are the surgeons double gloved?	☐	☐
Is alcoholic skin prep being used?	☐	☐
Implant cavity washed out?	☐	☐
(Before implantation) Have the surgeons changed their outer gloves?	☐	☐
Is a tunnelled drain required?	☐	☐
Are post-operative antibiotics prescribed if patient is high risk?	☐	☐

For peer review only - http://bmjopen.bmj.com/site/about/guidelines.xhtml

The Pre-BRA (Pre-pectoral Breast Reconstruction Evaluation) Feasibility Study: Protocol
KL Harvey, N Mills, P White, C Holcombe, S Potter
Appendix 2.

Regarding length of stay, is the patient planned for:

Day case (i.e. home the same day as surgery) ☐

Overnight stay (i.e. home the day after surgery) ☐

Inpatient stay (>1 night) ☐

Was surgery performed on: (please tick all that apply)

Right breast ☐ Left breast ☐

Right breast procedure:

Implant reconstruction ☐
Other reconstruction ☐
Augmentation ☐
Reduction ☐
Mastopexy ☐
Other ☐

Left breast procedure:

Implant reconstruction ☐
Other reconstruction ☐
Augmentation ☐
Reduction ☐
Mastopexy ☐
Other ☐

If other selected, please give details: ……………………………………………………………………..

………

For peer review only

The Pre-BRA (Pre-pectoral Breast Reconstruction Evaluation) Feasibility Study: Protocol
KL Harvey, N Mills, P White, C Holcombe, S Potter
Appendix 2.

Pre-BRA Section 5a. RIGHT BREAST DATA

(Please only answer Section 5a. if you have performed a PRE-PECTORAL IMPLANT RECONSTRUCTION on the patient's RIGHT BREAST)

Pre-operative Planning: RIGHT BREAST

Right breast – What do you plan to do upfront i.e. prior to starting the operation?

One stage reconstruction ☐ Two-stage reconstruction ☐

Intra-operative Detail

Right breast - Grade of primary operating surgeon

Consultant ☐ SAS grade ☐
Senior trainee (OPF/ST8+) ☐ ST6/7 ☐
ST5 or below ☐ Other ☐

If other selected, please give details: ……………………………………………………………..

Right breast - Number of pre-pectoral implant-based reconstructions using this method/ technique the primary surgeon has **previously performed:**

In total (i.e. both supervised and unsupervised):

Less than 5 ☐ 11-25 ☐
5-10 ☐ More than 25 ☐

Supervised:

Less than 5 ☐ 11-25 ☐
5-10 ☐ More than 25 ☐

Right breast - Type of mastectomy

Skin sparing mastectomy ☐ Skin and nipple preserving ☐
Reduction (Wise) pattern ☐ Other ☐

If other selected, please give details: ……………………………………………………………..

………………………………………………………………………………………………………

For peer review only - http://bmjopen.bmj.com/site/about/guidelines.xhtml

The Pre-BRA (Pre-pectoral Breast Reconstruction Evaluation) Feasibility Study: Protocol
KL Harvey, N Mills, P White, C Holcombe, S Potter
Appendix 2.

Right breast - Location of incision

Peri-areolar (nipple sparing)	☐	Lateral	☐
Inframammary	☐	Elliptical removing NAC	☐
Wise pattern	☐	Other	☐

If other selected, please give details: ……………………………………………………………..

……………………………………………………………………………………………………

Right breast – Was infiltration in the mastectomy plane i.e. hydro-dissection used?

No	☐	With saline only	☐
With local anaesthetic +/- saline	☐	LA and adrenaline +/- saline	☐

Right breast - Main instrument used for dissection of mastectomy skin flaps

Blade	☐	Scissors	☐
Handheld monopolar diathermy	☐	Diathermy scissors	☐
Combination of methods	☐	Other	☐

If other selected, please give details: ……………………………………………………………..

……………………………………………………………………………………………………

Right breast - Mastectomy dry weight (gms) ………………………………………..

<u>Feasibility of Pre-Pectoral Reconstruction</u>

Right breast - Surgeon's assessment of skin flap quality

Good – healthy, well-perfused, no concerns noted at time of surgery ☐

Average – intermediate – no obvious concerns at time of surgery ☐

Poor – thin flaps, questionable vascularity; concerns at time of surgery ☐

Right breast - Did you complete the reconstruction as planned? (i.e. as per your answer to the first question of Section 5?)

Yes ☐ No ☐

For peer review only - http://bmjopen.bmj.com/site/about/guidelines.xhtml

The Pre-BRA (Pre-pectoral Breast Reconstruction Evaluation) Feasibility Study: Protocol
KL Harvey, N Mills, P White, C Holcombe, S Potter
Appendix 2.

Right breast - What operation did you actually perform?

i. Pre-pectoral approach considered safe and performed with fixed volume implant ☐

ii. Pre-pectoral reconstruction performed with variable volume implant (e.g. Becker) ☐

iii. Pre-pectoral reconstruction performed with a tissue expander ☐

iv. Pre-pectoral reconstruction abandoned ☐

If you selected answer iv. please also answer the next two questions (“Why?” and “What did you do instead?”)

If you answered i, ii or iii please go straight to the third question down (“Right breast - type of implant coverage”)

Why was pre-pectoral approach abandoned?

…………………………………………………………………………………………

…………………………………………………………………………………………..

…………………………………………………………………………………………

What procedure was performed instead

No reconstruction ☐

Sub-pectoral reconstruction with tissue expander (no mesh) ☐

Sub-pectoral reconstruction with biological mesh ☐

Sub-pectoral reconstruction with synthetic mesh ☐

Other ☐

If other selected, please give details: **………………………………………………………………..**

……

Right breast - type of implant coverage

No mesh or ADM coverage (subcutaneous implant only) ☐

Biological mesh (e.g. ADM) ☐

Synthetic mesh (e.g. TiLOOP) ☐

Complete dermal sling ☐

Dermal sling and biological mesh in combination ☐

For peer review only - http://bmjopen.bmj.com/site/about/guidelines.xhtml

The Pre-BRA (Pre-pectoral Breast Reconstruction Evaluation) Feasibility Study: Protocol
KL Harvey, N Mills, P White, C Holcombe, S Potter
Appendix 2.

Dermal sling and synthetic mesh in combination ☐

Hybrid implant coverage (e.g. biological mesh at top/synthetic mesh at bottom) ☐

Other ☐

If 'Hybrid implant coverage' or 'other' selected, please supply details: ……………………………

....

Right breast - type of implant coverage, if you selected 'Biological mesh' please select which type:

BRAXON	☐	Artia	☐
Surgimend	☐	Fortiva	☐
Strattice	☐	Cellis	☐
Veritas	☐	Exaflex Pocket	☐
MESO	☐	Native	☐
Tutomesh	☐		

Other biological ADM/ mesh (please state) ....☐

....

Right breast - type of implant coverage, if you selected 'synthetic mesh' please select which type:

TiLOOP	☐	Galaflex	☐
TiGR	☐		

Other (please state) ....☐

Right breast - Breast prosthesis details

Fixed volume implant ☐

Size (ccs)..

OR

Temporary tissue expander ☐

Vol of saline inserted (mls)…............................

Size when fully expanded (ccs)..........................

OR

Combined implant (Beckers) ☐

For peer review only - http://bmjopen.bmj.com/site/about/guidelines.xhtml

The Pre-BRA (Pre-pectoral Breast Reconstruction Evaluation) Feasibility Study: Protocol
KL Harvey, N Mills, P White, C Holcombe, S Potter
Appendix 2.

Silicone component (g).......................... Vol of saline inserted (mls)…........................

Size when fully expanded (ccs)........................

Right breast – shape of implant

Round ☐ Anatomical ☐

Right breast – texture of implant

Smooth ☐ Textured/ Microtextured ☐

Polyurethane ☐

Right breast – Was the prosthesis bathed/washed prior to insertion?

Yes ☐ No ☐

If 'Yes' selected which wash was used?

Saline ☐ Antiseptic ☐

Antibiotics ☐ Other (please give details) ☐

....

Right breast – Was the cavity washed or irrigated prior to insert of implant?

Yes ☐ No ☐

If 'Yes' selected what irrigation fluid was used?

Saline ☐ Antiseptic ☐

Antibiotics ☐ Other (please give details) ☐

....

Surgeon glove change prior to handling the implant?

Yes ☐ No ☐

Right Breast - Were the skin edges freshened/excised prior to wound closure?

Yes ☐ No ☐

Right breast – Type of axillary surgery

None ☐

For peer review only - http://bmjopen.bmj.com/site/about/guidelines.xhtml

The Pre-BRA (Pre-pectoral Breast Reconstruction Evaluation) Feasibility Study: Protocol
KL Harvey, N Mills, P White, C Holcombe, S Potter
Appendix 2.

SLNB/targeted sample post NAC ☐

Axillary sample (failed SLNB) ☐

Axillary clearance ☐

SLNB + ANC (e.g. positive OSNA) ☐

Right breast – Number of (tunnelled) drains used (per side if bilateral case)

None ☐ One ☐ Two ☐

Right breast – intra-operative analgesia used

Local/regional block (e.g. pectoral or serratus block) ☐

Infiltration of LA as part of hydro dissection/tumescence ☐

Infiltration of LA into wounds prior to starting case ☐

Infiltration of LA into wounds at the end of the case ☐

LA inserted down drains ☐

Intra-pocket LA catheter (continuous infusion of LA post-op) ☐

LA not used ☐

Other ☐

Right breast dressings used (please select as many as apply)

Occlusive adhesive dressing (e.g. Opsite) ☐

Skin glue (e.g. Liquiband) ☐

Skin closure system - skin glue and tape (e.g. Dermabond Prineo dressing) ☐

Negative pressure dressing e.g. PICO ☐

Other ☐

For peer review only

For peer review only - http://bmjopen.bmj.com/site/about/guidelines.xhtml

The Pre-BRA (Pre-pectoral Breast Reconstruction Evaluation) Feasibility Study: Protocol
KL Harvey, N Mills, P White, C Holcombe, S Potter
Appendix 2.

Pre-BRA Section 5b. LEFT BREAST DATA

(Please only answer Section 5b. if you have performed a PRE-PECTORAL IMPLANT RECONSTRUCTION on the patient's LEFT BREAST)

Pre-operative Planning: LEFT BREAST

Left breast – What do you plan to do upfront i.e. prior to starting the operation?

One stage reconstruction ☐ Two-stage reconstruction ☐

Intra-operative Detail

Left breast - Grade of primary operating surgeon

Consultant ☐ SAS grade ☐

Senior trainee (OPF/ST8+) ☐ ST6/7 ☐

ST5 or below ☐ Other ☐

If other selected, please give details: ………………………………………………………………..

Left breast - Number of pre-pectoral implant-based reconstructions using this method the primary surgeon has **previously performed:**

In total (i.e. both supervised and unsupervised):

Less than 5 ☐ 11-25 ☐

5-10 ☐ >25 ☐

Supervised:

Less than 5 ☐ 11-25 ☐

5-10 ☐ >25 ☐

Left breast - Type of mastectomy

Skin sparing mastectomy ☐ Skin and nipple preserving ☐

Reduction (wise) pattern ☐ Other ☐

If other selected, please give details: ………………………………………………………………..

………

For peer review only

The Pre-BRA (Pre-pectoral Breast Reconstruction Evaluation) Feasibility Study: Protocol
KL Harvey, N Mills, P White, C Holcombe, S Potter
Appendix 2.

Left breast - Location of incision

Peri-areolar (nipple sparing)	☐	Lateral	☐
Inframammary	☐	Elliptical removing NAC	☐
Wise pattern	☐	Other	☐

If other selected, please give details: ……………………………………………………………..

……

Left breast – Was infiltration in the mastectomy plane/hydro-dissection used?

No	☐	With saline only	☐
With local anaesthetic +/- saline	☐	LA and adrenaline +/- saline	☐

Left breast - Main instrument used for dissection of mastectomy skin flaps

Blade	☐	Scissors	☐
Handheld monopolar diathermy	☐	Diathermy scissors	☐
Combination of methods	☐	Other	☐

If other selected, please give details: ……………………………………………………………..

……

Left breast - Mastectomy dry weight (gms) ………………………………………

<u>Feasibility of Pre-Pectoral Reconstruction</u>

Left breast - Surgeon's assessment of skin flap quality

Good – healthy, well-perfused, no concerns noted at time of surgery ☐

Average – intermediate – no obvious concerns at time of surgery ☐

Poor – thin flaps, questionable vascularity; concerns at time of surgery ☐

Left breast - Did you complete the reconstruction as planned? (i.e. as per your answer to the first question of Section 3b?)

Yes ☐ No ☐

Left breast – if 'no', what operation did you actually perform?

For peer review only - http://bmjopen.bmj.com/site/about/guidelines.xhtml

The Pre-BRA (Pre-pectoral Breast Reconstruction Evaluation) Feasibility Study: Protocol
KL Harvey, N Mills, P White, C Holcombe, S Potter
Appendix 2.

i. Pre-pectoral approach considered safe and performed with fixed volume implant ☐

ii. Pre-pectoral reconstruction performed with variable volume implant (e.g. Becker) ☐

iii. Pre-pectoral reconstruction performed with a tissue expander ☐

iv. Pre-pectoral reconstruction abandoned ☐

If you selected answer iv. please also answer the next two questions ("Why?" and "What did you do instead")

If you answered i, ii or iii please go straight to the third question down ("Left breast - type of implant coverage")

Why was pre-pectoral approach abandoned?

…………………………………………………………………………………………

…………………………………………………………………………………………..

…………………………………………………………………………………………

What procedure was performed instead

No reconstruction ☐

Sub-pectoral reconstruction with tissue expander (no mesh) ☐

Sub-pectoral reconstruction with biological mesh ☐

Sub-pectoral reconstruction with synthetic mesh ☐

Other ☐

Left breast - type of implant coverage

No mesh or ADM coverage (subcutaneous implant only) ☐

Biological mesh (e.g. ADM) ☐

Synthetic mesh (e.g. TiLOOP) ☐

Complete dermal sling ☐

Dermal sling and biological mesh in combination ☐

Dermal sling and synthetic mesh in combination ☐

For peer review only - http://bmjopen.bmj.com/site/about/guidelines.xhtml

The Pre-BRA (Pre-pectoral Breast Reconstruction Evaluation) Feasibility Study: Protocol
KL Harvey, N Mills, P White, C Holcombe, S Potter
Appendix 2.

Hybrid implant coverage (e.g. biological mesh at top/synthetic mesh at bottom) ☐

Other ☐

If 'Hybrid implant coverage' or 'other' selected, please supply details: ……………………………

....

Left breast - type of implant coverage, if you selected 'Biological mesh' please select which type:

BRAXON ☐ Artia ☐

Surgimend ☐ Fortiva ☐

Strattice ☐ Cellis ☐

Veritas ☐ Exaflex Pocket ☐

MESO ☐ Native ☐

Tutomesh ☐

Other biological ADM/ mesh (please state) ....☐

....

Left breast - type of implant coverage, if you selected 'synthetic mesh' please select which type:

TiLOOP ☐ Galaflex ☐

TiGR ☐

Other (please state) ....☐

Left breast - Breast prosthesis details

Fixed volume implant ☐

Size (ccs)..

OR

Temporary tissue expander ☐

Vol of saline inserted (mls)…...........................

Size when fully expanded (ccs)..........................

OR

Combined implant (Beckers) ☐

Silicone component (g).......................... Vol of saline inserted (mls)…........................

For peer review only - http://bmjopen.bmj.com/site/about/guidelines.xhtml

For peer review only

The Pre-BRA (Pre-pectoral Breast Reconstruction Evaluation) Feasibility Study: Protocol
KL Harvey, N Mills, P White, C Holcombe, S Potter
Appendix 2.

Size when fully expanded (ccs)........................

Left breast – shape of implant

Round ☐ Anatomical ☐

Left breast – texture of implant

Smooth ☐ Textured/ Microtextured ☐

Polyurethane ☐ I don't know ☐

Left breast – Was the prosthesis bathed/washed prior to insertion?

Yes ☐ No ☐

If 'Yes' selected which wash was used?

Saline ☐ Antiseptic ☐

Antibiotics ☐ Other (please give details) ☐

....

Left breast – Was the cavity washed or irrigated prior to insert of implant?

Yes ☐ No ☐

If 'Yes' selected what irrigation fluid was used?

Saline ☐ Antiseptic ☐

Antibiotics ☐ Other (please give details) ☐

....

Left Breast - Surgeon glove change prior to handling the implant?

Yes ☐ No ☐

Left Breast - Were the skin edges freshened/excised prior to wound closure?

Yes ☐ No ☐

Left breast – Type of axillary surgery

None ☐ (Please go to question 3.36)

SLNB/targeted sample post NAC ☐ Axillary sample (failed SLNB) ☐

Axillary clearance ☐ SLNB + ANC (e.g. positive OSNA) ☐

For peer review only - http://bmjopen.bmj.com/site/about/guidelines.xhtml

The Pre-BRA (Pre-pectoral Breast Reconstruction Evaluation) Feasibility Study: Protocol
KL Harvey, N Mills, P White, C Holcombe, S Potter
Appendix 2.

Left breast – Number of (tunnelled) drains used

None ☐ One ☐ Two ☐

Left breast – intra-operative analgesia used

Local/regional block (e.g. pectoral or serratus block) ☐

Infiltration of LA as part of hydro dissection/tumescence ☐

Infiltration of LA into wounds prior to starting case ☐

Infiltration of LA into wounds at the end of the case ☐

LA inserted down drains ☐

LA not used ☐

Intra-pocket LA catheter (continuous infusion of LA post-op)

Other ☐

Left breast dressings used (select as many as apply)

Occlusive adhesive dressing (e.g. Opsite) ☐

Skin glue (e.g. Liquiband) ☐

Skin closure system - skin glue and tape (e.g. Dermabond Prineo dressing) ☐

Negative pressure dressing e.g. PICO ☐

Other

For peer review only - http://bmjopen.bmj.com/site/about/guidelines.xhtml

The Pre-BRA (Pre-pectoral Breast Reconstruction Evaluation) Feasibility Study: Protocol
KL Harvey, N Mills, P White, C Holcombe, S Potter
Appendix 2.

Pre-BRA Section 6 – Modifications to Usual Operative Technique

Were any modifications made to the operative technique used in this case?

Yes ☐ No ☐

If you have answered "**yes**", please provide details about:

i. What was modified and how? (e.g. mesh sutured in a different way) ………………………...

…………………………………………………………………………………………………….

…………………………………………………………………………………………………….

…………………………………………………………………………………………………….

ii. Why was the modification made? (e.g. difficulty accessing mesh) …………………………...

…………………………………………………………………………………………………….

…………………………………………………………………………………………………….

…………………………………………………………………………………………………….

<u>**Please Note**</u>: The study team are very interested in speaking to surgeons to explore any modifications made to the procedure.

Does the operating surgeon consent to be contacted for a brief telephone interview?

Yes ☐ No ☐

Please supply the email address of the operating surgeon if yes:

………………………………………………………………………………………………

Pre-BRA Section 7 – Peri Discharge

<u>**Length of Stay**</u>

Was the patient managed as a day case?
(defined as: discharged on the same day as surgery)

Yes ☐ No ☐

For peer review only - http://bmjopen.bmj.com/site/about/guidelines.xhtml

The Pre-BRA (Pre-pectoral Breast Reconstruction Evaluation) Feasibility Study: Protocol
KL Harvey, N Mills, P White, C Holcombe, S Potter
Appendix 2.

Date of discharge (DD/MM/YYYY) ………………/……………/……………………..

Length of post-operative stay…………..……………… (no. of post-operative days in hospital, calculated from date of surgery (= Day 0) to date of discharge)

(REDCap will auto-populate using the answers from date of surgery and the answer above)

For peer review only

END OF PRE-OPERATIVE AND OPERATIVE DATA SECTION

For peer review only - http://bmjopen.bmj.com/site/about/guidelines.xhtml

The Pre-BRA (Pre-pectoral Breast Reconstruction Evaluation) Feasibility Study: Protocol
KL Harvey, N Mills, P White, C Holcombe, S Potter
Appendix 2.

Pain Scores

Pre-BRA

Please NOTE:

If the participant does not wish to complete pains core data entry online via email link they must be collected in person/ via telephone call and transcribed into REDCap's "Pain scores" section by the research team

Pain score at 24 hours' post-reconstruction **(0 - 10)*** ………………

Pain score at 1-week post-reconstruction: **(0 - 10)** ………………

Pain score at 2 weeks' post-reconstruction: **(0 - 10)** ………………

Pain score at 3 months' post-reconstruction: **(0 - 10)** ………………

* 0-10 Numeric pain rating scale, where:

0= No pain, 5= Moderate pain, 10= worst possible pain

For peer review only - http://bmjopen.bmj.com/site/about/guidelines.xhtml

The Pre-BRA (Pre-pectoral Breast Reconstruction Evaluation) Feasibility Study: Protocol
KL Harvey, N Mills, P White, C Holcombe, S Potter
Appendix 2.

3 Month Data

Pre-BRA

For peer review only

For peer review only - http://bmjopen.bmj.com/site/about/guidelines.xhtml

The Pre-BRA (Pre-pectoral Breast Reconstruction Evaluation) Feasibility Study: Protocol
KL Harvey, N Mills, P White, C Holcombe, S Potter
Appendix 2.

Pre-BRA Section 8 – Oncological outcome and adjuvant treatment data

Not applicable – risk reducing surgery ☐ (No further questions in Section 8)

RIGHT BREAST

(Only answer the following questions if you operated for malignancy on the patient's **right breast)**

Right breast – if neoadjuvant chemotherapy – complete pathological response?

Yes ☐ No ☐

Right breast - Invasive status

Invasive ☐ DCIS ☐

Right breast – Focality

Unifocal ☐ Multifocal ☐

Right breast - Grade of DCIS or invasive carcinoma

(from core biopsy if complete pathological response)

1 – Low grade (DCIS) or well-differentiated (invasive) ☐

2 – Intermediate grade (DCIS) or moderately differentiated (invasive) ☐

3 – High grade (DCIS) or poorly differentiated (invasive) ☐

Right breast - Lymph node involvement

Total number of involved nodes (macromets only) ....

Total number of lymph nodes in pathology specimen...

Right breast - Invasive lesion size (mm)

(largest lesion if multifocal) ....

Right side - Planned delayed axillary clearance

Yes ☐ No ☐

LEFT BREAST

(Only answer the following questions if you operated for malignancy on the patient's **left breast)**

Left breast – if neoadjuvant chemotherapy – complete pathological response?

Yes ☐ No ☐

Left breast - Invasive status

For peer review only - http://bmjopen.bmj.com/site/about/guidelines.xhtml

For peer review only

The Pre-BRA (Pre-pectoral Breast Reconstruction Evaluation) Feasibility Study: Protocol
KL Harvey, N Mills, P White, C Holcombe, S Potter
Appendix 2.

Invasive ☐ DCIS ☐

Left breast – Focality

Unifocal ☐ Multifocal ☐

Left breast - Grade of DCIS or invasive carcinoma
(from core biopsy if complete pathological response)

1 – Low grade (DCIS) or well-differentiated (invasive) ☐

2 – Intermediate grade (DCIS) or moderately differentiated (invasive) ☐

3 – High grade (DCIS) or poorly differentiated (invasive) ☐

Left breast - Lymph node involvement

6.13.1 Total number of involved nodes (macromets only) ....

6.13.2 Total number of lymph nodes in pathology specimen.......................................

Left breast - Invasive lesion size (mm)

(largest lesion if multifocal) ....

Left side - Planned delayed axillary clearance

Yes ☐ No ☐

<u>PLANNED ADJUVANT THERAPY</u>
(Answer the following questions for **all cases of malignancy**)

Chemotherapy

Yes ☐ No ☐ Already received ☐

Radiotherapy

Right side

to chest wall Yes ☐ No ☐

to axilla Yes ☐ No ☐

to SCF* Yes ☐ No ☐

to IMC** Yes ☐ No ☐

Left side

to chest wall Yes ☐ No ☐

to axilla Yes ☐ No ☐

to SCF* Yes ☐ No ☐

For peer review only - http://bmjopen.bmj.com/site/about/guidelines.xhtml

The Pre-BRA (Pre-pectoral Breast Reconstruction Evaluation) Feasibility Study: Protocol
KL Harvey, N Mills, P White, C Holcombe, S Potter
Appendix 2.

to IMC** Yes ☐ No ☐

(*Supraclavicular fossa, ** internal mammary chain)

Endocrine therapy Yes ☐ No ☐

Anti-Her2 therapy Yes ☐ No ☐

Date of FIRST adjuvant treatment
(i.e. date of first dose of chemotherapy, if applicable or 1st fraction of radiotherapy)

(DD/MM/YYYY)……………………………………………

Pre-BRA Section 9– Complication(s) at 3 Months

Post-operative complication experienced IN THE FIRST 3 MONTHS?

Yes ☐ No ☐

Readmission to hospital IN THE FIRST 3 MONTHS
Was the patient readmitted to hospital to manage a complication in the first 3 months post-operatively?

Yes ☐ No ☐ Date (DD/MM/YYYY) ……………………

If 'Yes' selected, please give reason for readmission:

………………………………………………………………………………………

………………………………………………………………………………………..

………………………………………………………………………………………

Re-operation for complications IN THE FIRST 3 MONTHS
Did the patient undergo an unplanned re-operation (i.e. following a complication) in the first 3 months post-operatively?

Yes ☐ No ☐ Date (DD/MM/YYYY) ……………………

If 'Yes' selected, please give reason for re-operation:

………………………………………………………………………………………

………………………………………………………………………………………..

………………………………………………………………………………………

For peer review only - http://bmjopen.bmj.com/site/about/guidelines.xhtml
For peer review only

The Pre-BRA (Pre-pectoral Breast Reconstruction Evaluation) Feasibility Study: Protocol
KL Harvey, N Mills, P White, C Holcombe, S Potter
Appendix 2.

Breast Complication Details:

Please indicate if a complication was experienced, indicate which side was affected and the date it was first noticed

	Right	Left	Bilateral	Date (DD/MM/YYYY)
**Seroma?**				
No aspiration required	☐	☐	☐	....
Requiring aspiration 1-2 times	☐	☐	☐	....
Requiring aspiration 3 or more times	☐	☐	☐	....
**Haematoma?**				
Minor - Managed conservatively	☐	☐	☐	....
Major 1 - Requiring aspiration in clinic (+/-USS, no GA)	☐	☐	☐	....
Major 2 - Requiring evacuation <u>in theatre</u>	☐	☐	☐	....
**Wound infection?**				
Minor - Requiring oral antibiotics	☐	☐	☐	....
Major 1 - Requiring admission for IV antibiotics	☐	☐	☐	....
Major 2 - Requiring surgical drainage or debridement	☐	☐	☐	....
**Mastectomy skin flap necrosis?**				
Minor – Managed conservatively	☐	☐	☐	....
Major 1 – Requiring surgical debridement in clinic (no GA)	☐	☐	☐	....
Major 2 – Requiring surgical debridement <u>in theatre</u> (GA)	☐	☐	☐	....
**Nipple necrosis?**				
Minor – Managed conservatively	☐	☐	☐	....
Major 1 – Requiring surgical debridement	☐	☐	☐	....
Major 2 – Total NAC loss	☐	☐	☐	....

For peer review only

The Pre-BRA (Pre-pectoral Breast Reconstruction Evaluation) Feasibility Study: Protocol
KL Harvey, N Mills, P White, C Holcombe, S Potter
Appendix 2.

	Right	Left	Bilateral	Date (DD/MM/YYYY)
**Wound dehiscence?**				
Minor – Managed conservatively	☐	☐	☐	…………………………
Major – Requiring return to theatre for re-suturing	☐	☐	☐	…………………………
**Implant loss?**				
Total implant loss i.e. any unplanned removal of the implant or expander for infection, **without replacement** of the prosthesis	☐	☐	☐	…………………………
**OR** ***Implant salvage*** i.e. i. Implant removed, washed and replaced with fixed volume/ final implant	☐	☐	☐	…………………………
**OR** ii. Implant removed, washed and replaced with a tissue expander	☐	☐	☐	…………………………

(If "***Implant salvage***" selected, additional question:
Was implant salvage a success? (i.e. implant still in place at 3 months)

Yes ☐ No ☐

	Right	Left	Bilateral	Date (DD/MM/YYYY)
**Mechanical implant problem? (e.g. deflation)**	☐	☐	☐	…………………………
**Other complication?**	☐	☐	☐	…………………………

Details………………………………………………………………………………………………

Pre-BRA Section 10 – Learning from Post-Operative Problems

Would the operating surgeon consider changing or modifying their **surgical technique** as a result of this complication? Yes ☐ No ☐

Please supply as much detail as possible: ………………………………………………………………

………

………

For peer review only - http://bmjopen.bmj.com/site/about/guidelines.xhtml

The Pre-BRA (Pre-pectoral Breast Reconstruction Evaluation) Feasibility Study: Protocol
KL Harvey, N Mills, P White, C Holcombe, S Potter
Appendix 2.
……………………………………………………………………………………………………

Would the operating surgeon consider changing or modifying their **patient selection criteria** as a result of this complication? Yes ☐ No ☐

Please supply as much detail as possible: …………………………………………………………
……………………………………………………………………………………………………
……………………………………………………………………………………………………
……………………………………………………………………………………………………

Please note: The study team are **very interested** in speaking to surgeons who report learning from post-operative problems.
Does the operating surgeon agree to be contacted for a brief telephone interview?

Yes ☐ No ☐

Please supply the email address of the operating surgeon if yes:

……………………………………………………………………………………

Pre-BRA Section 11 – Post-Operative Patient Reported Outcome Measures (PROMS)

7.1 **3-month Breast-Q completed?** Yes ☐ No ☐

7.2 **18-month Breast-Q completed?** Yes ☐ No ☐

END OF QUESTIONS

THANK YOU FROM THE PRE-BRA TEAM!

Pre-BRA

For peer review only - http://bmjopen.bmj.com/site/about/guidelines.xhtml

The Pre-BRA (Pre-pectoral Breast Reconstruction Evaluation) Feasibility Study: Protocol
KL Harvey, N Mills, P White, C Holcombe, S Potter
Appendix 3

Appendix 3

Definitions of Complications

Implant loss - any unplanned removal of the implant without replacement of the prosthesis (implant or expander) for infection, wound problems or other indication within the first 3 months following surgery.

Implant salvage – return to theatre for debridement of wound/ re-suturing /drainage of infection/ washout/ or other indication in which the implant is removed and immediately replaced either with the same device or a tissue expander with primary closure of the wound.

Unsuccessful implant salvage (considered equivalent to implant loss) - the implant is no longer in situ 3 months after the salvage procedure was performed.

Infection - A hot, red swollen breast associated with one of the following; a temperature, pus at the wound site, a raised white cell count and/or; a positive wound swab within the first 3 months following surgery. This will be further classified as:

- Minor – requiring oral antibiotics only;
- Major 1 – requiring admission for IV antibiotics and/or debridement;
- Major 2 – requiring surgical drainage/debridement

Red breast syndrome – Persistent non-infective breast erythema

Readmission to hospital – any re-admission to hospital in the 3 months following surgery directly related to the procedure (e.g. with infection requiring antibiotics).

For peer review only

The Pre-BRA (Pre-pectoral Breast Reconstruction Evaluation) Feasibility Study: Protocol
KL Harvey, N Mills, P White, C Holcombe, S Potter
Appendix 3

Return to theatre – Return to the operating theatre at any time during the first 3 months following surgery to deal with any complication of the reconstruction. This will not include any secondary oncological procedures such as axillary clearance or planned procedures including exchange of expander for a fixed volume implant or lipo-modelling.

Seroma - A symptomatic collection of fluid around the reconstructed breast following surgery requiring aspiration. The total number of aspirations will be collected at 3 months.

Haematoma - A collection of blood in the reconstructed breast

- Minor – managed conservatively or by aspiration in clinic;
- Major – requiring surgical evacuation

Mastectomy skin flap necrosis - Any area of skin loss on the reconstructed breast

- Minor – managed conservatively with dressings;
- Major 1 – requiring debridement (in clinic or theatre) under local anaesthetic (LA);
- Major 2 – requiring surgical debridement under general anaesthesia (GA)

Nipple necrosis – Any area of necrosis of the nipple areolar complex (NAC) (if nipple preserving mastectomy)

- Minor – managed conservatively with dressings;
- Major 1 – requiring surgical debridement under LA in clinic or theatre;
- Major 2 – requiring surgical debridement under GA in theatre

Wound dehiscence – separation of the skin edges at the wound site.

- Minor – treated conservatively;

For peer review only

The Pre-BRA (Pre-pectoral Breast Reconstruction Evaluation) Feasibility Study: Protocol
KL Harvey, N Mills, P White, C Holcombe, S Potter
Appendix 3

- Major – requiring return to theatre for re-suturing under GA

Displaced implant requiring repositioning under GA – any implant displacement that requires surgical correction to restore its position

In hospital complication – any complication that occurs during the patient's initial hospital stay at the time of their reconstructive surgery. This includes systematic complications such as DVT/PE and procedure specific complications such as haematoma.

Major complication - Any complication requiring readmission to hospital or return to theatre

Minor complication - Any other complication

Surgeons will be asked to report any learning arising from complications and whether they would be willing to be contacted to discuss this in more detail.

For peer review only

The Pre-BRA (Pre-pectoral Breast Reconstruction Evaluation) Feasibility Study: Protocol
KL Harvey, N Mills, P White, C Holcombe, S Potter
Appendix 4.

Appendix 4.

The Pre-Bra Study: Surgeon Survey

This survey is for surgeons performing pre-pectoral reconstruction who plan to recruit patients to the Pre-Bra study.

<u>Part 1: Your Details</u>

1. **Your name**……………………………………………………………………………………………

2. **Name of your hospital**……………………………………………………………………………

3. **City where your hospital is based** ………………………………………………………………

4. **What year did you become a consultant surgeon/ associate specialist?**........................

5. **Do you perform pre-pectoral breast reconstruction (PPBR)?** Yes ☐ No ☐

   (If you answered 'no" to question 5, no further answers are required. Thank you, you are not eligible to complete the questionnaire)

6. **If yes, how long have you been practising the technique of PPBR?**

   <6 months ☐ 2-5 years ☐

   6 -12 months ☐ 5-10 years ☐

   1-2 years ☐ > 10 years ☐

7. **a. IF <6 months or 6-12 months selected:** approximately how many PPBR have you performed to date?

   0-5 ☐ 11-20 ☐ 51-100 ☐

   6-10 ☐ 21-50 ☐ >100 ☐

   b. OR, IF >1 year selected: How many pre-pectoral reconstructions do you perform annually?

For peer review only

The Pre-BRA (Pre-pectoral Breast Reconstruction Evaluation) Feasibility Study: Protocol
KL Harvey, N Mills, P White, C Holcombe, S Potter
Appendix 4.

0-5	☐	11-20	☐	51-100	☐
6-10	☐	21-50	☐	>100	☐

8. Have you had training or mentorship in the technique of pre-pectoral reconstruction?

Yes ☐ No ☐

9. If you have answered yes to Q8. RedCAP™ offers the following options:

As a trainee? ☐ **OR** As a consultant? ☐

10. If you have answered yes to Q8. RedCAP™ offers the following options, please tick all that apply:

A National TIG Fellowship ☐

A training course ☐

An industry workshop/study day ☐

Visit to an established PPBR centre before performing your first case(s) ☐

Experienced PPBR surgeon-mentor in theatre during your first case(s) ☐

Another Oncoplastic fellowship (details below please) ☐

……

……

Other (please state) ☐ ……………………………………………………………………..............

……

……

For peer review only

The Pre-BRA (Pre-pectoral Breast Reconstruction Evaluation) Feasibility Study: Protocol
KL Harvey, N Mills, P White, C Holcombe, S Potter
Appendix 4.

Part 2: Your Patient Selection Criteria

11. Which factors affect your decision-making when offering a patient PPBR?

(please tick all that apply)

Breast size ☐ Patient age ☐

Patient BMI ☐ Degree of ptosis ☐

Patient hobbies or levels of activity ☐

Patient preference (e.g. to avoid implant animation) ☐

Likely need for post-mastectomy radiotherapy ☐

12. Which (if any) of the following would you consider a contraindication to pre-pectoral breast reconstruction? (please tick all that apply, RedCAP matrix)

	Not a contraindication	Relative contraindication	Absolute contraindication
Current smoker			
BMI >30			
Grade 3 or 4 ptosis			
Predicted implant volume >600 cc			
Breast size D cup or more			
Likely requirement for post-mastectomy radiotherapy			
Previous radiotherapy			
Thin skin flaps (e.g. pinch test <1cm)			

For peer review only

The Pre-BRA (Pre-pectoral Breast Reconstruction Evaluation) Feasibility Study: Protocol
KL Harvey, N Mills, P White, C Holcombe, S Potter
Appendix 4.

Diabetes			
Steroid use			
Connective tissue disease			

Part 3: Your Peri-operative Practice

13. What mesh(es) or ADMs do you use? (Please tick all that apply)

BRAXON ☐ TiLOOP ☐ MESO ☐

Strattice ☐ Fortiva ☐ TIGR ☐

Surgimend ☐ Artia ☐ Other ☐

(If other please give details –name and manufacturer)..

....

14. What is your mesh of choice/ preference? ....

....

15. Would you try a new mesh? Yes ☐ No ☐ Maybe ☐

16. If yes, what would make you consider trying something new?

Evaluation within a protocoled study ☐

Industry representative (rep) recommendation ☐

Evidence of Safety ☐

Any other reason ☐ ....

....

For peer review only

The Pre-BRA (Pre-pectoral Breast Reconstruction Evaluation) Feasibility Study: Protocol
KL Harvey, N Mills, P White, C Holcombe, S Potter
Appendix 4.

17. What implant(s) do you use? (Please tick all that apply)

Mentor ☐ Nagor ☐ B-Lite ☐

Allergan ☐ Eurosurgical ☐ Sebbin ☐

Other ☐ (please give details –name and manufacturer)....................................

....

18. Do you use tissue expanders in the PPBR population? Yes ☐ No ☐

19. If yes, how? (please select all that apply)

☐ I routinely perform two-stage PPBR with tissue expanders in all/ most cases

☐ I use two-stage surgery with tissue expanders in some patients I consider to be high risk (i.e. this is your planned pre-operative strategy)

☐ I convert to two-stage surgery with tissue expanders in some patients I consider to be high risk (i.e. this is an intra-operative decision based upon patient factors such as skin flaps)

☐ I use tissue expanders for PPBR in another way (please give details)

....

....

20. Do you have a protocol for the management of patients undergoing pre-pectoral reconstruction? (e.g. re: drains and antibiotics) Yes ☐ No ☐

21. How many post-operative drains to you routinely use?

None ☐ One ☐ Two ☐

other (please state) ☐……………………………………………………………………………..

For peer review only

The Pre-BRA (Pre-pectoral Breast Reconstruction Evaluation) Feasibility Study: Protocol
KL Harvey, N Mills, P White, C Holcombe, S Potter
Appendix 4.

………

22. **If one/ two/ other: How long do the drains stay in?** (free text)………………………………

………

23. **What antibiotics do you use (name and dose) and for how long**?.................................

………

24. **Do you routinely use any of the following strategies to reduce infection? (Please tick all that apply):**

Laminar flow theatre ☐ 'Clean' drapes ☐

Pocket wash ☐ 'Clean' instruments ☐

Tunnelling of drains ☐

Refreshing/trimming of skin edges prior to closure ☐

Glove change prior to implant insertion ☐

For peer review only

<u>Part 4: Your Post-operative Practice</u>

25. **How many routine post-operative attendances are your implant-reconstruction patients invited to attend?**

One ☐ Three ☐

Two ☐ >3 times ☐

Varies between cases ☐

Other……………………………………………………………………………………………

26. **When do you see patients in the early post-operative period?** (please select all that apply)

Before 1 week ☐ At 1 week ☐

The Pre-BRA (Pre-pectoral Breast Reconstruction Evaluation) Feasibility Study: Protocol
KL Harvey, N Mills, P White, C Holcombe, S Potter
Appendix 4.

At 2 weeks	☐	At 6 weeks	☐
At 3 weeks	☐	Beyond 6 weeks	☐
At 4 weeks	☐	It varies between cases	☐
At 5 weeks	☐	No routine follow-up	☐

Other ☐……………………………………………………………………………………

Part 5: Interviews with Surgeons

The study team are interested in identifying best practice for pre-pectoral breast reconstruction and would like to speak to surgeons performing the technique to understand how they do the operation, what works well and why.

27. Would you be prepared to be contacted by the study team to take part in a brief interview to discuss your approach to pre-pectoral breast reconstruction? All interviews will be conducted by telephone, should take no more than 20 minutes and information collected will be anonymised. Yes ☐ No ☐

28. If yes, please supply an Email address……………………………………………………………

END

Thank you for your answers

For peer review only

Reporting checklist for protocol of a clinical trial.

Based on the SPIRIT guidelines.

Instructions to authors

Complete this checklist by entering the page numbers from your manuscript where readers will find each of the items listed below.

Your article may not currently address all the items on the checklist. Please modify your text to include the missing information. If you are certain that an item does not apply, please write "n/a" and provide a short explanation.

Upload your completed checklist as an extra file when you submit to a journal.

In your methods section, say that you used the SPIRITreporting guidelines, and cite them as:

Chan A-W, Tetzlaff JM, Altman DG, Laupacis A, Gøtzsche PC, Krleža-Jerić K, Hróbjartsson A, Mann H, Dickersin K, Berlin J, Doré C, Parulekar W, Summerskill W, Groves T, Schulz K, Sox H, Rockhold FW, Rennie D, Moher D. SPIRIT 2013 Statement: Defining standard protocol items for clinical trials. Ann Intern Med. 2013;158(3):200-207

		Reporting Item	Page Number
**Administrative information**			
Title	#1	Descriptive title identifying the study design, population, interventions, and, if applicable, trial acronym	1
Trial registration	#2a	Trial identifier and registry name. If not yet registered, name of intended registry	3
Trial registration: data set	#2b	All items from the World Health Organization Trial Registration Data Set	3
Protocol version	#3	Date and version identifier	*Not applicable*
Funding	#4	Sources and types of financial, material, and other support	19,20

For peer review only

Roles and responsibilities: contributorship	#5a	Names, affiliations, and roles of protocol contributors	19
Roles and responsibilities: sponsor contact information	#5b	Name and contact information for the trial sponsor	19
Roles and responsibilities: sponsor and funder	#5c	Role of study sponsor and funders, if any, in study design; collection, management, analysis, and interpretation of data; writing of the report; and the decision to submit the report for publication, including whether they will have ultimate authority over any of these activities	*Not applicable*
Roles and responsibilities: committees	#5d	Composition, roles, and responsibilities of the coordinating centre, steering committee, endpoint adjudication committee, data management team, and other individuals or groups overseeing the trial, if applicable (see Item 21a for data monitoring committee)	19
**Introduction**			
Background and rationale	#6a	Description of research question and justification for undertaking the trial, including summary of relevant studies (published and unpublished) examining benefits and harms for each intervention	5
Background and rationale: choice of comparators	#6b	Explanation for choice of comparators	5
Objectives	#7	Specific objectives or hypotheses	6
Trial design	#8	Description of trial design including type of trial (eg, parallel group, crossover, factorial, single group), allocation ratio, and framework (eg, superiority, equivalence, non-inferiority, exploratory)	7
**Methods: Participants, interventions, and outcomes**			
Study setting	#9	Description of study settings (eg, community clinic, academic hospital) and list of countries where data will be	7

For peer review only

		collected. Reference to where list of study sites can be obtained	
Eligibility criteria	#10	Inclusion and exclusion criteria for participants. If applicable, eligibility criteria for study centres and individuals who will perform the interventions (eg, surgeons, psychotherapists)	8
Interventions: description	#11a	Interventions for each group with sufficient detail to allow replication, including how and when they will be administered	10
Interventions: modifications	#11b	Criteria for discontinuing or modifying allocated interventions for a given trial participant (eg, drug dose change in response to harms, participant request, or improving / worsening disease)	10
Interventions: adherance	#11c	Strategies to improve adherence to intervention protocols, and any procedures for monitoring adherence (eg, drug tablet return; laboratory tests)	10
Interventions: concomitant care	#11d	Relevant concomitant care and interventions that are permitted or prohibited during the trial	11
Outcomes	#12	Primary, secondary, and other outcomes, including the specific measurement variable (eg, systolic blood pressure), analysis metric (eg, change from baseline, final value, time to event), method of aggregation (eg, median, proportion), and time point for each outcome. Explanation of the clinical relevance of chosen efficacy and harm outcomes is strongly recommended	12
Participant timeline	#13	Time schedule of enrolment, interventions (including any run-ins and washouts), assessments, and visits for participants. A schematic diagram is highly recommended (see Figure)	13
Sample size	#14	Estimated number of participants needed to achieve study objectives and how it was determined, including clinical and statistical assumptions supporting any sample size calculations	16
Recruitment	#15	Strategies for achieving adequate participant enrolment to reach target sample size	16

For peer review only

**Methods: Assignment of interventions (for controlled trials)**			
Allocation: sequence generation	#16a	Method of generating the allocation sequence (eg, computer-generated random numbers), and list of any factors for stratification. To reduce predictability of a random sequence, details of any planned restriction (eg, blocking) should be provided in a separate document that is unavailable to those who enrol participants or assign interventions	*Not applicable*
Allocation concealment mechanism	#16b	Mechanism of implementing the allocation sequence (eg, central telephone; sequentially numbered, opaque, sealed envelopes), describing any steps to conceal the sequence until interventions are assigned	*Not applicable*
Allocation: implementation	#16c	Who will generate the allocation sequence, who will enrol participants, and who will assign participants to interventions	*Not applicable*
Blinding (masking)	#17a	Who will be blinded after assignment to interventions (eg, trial participants, care providers, outcome assessors, data analysts), and how	*Not applicable*
Blinding (masking): emergency unblinding	#17b	If blinded, circumstances under which unblinding is permissible, and procedure for revealing a participant's allocated intervention during the trial	*Not applicable*
**Methods: Data collection, management, and analysis**			
Data collection plan	#18a	Plans for assessment and collection of outcome, baseline, and other trial data, including any related processes to promote data quality (eg, duplicate measurements, training of assessors) and a description of study instruments (eg, questionnaires, laboratory tests) along with their reliability and validity, if known. Reference to where data collection forms can be found, if not in the protocol	13
Data collection plan: retention	#18b	Plans to promote participant retention and complete follow-up, including list of any outcome data to be collected for	13

For peer review only

		participants who discontinue or deviate from intervention protocols	See appendix 4
Data management	#19	Plans for data entry, coding, security, and storage, including any related processes to promote data quality (eg, double data entry; range checks for data values). Reference to where details of data management procedures can be found, if not in the protocol	13, 19
Statistics: outcomes	#20a	Statistical methods for analysing primary and secondary outcomes. Reference to where other details of the statistical analysis plan can be found, if not in the protocol	17
Statistics: additional analyses	#20b	Methods for any additional analyses (eg, subgroup and adjusted analyses)	17
Statistics: analysis population and missing data	#20c	Definition of analysis population relating to protocol non-adherence (eg, as randomised analysis), and any statistical methods to handle missing data (eg, multiple imputation)	17
**Methods: Monitoring**			
Data monitoring: formal committee	#21a	Composition of data monitoring committee (DMC); summary of its role and reporting structure; statement of whether it is independent from the sponsor and competing interests; and reference to where further details about its charter can be found, if not in the protocol. Alternatively, an explanation of why a DMC is not needed	*Not applicable*
Data monitoring: interim analysis	#21b	Description of any interim analyses and stopping guidelines, including who will have access to these interim results and make the final decision to terminate the trial	17
Harms	#22	Plans for collecting, assessing, reporting, and managing solicited and spontaneously reported adverse events and other unintended effects of trial interventions or trial conduct	13
Auditing	#23	Frequency and procedures for auditing trial conduct, if any, and whether the process will be independent from investigators and the sponsor	*Not applicable*
**Ethics and dissemination**			

For peer review only

Research ethics approval	#24	Plans for seeking research ethics committee / institutional review board (REC / IRB) approval	3
Protocol amendments	#25	Plans for communicating important protocol modifications (eg, changes to eligibility criteria, outcomes, analyses) to relevant parties (eg, investigators, REC / IRBs, trial participants, trial registries, journals, regulators)	*Not applicable*
Consent or assent	#26a	Who will obtain informed consent or assent from potential trial participants or authorised surrogates, and how (see Item 32)	9
Consent or assent: ancillary studies	#26b	Additional consent provisions for collection and use of participant data and biological specimens in ancillary studies, if applicable	See Appendix 4
Confidentiality	#27	How personal information about potential and enrolled participants will be collected, shared, and maintained in order to protect confidentiality before, during, and after the trial	13
Declaration of interests	#28	Financial and other competing interests for principal investigators for the overall trial and each study site	20
Data access	#29	Statement of who will have access to the final trial dataset, and disclosure of contractual agreements that limit such access for investigators	19
Ancillary and post trial care	#30	Provisions, if any, for ancillary and post-trial care, and for compensation to those who suffer harm from trial participation	*Not applicable*
Dissemination policy: trial results	#31a	Plans for investigators and sponsor to communicate trial results to participants, healthcare professionals, the public, and other relevant groups (eg, via publication, reporting in results databases, or other data sharing arrangements), including any publication restrictions	19
Dissemination policy: authorship	#31b	Authorship eligibility guidelines and any intended use of professional writers	1
Dissemination policy: reproducible research	#31c	Plans, if any, for granting public access to the full protocol, participant-level dataset, and statistical code	*Not applicable*

For peer review only

Appendices

Informed consent materials	#32	Model consent form and other related documentation given to participants and authorised surrogates	Appendix 4
Biological specimens	#33	Plans for collection, laboratory evaluation, and storage of biological specimens for genetic or molecular analysis in the current trial and for future use in ancillary studies, if applicable	*Not applicable*

None The SPIRIT checklist is distributed under the terms of the Creative Commons Attribution License CC-BY-ND 3.0. This checklist can be completed online using https://www.goodreports.org/, a tool made by the EQUATOR Network in collaboration with Penelope.ai

For peer review only
